# Determining the presence of asthma-related molecules and salivary contamination in exhaled breath condensate

**DOI:** 10.1186/s12931-017-0538-5

**Published:** 2017-04-12

**Authors:** Charmion Cruickshank-Quinn, Michael Armstrong, Roger Powell, Joe Gomez, Marc Elie, Nichole Reisdorph

**Affiliations:** Skaggs School of Pharmacy and Pharmaceutical Sciences, University of Colorado Anschutz Medical Campus, 12850 East Montview Boulevard, Aurora, CO 80045-2605 USA

**Keywords:** EBC, Saliva, Lung, Metabolomics, Proteomics, LC-MS, Amino acids, Eicosanoids, Leukotriene, Asthma

## Abstract

**Background:**

Researchers investigating lung diseases, such as asthma, have questioned whether certain compounds previously reported in exhaled breath condensate (EBC) originate from saliva contamination. Moreover, despite its increasing use in ‘omics profiling studies, the constituents of EBC remain largely uncharacterized. The present study aims to define the usefulness of EBC in investigating lung disease by comparing EBC, saliva, and saliva-contaminated EBC using targeted and untargeted mass spectrometry and the potential of metabolite loss from adsorption to EBC sample collection tubes.

**Methods:**

Liquid chromatography mass spectrometry (LC-MS) was used to analyze samples from 133 individuals from three different cohorts. Levels of amino acids and eicosanoids, two classes of molecules previously reported in EBC and saliva, were measured using targeted LC-MS. Cohort 1 was used to examine contamination of EBC by saliva. Samples from Cohort 1 consisted of clean EBC, saliva-contaminated EBC, and clean saliva from 13 healthy volunteers; samples were analyzed using untargeted LC-MS. Cohort 2 was used to compare eicosanoid levels from matched EBC and saliva collected from 107 asthmatic subjects. Samples were analyzed using both targeted and untargeted LC-MS. Cohort 3 samples consisted of clean-EBC collected from 13 subjects, including smokers and non-smokers, and were used to independently confirm findings; samples were analyzed using targeted LC-MS, untargeted LC-MS, and proteomics. In addition to human samples, an in-house developed nebulizing system was used to determine the potential for EBC samples to be contaminated by saliva.

**Results:**

Out of the 400 metabolites detected in both EBC and saliva, 77 were specific to EBC; however, EBC samples were concentrated 20-fold to achieve this level of sensitivity. Amino acid concentrations ranged from 196 pg/mL – 4 μg/mL (clean EBC), 1.98 ng/mL – 6 μg/mL (saliva-contaminated EBC), and 13.84 ng/mL – 1256 mg/mL (saliva). Eicosanoid concentration ranges were an order of magnitude lower; 10 pg/mL – 76.5 ng/mL (clean EBC), 10 pg/mL – 898 ng/mL (saliva-contaminated EBC), and 2.54 ng/mL – 272.9 mg/mL (saliva). Although the sample size of the replication cohort (Cohort 3) did not allow for statistical comparisons, two proteins and 19 eicosanoids were detected in smoker vs. non-smoker clean-EBC.

**Conclusions:**

We conclude that metabolites are present and detectable in EBC using LC-MS; however, a large starting volume of sample is required.

**Electronic supplementary material:**

The online version of this article (doi:10.1186/s12931-017-0538-5) contains supplementary material, which is available to authorized users.

## Background

Exhaled breath condensate (EBC) is comprised of volatile gases (e.g. nitric oxide) [1] and non-volatile compounds such as eicosanoids and cytokines [2]. EBC is increasingly used as a tool for biomarker discovery; it can be obtained non-invasively and reflects the physiology of the airway lining, thereby providing vital information about lung health. Publications have reported on the benefits of EBC as a quick screening tool for lung diseases such as asthma [3–6], pneumonia [5], chronic obstructive pulmonary disease (COPD) [5–8], cystic fibrosis [4, 9, 10], and pneumoconiosis [11]; these suggest the potential for EBC to be used in point-of-care diagnostics.

Several EBC studies have focused on eicosanoids due to their known relationship to lung disease and the fact that these compounds are released from mast cells and eosinophils during inflammatory responses [12]. Leukotriene B4 (LTB_4_), for example, has been shown to be released by activated alveolar macrophages in sarcoidosis patients [13]. Moreover, levels of cysteinyl-leukotrienes (CysLT) and 8-isoprostane have been reported to be increased in EBC of moderate and severe asthma patients compared to healthy controls [14]. Antczek et al. [15] investigated CysLT, LTB_4_, prostaglandin E4 (PGE_4_), and 8-isoprostane in longitudinal EBC samples of 16 COPD patients at 4 time points: day 1, during treatment, after therapy, and when stable. Their results showed (1) a decrease in CysLT, LTB_4_, and 8-isoprostane after antibiotic therapy and (2) that eicosanoids were elevated in the airways of stable COPD patients compared to healthy subjects. Although numerous studies demonstrate the potential for EBC in point-of-care diagnostics, eicosanoid detection in EBC can be marked by loss of analyte due to adsorption in plastic collection tubes. Therefore, recent investigators have examined the use of glass tubes coated with surfactants, such as Tween 20, as an alternative to improve eicosanoid analysis in EBC [16].

In addition to eicosanoids, other EBC studies have focused on the measurement of nitrogen oxide [17], glucose [10], proteins [18], and amino acids [19]. Amino acids have been shown to be markers of lung function [20], are dysregulated in COPD [21, 22], and are perturbed in smokers [23]. Although amino acids and eicosanoids have been reported in EBC [19, 24, 25], the contribution from saliva remains in question. Saliva contains more than 200 metabolites [26, 27] and has been suggested as a source of contamination in EBC during sample collection. Of the EBC collection devices commercially available, only two (ECoScreen, and TURBO-DECCS) contain saliva traps [28].

Because there is potential for saliva contamination in EBC, many investigators test for saliva contamination by measuring hydrolytic α-amylase activity. Syslova et al [29] investigated CysLTs using a rapid method comprising pre-concentration, stable isotope dilution, and immunoaffinity. Since the α-amylase activity in samples did not exceed 0.1% of the saliva activity, the investigators excluded significant salivary contamination of EBC. However, small amounts of saliva molecules can be detected by liquid chromatography tandem mass spectrometry (LC-MS/MS); Gaber et al reported that the main source of LTB_4_ detected in EBC was from saliva [25]. LTB_4_ and α-amylase activity were measured in saliva and α-amylase activity was measured in undiluted EBC. The authors observed that spiking EBC with saliva consistently increased LTB_4_ levels in EBC and concluded that α-amylase assay may not be sufficiently sensitive to show the presence of saliva in small quantities.

We sought to define the usefulness of EBC in investigating lung disease by characterizing its constituents and delineating if the molecules reportedly detected in EBC are a result of saliva contamination during sample collection. This was achieved using three strategies. First, we used an in-house developed nebulizing system to validate methods and to determine loss of molecules during collection. Second, since they have been reported as increased in lung disease, we specifically measured amino acids and eicosanoids in saliva and EBC in both healthy and asthmatic individuals. Finally, we used untargeted metabolomics and proteomics to determine the components of a highly concentrated EBC sample.

## Methods

### Study population

Adults were recruited from asthmatic patients at National Jewish Health through flyers and through the asthma clinic. Studies were approved by the Western Institutional Review Board or National Jewish Health IRB. Informed consent was obtained from all participants. The 133 volunteers were adult males and females ranging in age 27-64 years old; subjects filled out questionnaires detailing the last time they ate, drank, brushed their teeth, flossed, or used mouthwash.

Cohort 1: This cohort included 13 healthy subjects with no pre-existing conditions who provided clean-EBC, saliva-EBC, and/or clean saliva. Not all subjects were able to provide both EBC and saliva: 80% of subjects who provided clean-EBC provided saliva; 100% of subjects who provided saliva-EBC also provided saliva; 5 subjects who provided saliva did not provide EBC.

Cohort 2: This cohort consists of 107 asthmatic subjects who had matched saliva and EBC collected. One subject who provided EBC did not provide saliva.

Cohort 3: This cohort refers to a separate group of 13 subjects of various health statuses – healthy smoker, healthy non-smoker, common cold, nasal congestion – who all provided clean-EBC. The smoker group is designated as smoking at least one cigarette per day, while healthy indicated no other pre-existing conditions.

Additional demographic data is unavailable for the cohorts as patient samples were de-identified and additional information was no longer available following completion of the study. Because only minimal information was available, no statistical claims are being made based on health status, gender, or age in these cohorts.

### Chemicals and reagents

All solvents for untargeted mass spectrometry were LC-MS grade. Acetonitrile, methanol, and formic acid were purchased from Fisher Scientific (Fairlawn, New Jersey); Water was purchased from Honeywell (Muskegon Michigan); 1-methylhistidine, 3-methylhistidine, α-amino-n-butyric acid, alanine, anserine, arginine, asparagine, aspartic acid, β-aminoisobutryic acid, β-alanine, carnosine, citrulline, creatinine, cystathionine, cysteine, ethanolamine, γ-aminobutyric acid, glutamic acid, glutamine, glycine, histidine, homocystine, hydroxylysine, hydroxyproline, isoleucine, L-aminoadipic acid, L-cystine, leucine, lysine, proline, methionine, ornithine, phenylalanine, phosphoserine, phosphoethanolamine, sarcosine, serine, taurine, threonine, tryptophan, tyrosine, urea, and valine were purchased from Sigma Aldrich (St. Louis, Missouri); 10(S),17(S)-DiHDoHE (Protectin DX), 11β-PGF_2α_, 14(S)-HDHA, 15R-PGF_2α_, 17(S)-HDHA, 8-iso-15R-PGF_2α_, 8-iso-PGF_2α_, Lipoxin A4 (LXA_4_), LTB_4_, LTC_4_, LTD_4_, LTE_4_, PGE_2_, PGF_2α_, Resolvin D1 (RVD1), and Resolvin D2 (RVD2) were purchased from Cayman Chemical (Ann Arbor, Michigan).

All standards and deuterated internal standards used for LC-MS/MS analysis of arachidonic acid, docosahexaenoic acid derived lipid mediators were purchased from Cayman Chemical (Ann Arbor, Michigan, USA). All HPLC solvents and extraction solvents were HPLC grade or better.

### EBC spike recovery experiments: evaluation of adsorption to glass versus plastic tubes

To evaluate adsorption of eicosanoids during collection and analysis, simulated experiments were conducted with different types of collection tubes and analysis was performed with two EBC devices to determine compound adsorption to these different tubes. Glass and plastic collection tubes were tested with the RTube device (a disposable collection system which separates saliva from the exhaled breath) (Respiratory Research, Inc., Charlottesville, VA). Glass with polyethylene terephthalate (PET), glass without PET and plastic without PET were tested with the TURBO DECCS device (a transportable unit for use in research on biomarkers obtained from disposable exhaled condensate collection systems) (Medivac, Italy). EBC production and deposition was simulated using the EBC sampling devices without human subjects.

#### RTube EBC simulation standards

The deuterated internal standard solution contained LTB_4_-d4, LTE_4_-d3 and PGE_2_-d4 at concentrations of 500 pg/ml in ethanol and stored at -20 °C until use. The calibration standards (LTB_4_, LTC_4_, LTD_4_, and LTE_4_) were prepared in LC-MS water to final concentrations of 5, 10, 25, 50, 100, and 250 pg/mL and they were kept on ice until use. Standards were prepared for analysis by adding 200 μL of deuterated internal standard solution and 800 μL of calibration standard into a 2 mL autosampler vial and vortexing for 5 s. The 10 pg/mL (low level) and 100 pg/mL (high level) calibration standards were used for infusion in the EBC simulation experiment.

#### RTube EBC simulation setup

The RTube was constructed with an alternative material (Borosilicate glass) and the performance compared with the plastic RTube. The EBC simulation apparatus utilized the following components: syringe pump, a plastic 10 mL luer lock BD syringe, a 2 foot section of 0.17 mm PEEK tubing with an inline 2 μm frit and luer lock adapter, an Agilent electrospray nebulizer and column stand, nitrogen (98% pure or better) supplied at 10psi, an aluminum condenser (for plastic RTubes) stored at -80 °C, and water ice condenser fabricated with a zip-lock bag (for glass RTubes) stored at -80 °C.

Before each simulation, the syringe, tubing and nebulizer assembly was rinsed with 200 μL of LC-MS methanol. 8-9 mL of low or high level standard was poured into the 10 mL syringe and placed into the syringe pump. The syringe was attached to the tubing and nebulizer. The gas port of the nebulizer was blocked with a blank nut. The syringe pump was set to pump 1.5 mL of standard solution for 5 min (0.3 mL/min). A first 5 min infusion was run and discarded to purge the line; a second 5 min infusion was run and collected in a 2 mL glass autosampler vial as a control sample. Nitrogen at 10 psi was then attached to the nebulizer. The syringe pump was operated for 20 s to observe and verify the nebulizer spray quality. A glass or plastic RTube was attached to the column stand. The nebulizer was then attached to the column stand with the nebulizer tip protruding 1-5 mm above the RTube duckbill.

The appropriate condenser was slid over the RTube and careful observation was made to ensure that the nebulizer needle was centered in the duckbill. The syringe pump was allowed to flow for 5 min. The glass RTube required a small amount of isopropanol (20-50 μL) to be injected with a spinal needle between the bottom of the duckbill and the walls of the condenser to prevent the duckbill from sticking to the walls during the EBC recovery. Condensate was aliquoted into a 2 mL glass autosampler vial and placed on ice. Samples were prepared for analysis by adding 800 μL of condensate and 200 μL of deuterated internal standard to a 2 mL glass autosampler vial and vortexing for 5 s.

#### TURBO DECCS EBC simulation standards

Standards were prepared using the same protocol as the RTube experiments with the following changes: calibration standards were prepared in LC-MS water to final concentrations of 1, 5, 10, 25, 50 pg/mL and kept on ice until use. All calibration standards were infused and collected in autosampler vials using the EBC simulation apparatus without nebulizer gas. Three additional aliquots of the 10 pg/mL standard were prepared for the EBC simulation experiments.

#### TURBO DECCS EBC simulation collection

EBC simulation apparatus was the same as the RTube setup with the following changes: the 0.12 mm peek tubing was replaced with 0.17 mm stainless steel tubing to reduce back pressure on the syringe pump. Nitrogen was supplied at 60 psi which produced a finer spray to help prevent condensation in the PET tube. Before each simulation, the syringe, tubing and nebulizer assembly was rinsed with 200 μl of LC-MS methanol. The disposable DECCS sampling assembly was placed into the Turbo cooler and allowed to reach -5 °C +/-0.5 °C. 8-9 mL of low or high level standard was poured into the 10 mL syringe and placed into the syringe pump. The syringe was attached to the tubing and nebulizer. The gas port of the nebulizer was blocked with a blank nut. The syringe pump was set to pump 2 mL of standard for 7.5 min (0.3 mL/min). The first 2 min of the infusion sample were discarded. The next three 2.0 mL infusions were collected in a 2 mL autosampler vial and set aside in 4 °C fridge until ready to aliquot for analysis. Nitrogen at 60 psi was then attached to the nebulizer. The syringe pump was operated for 20 s to observe and verify the nebulizer spray quality. The nebulizer was then placed into the assembly and the syringe pump was programmed to run for 10 min (infuse 3 mL of condensate). At the completion of the condensate collection, the 50 mL collection tube was centrifuged for 2 min and the condensate was aliquoted into a 2 mL autosampler vial for analysis.

An initial experiment with the standard DECCS sampling device showed excessive adsorption of cysteinyl leukotrienes. The source of the adsorption was determined using 3 separate experimental setups (*n* = 3 for each parameter). Setup #1: DECCS assembly minus the mouthpiece with a solvent-rinsed 30 mL glass Corex centrifuge tube placed inside the 50 mL plastic collection tube; Setup #2: Same as setup #1 but minus the PET tube. The nebulizer was placed directly on top of the diffusing tube and the collecting tube; Setup #3: Same as setup #2 minus the 30 mL glass Corex centrifuge tube. These different parameters allowed the elimination of the PET tube and the 50 mL collection tube as possible sites of adsorption for the Cys-LTs.

### Saliva sample collection and preparation

Each volunteer from Cohort 1 was provided with a 50 mL conical tube and saliva was allowed to flow naturally into the tube for 15-30 min. Approximately 3-5 mL of saliva was collected per volunteer. Samples were collected between 9:45 am and 10:30 am, approximately 2-3 h after eating breakfast. For Cohort 2, a “dirty” saliva sample was collected by having the subject spit into a 15 mL conical tube without rinsing their mouth. The subject then rinsed their mouth out 3 times with drinking water. A “clean” saliva sample was produced by having the subject chew a piece of chewing gum for 15-30 s, removing gum, and then spitting into 15 mL conical falcon tube; subjects were cautioned not to swish the sample in their mouth prior to spitting.

Immediately after collection, the saliva in the falcon tube was then centrifuged for 10 min at 3000 rpm at 4 °C to separate the clear liquid component of the saliva from the stringy mucus portion of the sample. The supernatant was pipetted into an amber autosampler vial for untargeted LC-MS analysis. For targeted analysis, 1 mL of the centrifuged saliva was placed into a new falcon tube containing 50 μL of internal standard (LTB_4_-d4, LTC_4_-d5, LTD_4_-d5, LTE_4_-d5 and PGE_2_-d4 at 2.5 pg/μL in ethanol) and 4 mL of acetonitrile. Samples were then vortexed for 10 s. After addition of the acetonitrile to the clarified saliva, some additional stringy mucus may appear, so the samples were then allowed to settle. Taking care not to disturb the stringy mucus, 4 separate aliquots of the sample were pipetted into 1.5 mL centrifuge tubes and centrifuged at 14,000 rpm for 10 min at 4 °C. 950 μL of supernatant was removed and placed in a new centrifuge tube. The sample was then frozen at -80 °C until analysis was performed. Prior to MS analysis, samples were dried in the centrifugal evaporator until ~200 μL of sample remained. The remaining supernatant was transferred to a 1.8 mL glass autosampler vial. 200 μL of ethanol was added to the centrifuge tube and vortexed for ~5 s. The ethanol was transferred to the vial with the rest of the supernatant. The sample was then diluted to 1 ml with LC-MS water and analyzed.

### EBC sample collection and preparation

#### EBC control experiment

A TURBO-DECCS (Medivac, Italy) apparatus was set up to mimic a human subject breathing into an EBC collection tube (Fig. 2a) and included a saliva trap. A syringe was rinsed thoroughly with LC-MS grade water followed by LC-MS grade methanol and then attached to a syringe pump at one end. At the other end, the syringe was connected to the mouthpiece and secured with parafilm. The syringe pump flow rate was adjusted to allow 3 mL of sample to be collected in 15 min (average amount of EBC per person). A nitrogen dryer was attached to the side of the mouthpiece using tubing to mimic exhalation. Two blank samples were run through the device using 100% LC-MS grade water; sample was collected for analysis and referred to as the ‘blank water control’. This was followed by running spiked LC-MS grade water containing 42 amino acids (10 μM) and 16 eicosanoids (10 ng/mL) as a spiked control; this sample was designated ‘spiked water control’. A new TURBO-DECCS nozzle and collection tube was used for each sample. Each nozzle and tube was discarded after use.

#### EBC sample collection and preparation

The TURBO-DECCS collection device (Medivac, Italy) was set up at room temperature and the condenser temperature was allowed to decrease until stable at -5.5 °C. This EBC collection device contains a saliva trap. A new, unused mouth piece and nozzle was supplied for each individual. Nose clips were optional. EBC was collected per ATS/ERS recommendations [30]. Subjects breathed normally into the EBC breathing apparatus for 15-20 min each. Subjects did not eat, drink, or exercise for at least two hours prior to sample collection. Approximately 2-5 mL of EBC was collected per person. For Cohort 1, samples were examined for possible salivary contamination and marked as “saliva” or “clean”. Human-derived EBC samples were pooled into two groups (‘clean’ EBC, 12.5 mL, (*n* = 5 subjects), and ‘saliva’ EBC, 8.5 mL (*n* = 3 subjects)) as shown in Fig. 2e, and aliquoted into 15 mL falcon tubes. Human-derived EBC samples and control EBC samples (blank water control and spiked water control) were immediately frozen at -80 °C overnight, and lyophilized the following morning.

The lyophilized samples were each reconstituted in 1 mL of 80:20 methanol: water, briefly vortexed and centrifuged. EBC samples were then dried down in a vacuum centrifugal concentrator at 55 °C for 1.5 h, reconstituted in 20 μL of LC-MS starting buffer (2% of 90:10 acetonitrile:water with 0.1% formic acid), spun for 5 s to remove residue from the sides of the centrifuge tube, and transferred to an amber autosampler vial. The entire amount of each human-derived and control EBC sample was used for untargeted LC-MS analysis. For targeted analysis, immediately after collection, 400 μL of internal standard (LTB_4_-d4, LTC_4_-d5, LTD_4_-d5, LTE_4_-d5 and PGE_2_-d4 at 0.125 pg/μL in ethanol) was added to the EBC, vortexed and then centrifuged for 10 minutes at 3000 rpm at 4 °C. The total condensate volume was measured and recorded and split evenly between 2 centrifuge tubes. LC-MS water was added to each tube to reach a final volume of 1 mL. The samples were then frozen at -70 °C until analysis. Note that a saliva trap was used to prevent saliva contamination in EBC samples in Cohort 3 as well as visual observation of each sample following collection. Water spiked with amino acids and eicosanoids (referred to as standards solution) was used as reference. Overall, samples were concentrated 50-fold and divided into aliquots for untargeted LC-MS, targeted LC-MS, and proteomics analysis.

### Untargeted metabolomics analysis

#### Untargeted LC-MS

Liquid chromatography was performed on an Agilent Series G2226A pump by injecting 5 μL sample onto an Agilent Zorbax SB-Aq Rapid Resolution HT 2.1 x 100 mm, 1.8 μm, 600 bar analytical column, coupled to an Agilent Zorbax SB-Aq Narrow-Bore 2.1 x 12.5 mm, 5 μm guard column. The autosampler tray temperature was set at 4 °C and column compartment was set at 30 °C. Samples were run at a flow rate of 0.3 mL/min, using mobile phase A (water with 0.1% formic acid), and mobile phase B (90:10 acetonitrile:water with 0.1% formic acid). Gradient elution was as follows: 0-3 min 2% B, 3-5 min 2-40% B, 5-20 min 40-100% B, 20-30 min 100% B, followed by column re-equilibration. An Agilent 6520 quadrupole time-of-flight mass spectrometer (Q-TOF-MS) was used to analyze samples in positive and negative ionization mode at mass range 50-1700 m/z, scan rate 2.22 spectra/s, gas temperature 300^o^C, and gas flow 10 L/min. The nebulizer was 30 psi, skimmer 60 V, capillary voltage 4000 V, and fragmentor 120 V in positive mode and 140 V in negative mode, with reference masses 121.050873 and 922.009798 for positive mode and 112.985628 and 966.000725 for negative mode (Agilent reference mix).

#### Quality control

To reduce false positives due to cross contamination or carryover, a new column was used. Instrument blanks (100% methanol) were injected onto a new column and followed by ‘water blanks’ prior to sample analysis. Samples were run in the following order: control “blank” water, clean-EBC, saliva-EBC, spiked water control sample. Solvent blanks were run between each sample to eliminate carryover. Saliva samples were run last, followed by a series of additional blanks and laboratory instrument QCs to ensure that the instrument was operating at optimal conditions. This process greatly reduced the chances of any potential carryover from any of the samples.

#### Data extraction and analysis

MassHunter Profinder software (Agilent) was used to analyze the spectral data. An initial naïve feature finding algorithm was used to detect and extract peaks present in the spectrum of the samples using the following parameters: peak heights ≥ 300 counts, ion species + H, +Na, +K in positive mode, -H, +Br, +HCOO, +CH_3_COO in negative mode, charge state maximum of 2, ion threshold of two or more ions, alignment using 0.3 min retention time and 20 ppm mass window, absolute height ≥ 1100 counts, MFE score ≥ 90, and a compound must be present in at least two sample files. A formula generation algorithm was used to re-mine the spectral data and reduce missing values using the following parameters: symmetric ppm of 20, matching score > 75, absolute peak height ≥ 1000 counts, absolute ion filter height ≥ 1000 counts, and a compound must be present in at least two sample files. Compounds were imported into Mass Profiler Professional (Agilent) using a minimum abundance threshold of 3000 counts. Solvent blanks and the unspiked water controls were background subtracted to eliminate any contamination from the samples during sample collection, preparation or instrument analysis; this removed 13 compounds in control water and 2 compounds in the instrument blank from the EBC samples.

#### MS/MS analysis

Tandem MS was performed on an Agilent 6560 IMMS Q-TOF for selected metabolites. MS/MS data was collected with a 500 ms/spectra acquisition time. Precursor ions were isolated with a 4 *m/z* isolation width and 1 min delta retention time. Collision energies of 10, 20, and 40 eV were applied. Fragmentation data was exported to the freely available NIST MS Search v.2.2 g GUI program [31] (NIST, Gaithersburg, MD, USA) and were matched to spectra in the NIST 14 Mass Spectral Library. This library contains 193,119 spectra representing 43,912 precursor ions and 8,351 compounds; a detailed description of the library is available [32]. Automated library searching was performed using spectrum search type ‘Identity’, search with “MS/MS”, and default program settings. The search *m/z* tolerance was ± 0.4 for precursor ions and ± 0.4 for product ions without ignoring the precursor ion. The MS search program outputted a list of matched chemical compounds including several measures of spectral similarity [33]. The Match Factor (MF) is the normalized dot product with square-root scaling of the experimental mass spectrum and a library mass spectrum, using all the elements in the experimental mass spectrum. The Reverse Match Factor (RMF) is the normalized dot product with square-root scaling of the experimental mass spectrum and the library mass spectrum, but the elements that are not present in the library mass spectrum are not included.

#### Amino acid and eicosanoid data analysis

The raw LC-MS .d files from the standards and samples were imported into MassHunter Quantitative Analysis Software (v.B.07.00) (Agilent Technologies, Santa Clara, CA) to extract m/z and retention times using tolerances of ±0.5 min for retention time window and ±10 ppm for m/z. Quantitation of amino acids and eicosanoids was based on peak areas of amino acids and eicosanoids in the samples against known spiked standards [34, 35].

#### Compound identification

Amino acids and eicosanoids in EBC and saliva were identified by matching their exact mass, isotope ratios, and retention times to purchased standards. An in-house database comprising METLIN, LIPID MAPS, Kyoto Encyclopedia of Genes and Genomes (KEGG), and human metabolome database (HMDB) was used to annotate additional detected metabolites in the untargeted data using exact mass, isotope ratio, and isotopic distribution with a mass error of ≤10 ppm and a minimum database score of 70/100. Spectra were manually inspected for quality.

### Targeted analysis of lipid mediators by LC-MS

Quantitation of lipid mediators was performed using 2 dimensional reverse phase HPLC tandem mass spectrometry (LC-MS/MS). The HPLC system consisted of an Agilent 1260 autosampler (Agilent Technologies, Santa Clara, CA), an Agilent 1260 binary loading pump (pump 1), an Agilent 1260 binary analytical pump (pump 2) and a 6 port switching valve. Pump 1 buffers consisted of 0.1% formic acid in water (solvent A) and 9:1 v:v acetonitrile:water with 0.1% formic acid (solvent B). Pump 2 buffers consisted of 0.01% formic acid in water (solvent C) and 1:1 acetonitrile:isopropanol (solvent D).

100 μl of extracted saliva or EBC was injected onto an Agilent Poroshell EC-C18 2.1x5 mm 2.7 μm trapping column using pump 1 at 0.5 mL/min for 0.5 min with a solvent composition of 95% solvent A: 5% solvent B. At 0.51 min the switching valve changed the flow to the trapping column from pump 1 to pump 2. The flow was reversed and the trapped lipid mediators were eluted onto an Agilent Poroshell EC-C18 2.1x150 mm 2.7 μm analytical column using the following gradient at a flow rate of 0.4 mL/min: hold at 75% solvent A:25% solvent D from 0-0.5 min, then a linear gradient from 25-45% D over 8.5 min followed by an increase from 45-48% D from 8.5-11 min, then from 48% D to 65% D over 4 min, and then from 65-100% D in 0.01 min, finally holding at 100% D for 1 min. During the analytical gradient pump 1 washed the injection loop with 100% B for 4 min at 0.5 mL/min. Both the trapping column and the analytical column were re-equilibrated at starting conditions for 4 min before the next injection.

Mass spectrometric analysis was performed on an Agilent 6490 triple quadrupole mass spectrometer in negative or positive ionization mode, based on compound chemistry (Additional file 1). The drying gas was 250 °C at a flow rate of 15 mL/min. The sheath gas was 350 °C at 12 mL/min. The nebulizer pressure was 35 psi. The capillary voltage was 3500 V in negative mode and 4000 V in positive mode.

Data for lipid mediators was acquired in dynamic MRM mode using experimentally optimized collision energies obtained by flow injection analysis of authentic standards (Additional file 1). Calibration standards for each lipid mediator were analyzed over a range of concentrations from 0.04 pg/mL-8 pg/mL. Calibration curves for each lipid mediator were constructed using Agilent MassHunter Quantitative Analysis software. The results were calculated by obtaining the ratio of the target compound/internal standard and then using the linear equation obtained from the calibration curve (y = mX + b) to get the final concentration in pg/mL.

### Proteomics analysis

An aliquot of the EBC samples collected from the 13 volunteers in the replication study (Cohort 3) and grouped into 4 categories based on health status was used for proteomics analysis; 120 μL healthy non-smoker, 8 μL healthy smoker, 28 μL non-smoker common cold, and 6.6 μL non-smoker nasal congestion. Samples were dried in a centrifugal evaporator at 45 °C. The 4 samples then underwent a trifluoroethanol (TFE) in-solution digestion overnight with trypsin. Digests were dried at 45 °C and resuspended in 10 μL of 3% acetonitrile with 0.1% formic acid.

5 μL of each sample was injected onto a ProntoSil C18AQ (0.1X150mm) column from NanoLCMS Solutions. Samples were analyzed using a nanoAdvance nano flow LC (Bruker) on the front of an Impact HD Q-TOF (Bruker) with a gradient elution from 5-50% over 30 min at 40 °C and 800 nL/min flow rate. Buffer A was water with 0.1% formic acid, and buffer B was acetonitrile with 0.1% formic acid. Data was acquired at 2Hz over a range of 150-2200 m/z. Data was processed using DataAnalysis 4.2 (Bruker), database searches were performed with Mascot v2.4 (Matrix Science), and protein assessment/scoring was performed with ProteinScape 3.1 (Bruker).

## Results

### Experimental set up for control experiments

**Fig. 1:**
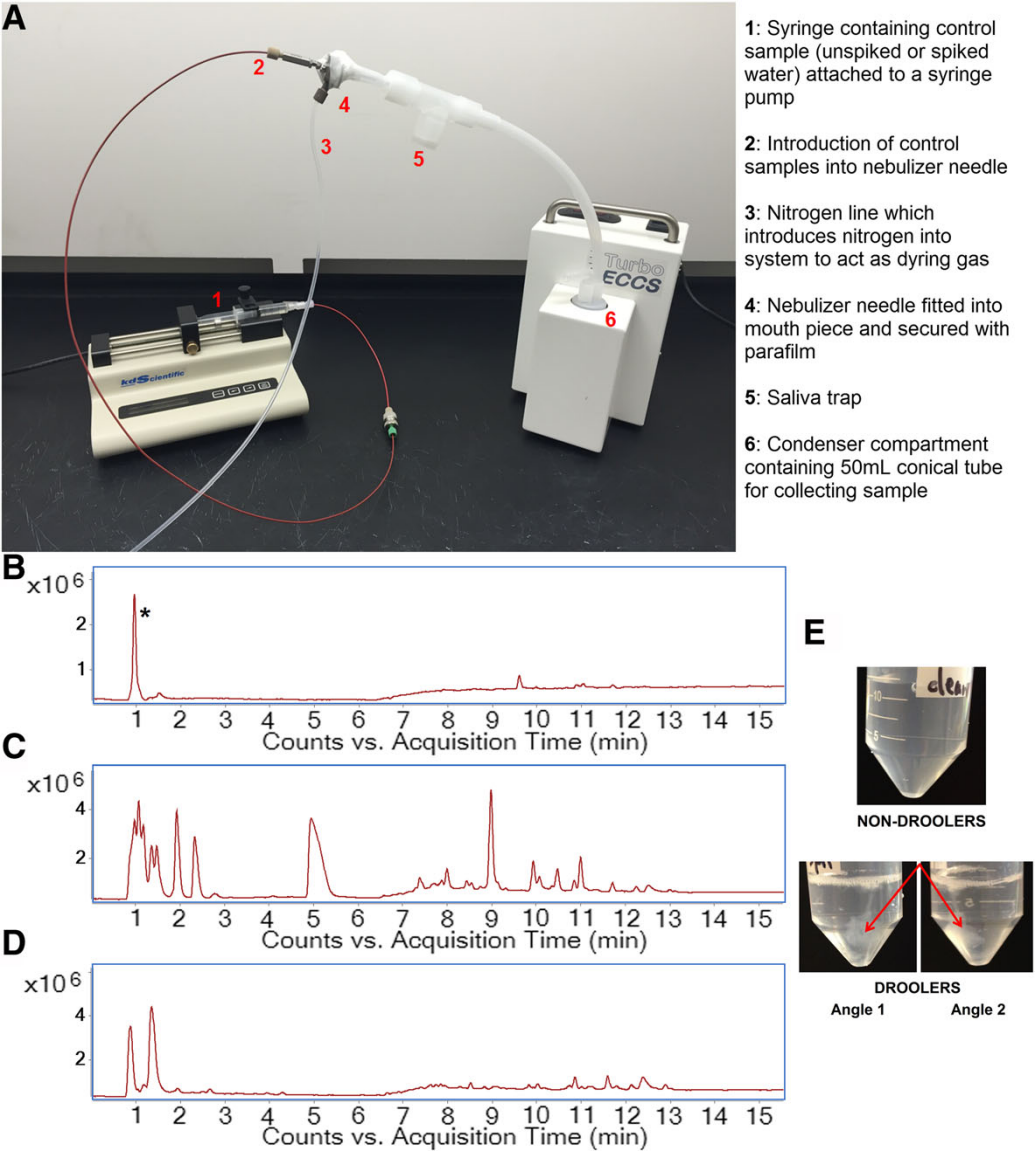
Experimental setup and total ion chromatograms (TIC) of exhaled breath condensate (EBC) controls. **a** Setup of the control experiment using a syringe and syringe pump to administer control unspiked water and a control spiked water sample through the TURBO-DECCS EBC collection apparatus; **b** TIC of unspiked water. * indicates a contaminant peak at 0.933 min which was putatively identified as propiolic acid (8.69 ppm error, 82.8 score), **c** TIC of spiked water; **d** TIC of a ‘clean’ EBC sample; **e** Visualization of EBC samples collected from volunteers showing the clean EBC from non-droolers, compared to EBC collected from droolers which show the presence of saliva in the samples

In order to test recoveries and determine background contamination, a system was developed that mimicked EBC collection. As shown in Fig. 1a, a syringe and syringe pump were attached to a nebulizer; this was used to administer sample at a controlled rate into a TURBO-DECCS EBC collection apparatus. An unspiked, “blank” water sample was used as a control to monitor contaminants that may be present in the collection tubing. Water spiked with amino acids and eicosanoids was used to measure recoveries; this “spiked control” was also used as a reference to verify metabolite identities in EBC using mass and retention time. As expected, no compounds were detected in the “blank” controls. In total, 30 out of the 42 spiked amino acids and 16 spiked eicosanoids were detected in the “spiked control” samples. Following method validation, EBC (*n* = 8) was collected from healthy volunteers, and underwent minimal sample preparation to reduce potential for contamination. EBC was divided into clean (*n* = 5) and saliva-contaminated (*n* = 3) based on visual inspection of the samples. Note that spikes were not added to the EBC samples; this was in an effort to reduce false positives that may be present as a result of degradation of spiked standards. Figure 1b, c and d show representative total ion chromatograms (TIC) of the blank, spiked water and “clean” EBC samples respectively. Figure 1e shows EBC in tubes following collection; saliva-contaminated EBC was clearly distinguishable from non-contaminated EBC.

### Adsorption of compounds on EBC collection tubes: leukotriene recovery experiment

**Fig. 2:**
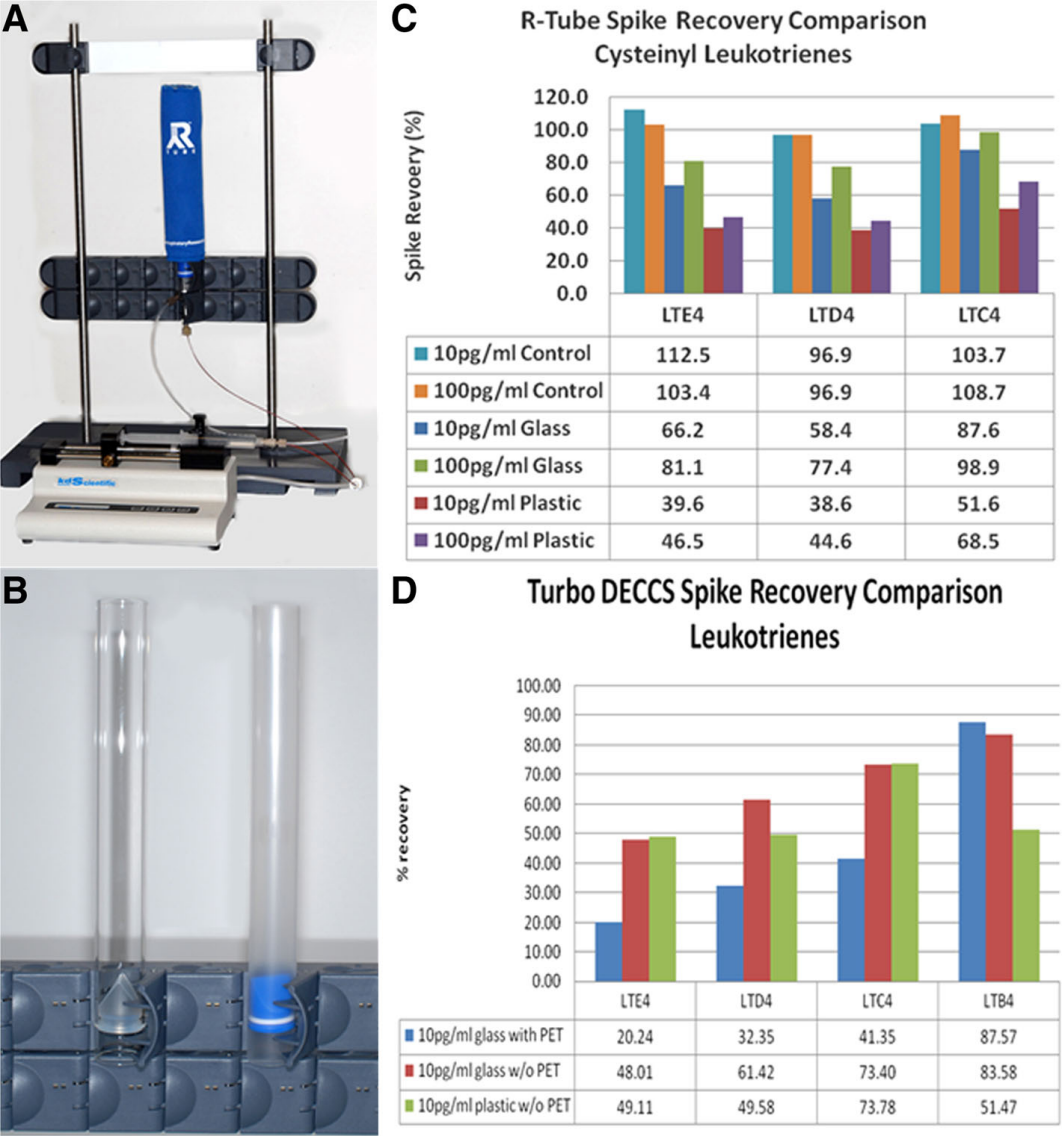
Experimental setup and recoveries of leukotrienes using various exhaled breath condensate (EBC) collection devices. **a** EBC simulation device connected to a syringe pump, showing the plastic tube and condenser; **b** Glass (left) versus plastic (right) EBC tube; **c** RTube spike recovery comparison for cysteinyl leukotrienes; **d** TURBO-DECCS spike recovery comparison for leukotrienes. PET: polyethylene terephthalate

A second control experiment (Fig. 2) was performed to determine recoveries of commonly reported EBC molecules. Because plastic tubes may result in adsorption of certain molecules, glass and plastic tubes and the addition of a coating were compared. For this experiment, known amounts of leukotrienes (10 pg/mL and 100 pg/mL) were injected into TURBO DECCS tubes using the apparatus in Fig. 1; RTubes were attached to the syringe pump as shown in Fig. 2. Overall, recoveries of the leukotrienes ranged between 96.9 – 112% depending on the collection tube used (Fig. 2c). Recoveries were higher with the glass RTube device (58.4 – 98.9%) compared to the plastic RTube device (38.6 – 68.5%). In experiments using the TURBO DECCS, recoveries were higher using the glass tube without PET (48.01 – 83.58%), compared to the glass tube with PET (20.24 – 87.57%), and the plastic tube without PET (49.11 – 73.78%). These recovery experiments showed significant adsorption of cysteinyl leukotrienes to the plastic RTube by ~60% compared to the glass RTube (Fig. 2c). There was also 10-22% less adsorption of the leukotrienes to the TURBO-DECCS plastic tube compared to the plastic RTube at the 10 pg/mL spike levels (Fig. 2d). In addition, polyethylene terephthalate (PET) caused significant adsorption of LTC_4_, LTD_4_, and LTE_4_ to the coated collection tubes compared to the uncoated tubes (Fig. 2d).

### Comparison of eicosanoids and amino acids in healthy saliva, healthy EBC, and healthy saliva-EBC

Saliva and EBC were collected from 13 healthy subjects (Cohort 1) and analyzed using LC-MS. Table 1 shows concentrations of the amino acids and eicosanoids detected in EBC and saliva of these subjects. Additional file 2 shows the extracted peak areas of selected compounds and Additional file 3 shows the separation of PGF_2α_isomers. Three amino acids (anserine, hydroxyproline, and cysteine) were undetected in all samples including the spiked water; these molecules may be below our limit of detection or are not detectable in our system (for example due to inability to ionize). Carnosine, homocystine, lysine, methionine, and phosphoethanolamine were only detected in the spiked water; this indicates that their concentration in EBC and saliva were below detection limits or that they may not be present in these biological fluids. Seven eicosanoids (protectin DX, 17(S)-HDHA, LTC_4_, LTD_4_, LTE_4_, RvD1, and RvD2) were less than half the concentration in clean-EBC compared to saliva-EBC; they were an order of magnitude lower in concentration in clean-EBC compared to saliva. Overall, in the clean-EBC, the eicosanoids were present at concentrations ranging 10 pg/mL – 76.5 ng/mL; amino acids ranged from 196 pg/mL – 4 μg/mL.

**Table 1:** Amino acid and eicosanoid concentrations in healthy human saliva and healthy EBC

	Compound	Formula	Identifier	Clean-EBC(ng/mL)	Saliva-EBC (ng/mL)	Saliva (ng/mL)
Amino acids	1-methylhistidine/3-methylhistidine	C7H11N3O2	70958/70959	518.0	1276.1	1 256 059
	α-amino-n-butyric acid/β-aminoisobutryic acid	C4H9NO2	35621	316.3	41.3	90 579
	Alanine/β-Alanine	C3H7NO2	16449	58.1	51.9	13.84
	Anserine^a^	C10H16N4O3	18323	ND	ND	ND
	Arginine	C6H14N4O2	29016	1211.6	6016.8	101 238
	Asparagine	C4H8N2O3	22653	ND	8.6	7575
	Aspartic acid	C4H7NO4	22660	10.7	19.4	11 387
	Carnosine^a^	C9H14N4O3	15727	ND	ND	ND
	Citrulline	C6H13N3O3	18211	302.2	304.8	16 922
	Creatinine	C4H7N3O	16737	237.5	396.9	88 368
	Cystathionine	C7H14N2O4S	17755	930.7	1756.2	ND
	Cysteine^a^	C3H7NO2S	15356	ND	ND	ND
	Ethanolamine	C2H7NO	16000	52.5	230.9	20 887
	ϒ-aminobutyric acid	C4H9NO2	16865	41.3	56.1	13 705
	Glutamic acid	C5H9NO4	18237	77.0	29.6	49 725
	Glutamine	C5H10N2O3	28300	24.6	162.4	18 423
	Glycine	C2H5NO2	15428	5.99	11.4	1739
	Histidine	C6H9N3O2	27570	401.2	316.5	14 751
	Homocystine^a^	C4H9NO2S	17485	ND	ND	ND
	Hydroxylysine	C6H14N2O3	60175	205.6	283.3	133 890
	Hydroxyproline^a^	C5H9NO3	24741	ND	ND	ND
	L-Aminoadipic acid	C6H11NO4	37024	373.3	696.5	27 008
	L-Cystine	C6H12N2O4S2	17376	55.6	295.2	60 301
	Leucine/Isoleucine	C6H13NO2	25017/24898	100.2	507.1	99 237
	Lysine^a^	C6H14N2O2	18019	ND	ND	ND
	Methionine^a^	C5H11NO2S	16643	ND	ND	ND
	Ornithine	C5H12N2O2	18257	20.3	167.8	71 403
	Phenylalanine	C9H11NO2	28044	0.196	78.6	12 685
	Phosphoethanolamine^a^	C2H8NO4P	36711	ND	ND	ND
	Phosphoserine	C3H8NO6P	37712	685.1	3926.3	329 903
	Proline	C5H9NO2	26271	111.4	1088.9	25 577
	Sarcosine	C3H7NO2	16511	78.0	143.8	56 894
	Serine	C3H7NO3	17822	2.17	1.98	26 140
	Taurine	C2H7NO3S	15891	ND	405.7	53 156
	Threonine	C4H9NO3	26986	4055.0	6.67	1223
	Tryptophan	C11H12N2O2	27897	431.3	245.9	30 868
	Tyrosine	C9H11NO3	18186	ND	27.1	12 607
	Valine	C5H11NO2	27266	57.3	109.8	25 238
	Urea (negative mode)	CH4N2O	16199	1.56	51.4	3651
	Urea (positive mode)	CH4N2O	16199	2.22	65.1	5802
Eicosanoids	10(S),17(S)-DiHDoHE (Protectin DX)	C22H32O4	871826-47-0	0.084	0.176	166.83
	11β-PGF_2α_	C20H34O5	27595	76.5	9.60	ND
	14(S)-hydroxy Docosahexaenoic Acid	C22H32O3	119433-37-3	24.9	6.65	30.76
	15R-PGF_2α_	C20H34O5	37658-84-7	74.8	9.60	ND
	17(S)-hydroxy Docosahexaenoic Acid	C22H32O3	155976-53-7	1.07	4.28	798.09
	8-iso-15R-PGF_2α_	C20H34O5	214748-65-9	69.0	9.60	ND
	8-iso-PGF_2α_	C20H34O5	34505	72.3	9.60	ND
	Lipoxin A4 (LXA4)	C20H32O5	6498	10.4	14.9	906
	Leukotriene B_4_ (LTB_4_)	C20H32O4	15647	1.56	1.78	105
	Leukotriene C_4_ (LTC_4_)	C30H47N3O9S	16978	3.68	59.4	10 898
	Leukotriene D_4_ (LTD_4_)	C25H40N2O6S	28666	0.26	2.85	175.71
	Leukotriene E_4_ (LTE_4_)	C23H37NO5S	15650	1.05	29.5	1842
	Prostaglandin E_2_ (PGE_2_)	C20H32O5	15551	3.87	31.0	ND
	Prostaglandin F_2α_ (PGF_2α_)	C20H34O5	15553	0.010	0.010	2.54
	Resolvin D1 (R_V_D1)	C22H32O5	81564	3.08	6.91	2193
	Resolvin D2 (R_V_D2)	C22H32O5	81565	ND	898.5	272 983

Saliva, clean-EBC, and saliva-EBC were collected from 13healthy volunteers (Cohort 1) as described in methods. Samples were pooled and 5 μL of each sample was injected onto an analytical column. Amino acids and eicosanoids were spiked into a control water sample and underwent the EBC sample collection procedure as shown in Fig. 2a. These authentic amino acid and eicosanoid standards were used to confirm compound identities in the EBC and saliva samples using exact mass, isotope ratios and retention time matching. Isomers could not be separated or differentiated using the LC-MS method described, and are listed together.^ND^indicates not detected. ng/mL indicates the calculated concentration of amino acids and eicosanoids in each of the pooled samples. Compound identifiers are ChEBI except for 5 eicosanoids with CAS identifiers. ^a^indicates undetected in all three sample groups

Forty compounds were of higher concentration in saliva compared to clean-EBC or saliva-EBC. In addition, twenty-five compounds were present at higher concentrations in the saliva-EBC samples compared to the clean-EBC. These included the following fifteen amino acids: 1-methylhistidine/3-methylhistidine, arginine, cystathionine, ethanolamine, glutamine, L-aminoadipic acid, L-cystine, leucine/isoleucine, ornithine, phenylalanine, phosphoserine, proline, sarcosine, taurine, urea, and valine. These also include eight eicosanoids: protectin DX, 17(S)-HDHA, LTC_4_, LTD_4_, LTE_4_, PGE_2_, RvD1, and RvD2. These compounds are most likely elevated due to salivary contamination of the EBC samples.

Ten compounds were detected at similar concentrations in both the clean-EBC and the saliva-EBC samples. These were alanine, aspartic acid, citrulline, creatinine, γ-aminobutyric acid, glycine, hydroxylysine, LXA4, LTB_4_, and PGF_2α_. Four amino acids (α-aminobutyric acid, glutamic acid, threonine, tryptophan) and five eicosanoids (11β-PGF_2α_, 14(S)-HDHA, 15R-PGF_2α_, 8-iso-15R-PGF_2α_, 8-iso-PGF_2α_) had higher concentrations in the clean-EBC compared to the saliva-EBC samples. Four compounds (asparagine, taurine, tyrosine, RvD2) were undetected in EBC compared to saliva. Alanine was lower in concentration in saliva compared to clean-EBC and saliva-EBC while an additional six compounds (cystathionine, 11β-PGF_2α_, 15R-PGF_2α_, 8-iso-15R-PGF_2α_, 8-iso-PGF_2α_, and PGE_2_) were undetected in the saliva samples but were detected in the clean-EBC and saliva-EBC.

### Detection of metabolites in healthy EBC using untargeted metabolomics

**Fig. 3:**
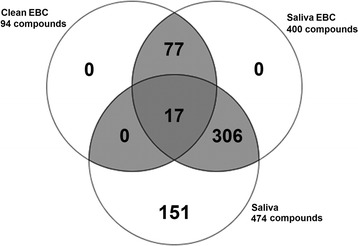
Overlap of metabolites detected in clean EBC, saliva-contaminated EBC, and saliva samples of healthy volunteers in Cohort 1. Untargeted metabolomics was performed on EBC and saliva from healthy volunteers. Metabolite peaks were extracted using MassHunter Profinder software (Agilent). Samples were filtered using a 3000 abundance cutoff and a presence in at least two of the three sample groups. A total of 77 metabolites were determined to be unique to EBC

LCMS-based metabolomics was used to determine what metabolites could be detected and identified in Cohort 1 (clean EBC, saliva-EBC, and saliva) using an untargeted approach. The overlap in metabolites from the LC-MS analysis of these samples is shown in Fig. 3. Overall, there were 400 metabolites detected in all samples; 306 were present in both saliva and saliva-EBC; 77 metabolites were detected in the clean-EBC and the saliva-EBC but were undetected in the saliva samples. From the 77 metabolites detected in the EBC and saliva-EBC samples, 40 metabolites were annotated using freely available small molecule databases (Table 2); 37 were either unannotated or annotated using molecular formula (Additional file 4). Tandem MS provided additional confidence in the identification of acetylsalicylic acid, 4-chloro-L-phenylalanine, 3,4-furandicarboxylic acid, shikimic acid, succinic acid, and citric acid (Additional file 5).

**Table 2:** Database annotated metabolites detected in healthy EBC

Compound Database Annotation	Formula	Mode	RT (min)	Mass	m/z	Adduct	ppm error	Identifier
C25-Allenic-apo-aldehyde	C25 H34 O3	+	8.24	382.2508	383.2579	[M + H]+	0.52	KEGG: C14044
								LMPR01070293
19α-19-Hydroxy-3,11-dioxo-12-ursen-28-oic	C29 H42 O5	+	8.40	470.3055	471.3124	[M + H]+	3.35	HMDB38683
2,2,4,4,-Tetramethyl-6-(1-oxopropyl)-1,3,5-cyclohexanetrione	C13 H18 O4	+	10.55	238.1205	261.1105	[M + Na]+	5.21	HMDB33191
Planinin	C21 H22 O6	-	13.10	370.1416	369.1348	[M-H]-	1.88	HMDB38236
25-Hydroxyvitamin D2-25-glucuronide	C34 H52 O8	+	8.34	588.3685	606.4037	[M + NH4]+	3.81	KEGG: C03033
								HMDB10342
3-Deoxy-3-azido-25-hydroxyvitamin D3	C27 H43 N3 O	+	7.69	425.3406	446.2932	[M + K-H2O]+	2.64	LMST03020677
3-keto Fusidic acid	C31 H46 O7	+	8.03	530.3267	548.3616	[M + NH4]+	5.04	HMDB60745
Mumefural	C12 H12 O9	-	8.36	300.0481	299.0388	[M-H]-	6.58	HMDB35179
3-Oxooctanoic acid	C8 H14 O3	+	8.09	158.0940	159.1007	[M + H]+		HMDB10721
Fluometuron	C10 H11 F3 N2 O	-	9.40	232.0823	463.1564	[2 M-H]-	1.49	KEGG: C18853
Dibenzyl ether	C14 H14 O	-	13.73	198.1039	197.0967	[M-H]-	2.85	HMDB32078
Marmesin rutinoside	C26 H34 O13	+	10.16	554.1999	537.1973	[M + H-H2O]+	1.12	HMDB41413
Amitraz	C19 H23 N3	+	7.79	293.1892	316.1790	[M + Na]+	4.13	KEGG: C10995
								CAS: 33089-61-1
Glutamyl-Glycine	C10 H7 N3 O	+	11.72	223.0123	224.0203	[M + K-H2O]+	3.63	HMDB28819
de-Hypoxanthine futalosine	C14 H16 O7	+	1.77	296.0896	149.0523	[M + 2H]2+	0.78	KEGG: C17010
Diethyltoluamide (DEET)	C12 H17 N O	-	16.74	251.1519	250.1451	[M + CH3COO]-	0.05	KEGG: C10935
								CAS: 134-62-3
3,4-Dihydroxyfluorene	C13 H10 O2	+	11.38	198.0691	181.0661	[M + H-H2O]+	6.66	KEGG: C07717 CAS: 42523-20-6
2,3-Dihydro-2,3-dihydroxy-4-(4-methoxyphenyl)-1H-phenalen-1-one	C20 H16 O4	-	7.63	320.1049	319.0981	[M-H]-	1.49	HMDB41463
3-Oxopregn-4-ene-20beta-carboxaldehyde dioxime	C22 H34 N2 O2	+	8.66	358.2620	422.2817	[M + ACN + Na]+	9.24	KEGG: C15106
Hyperin 2''-[glucosyl-(1- > 3)-rhamnoside] 6''-rhamnoside	C39 H50 O25	-	12.42	918.2638	917.2567	[M-H]-	0.20	HMDB39911
Mangostanol	C24 H26 O7	-	13.02	426.1679	485.1805	[M + CH3COO]-	3.73	HMDB29868
Oleoside dimethyl ester	C18 H26 O11	-	9.79	418.1475	453.1160	[M + Cl]-	1.23	HMDB31350
N-Acetyl-6-O-L-fucosyl-D-glucosamine	C14 H25 N O10	-	8.49	349.1370	348.1292	[M-H2O-H]-	3.03	HMDB02220
								CAS: 109582-58-3
Oleanolic acid 3-O-beta-D-glucosiduronic acid	C36 H56 O9	+	8.40	632.3924	650.4312	[M + NH4]+	6.2	KEGG: C08964
Methionyl-Arginine	C11 H23 N5 O3 S	+	11.97	305.1522	288.1497	[M + H-H2O]+	2.01	HMDB28967
N-Cyclopropylammelide	C6 H8 N4 O2	-	9.27	204.0403	203.0329	[M-Cl]-	8.76	KEGG: C14149
PE(34:1)-15-isoLG hydroxylactam	C59 H104 N O13 P	+	10.01	1065.7160	1088.7032	[M + Na]+	7.06	KEGG: C06254
								LMGP00000061
PE(44:7)	C49 H84 N O8 P	+	9.75	845.5867	868.5782	[M + Na]+	5.18	KEGG: C00350
								HMDB09700
Prostaglandin F_2α_-biotin	C35 H60 N4 O6 S	+	8.71	664.4234	669.3982	[M + Na-H2O]+	7.77	
Prostaglandin D2-biotin	C36 H60 N4 O6 S	+	8.47	676.4234	694.4563	[M + NH4]+	1.30	
Prostaglandin E2-biotin	C35 H58 N4 O6 S	+	8.24	662.4045	663.4127	[M + NH4]+	1.63	
Tyrosol-histidine	C15 H18 N4 O4	+	7.90	300.1224	301.1300	[M + H-H2O]+	2.79	HMDB29107
S-Farnesyl Thioacetic Acid	C17 H28 O2 S	+	7.44	296.1797	297.1891	[M + H]+	0.87	CAS: 135784-48-4
Terbucarb	C17 H27 N O2	+	13.58	277.2047	278.2088	[M + H]+	9.9	KEGG: C19129
								CAS: 1918-11-2
8-Hydroxypinoresinol 4-glucoside	C26 H32 O12	+	9.95	536.1894	537.1986	[M + H]+	6.55	KEGG: C07149
								HMDB14643
								CAS: 26171-23-3
Ganglioside GM_3_ (d18:0/20:0)	C61 H114 N2 O21	+	10.09	1210.7914	597.3970	(M + 2H) + 2[-H2O]+		KEGG: C04730
								HMDB11919
Beta-Santalic acid	C15 H22 O2	-	15.63	234.1620	233.1548	[M-H]-	2.93	HMDB39621
Phenylalanyl-Histidine	C15 H18 N4 O3	+	7.77	302.1379	285.1354	[M + H-H2O]+	2.81	HMDB28997
4-Hydroxyphenylacetaldehyde	C8 H8 O2	-	9.28	136.0524	135.0450	[M-H]-	3.11	KEGG: C03765
								HMDB03767
Pimelylcarnitine	C14 H25 N O6	-	8.98	303.1682	284.1487	[M-H2O-H]-	3.87	CAS: 7339-87-9

Database annotations were obtained for 40 of the 77 compounds that were specific to the EBC samples (Fig. 3). Samples were analyzed in positive and negative ionization mode using LC-MS untargeted metabolomics on an SB-AQ analytical column. Annotations were based on an in-house database comprising KEGG, HMDB, Lipid Maps, and Metlin. + indicates detected in positive ionization mode, - indicates detected in negative ionization mode

### Analysis of eicosanoids in saliva and EBC of asthmatics

**Fig. 4:**
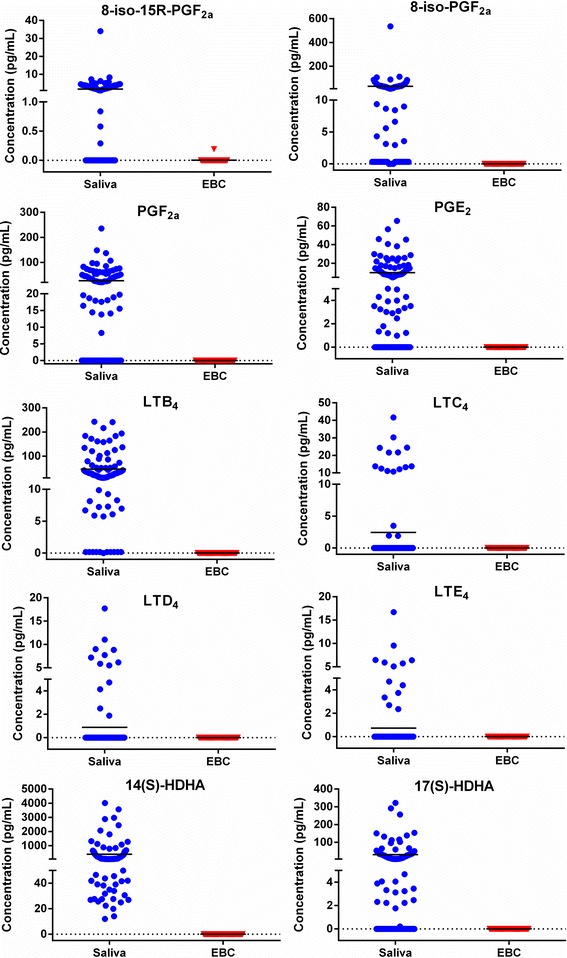
Concentrations of ten eicosanoids in saliva and EBC samples of matched asthmatics subjects in Cohort 2. Quantitative analysis was performed on an Agilent triple quadrupole (QQQ) 6410 mass spectrometer using targeted multiple reaction monitoring (MRM); concentration units in pg/mL; blue circles are saliva samples (*n* = 106), red triangles are EBC samples (*n* = 107); black line is sample mean

Since EBC has potential as a clinical diagnostic in lung diseases, we sought to determine if previously reported eicosanoid molecules could be detected in clean EBC or if their detection was the result of saliva contamination. Matched saliva and EBC was collected from 107 asthmatic subjects (Cohort 2) for this purpose (Fig. 4). Eicosanoid analysis was performed using multiple reaction monitoring (MRM) on a triple quadrupole mass spectrometer (QQQ-MS). Sixteen eicosanoids comprised the panel as detailed in the methods. Only one molecule, 8-iso-15R-PGF_2α_, was detected in EBC and in only one subject. Conversely, ten out of the sixteen eicosanoids were detected in a significant number of saliva samples including 8-iso-15R-PGF_2α_ (*n* = 47), 8-iso-PGF_2α_ (*n* = 102), PGF_2α_ (*n* = 56), PGE_2_ (*n* = 77), LTB_4_ (*n* = 105), LTC_4_ (*n* = 16), LTD_4_ (*n* = 13), LTE_4_ (*n* = 13), 17(S)-HDHA (*n* = 79), and 14(S)-HDHA (*n* = 106). One eicosanoid, 14(S)-HDHA, was detected in all 106 saliva samples; this may be due to its higher concentration levels (12 pg/mL – 4014 pg/mL) compared to the other eicosanoids (0.15 pg/mL – 535 pg/mL).

In order to determine if eicosanoids were present in EBC but below the limit of detection, remaining EBC from the asthmatics subjects (*n* = 107) was pooled into a single 13 mL aliquot and spiked with five deuterated eicosanoid standards (PGE_2_-d4, LTB_4_-d4, LTC_4_-d5, LTD_4_-d5, LTE_4_-d5). The sample was lyophilized, reconstituted in 20 μL of HPLC buffer, and the entire amount analyzed via LC-MS in full scan positive ionization mode. After subtracting solvent blanks, 97 metabolites were detected in EBC, including tentative identifications for LTE_3_, thromboxane, 11-trans-LTE_4_, 11-trans-LTC_4_, and 12-oxo-LTB_4_ (Additional file 6). While it is possible that these molecules were the result of degradation of the standards, this result also suggests that eicosanoids may be present in EBC, albeit at very low concentrations.

### Replication experiment using targeted LC-MS, untargeted LC-MS, and proteomics to examine healthy, sick, and smoker EBC

**Fig. 5:**
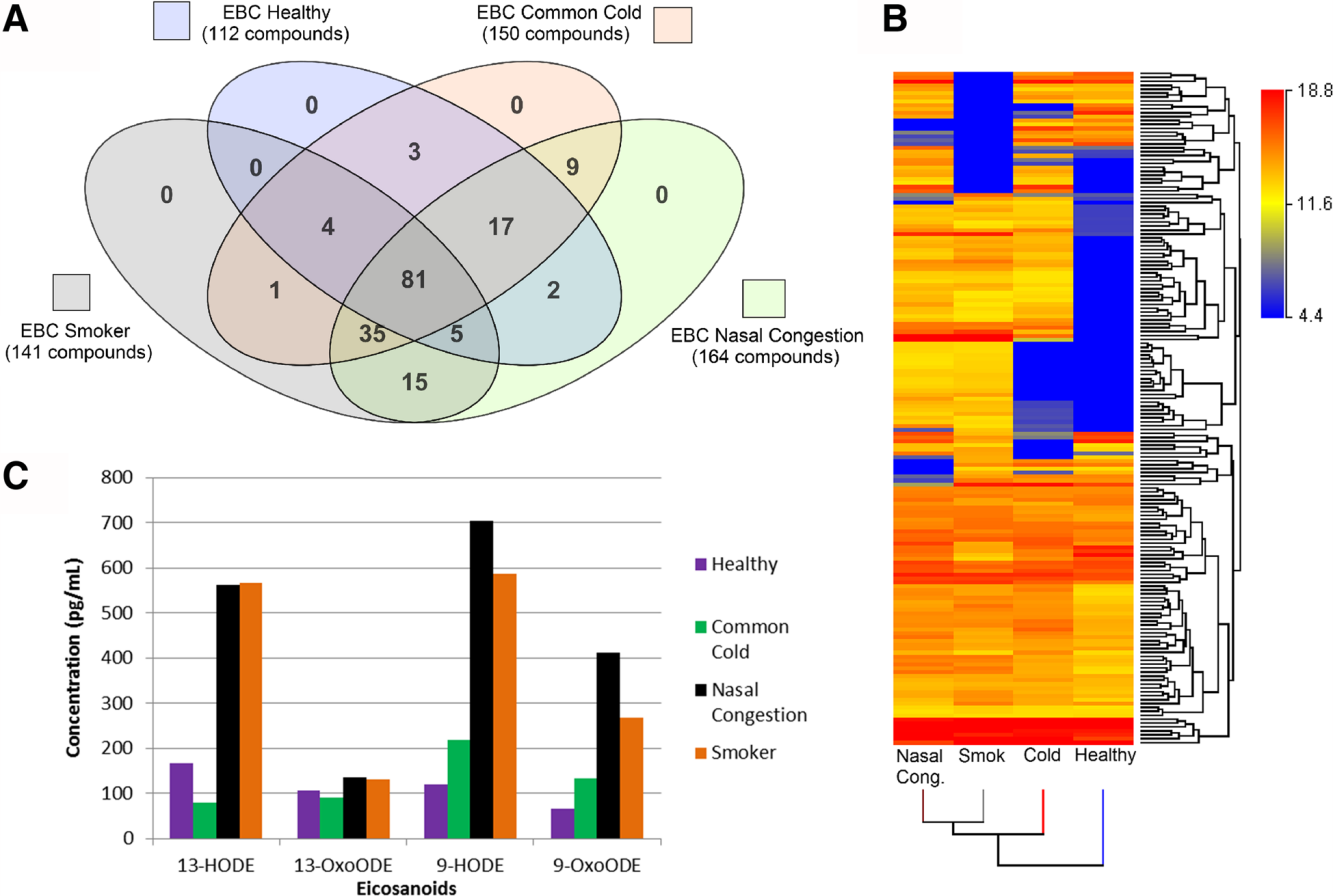
Distribution of compounds across EBC groups in Cohort 3. **a** Venn diagram depicting the overlap of metabolites in four categories based on subject from the untargeted metabolomics analysis. Metabolites were filtered for presence in at least two out of the four groups; **b** Hierarchical clustering of 172 metabolites present in at least two EBC groups. Blue sections indicate low metabolite abundances and red sections indicate high abundance levels. The healthy EBC subjects appear to have a majority of lower abundance metabolites compared to the other three groups; **c** Concentration levels of four eicosanoids detected in all four sample groups using targeted LC-MS. Samples were analyzed on a triple quadruple mass spectrometer

To confirm that small molecules are detectable in EBC and to further rule out the possibility of saliva contamination, a replication experiment was performed with EBC collected from 13 subjects (Cohort 3). Samples were classified into four groups based on health status: healthy non-smoker, healthy smoker, non-smoker with the common cold, non-smoker with nasal congestion. A saliva trap was used and subjects were observed to ensure that each participant did not contaminate the samples with saliva during the EBC collection procedure. Overall, a total of 172 metabolites were detected when untargeted LC-MS metabolomics was used (Fig. 5a); of these 118 were database annotated and 81 metabolites were common to all four groups. While the sample numbers are too low to enable the use of statistics, it is useful to discuss the results in the context of the individual groups. The healthy group contained the least number of metabolites (112) compared to the smoker (141), common cold (150), and nasal congestion (164) groups (Fig. 5a, b). Amino acids and their derivatives were unique to the smoker EBC (Table 3). A complete list of annotated and MS/MS hits is available in Additional file 7.

**Table 3:** Compounds detected in healthy, sick, and smoker EBC

Compound	Smoker	Nasal	Cold	Healthy	RT (mins)	Mass	Formula	Mode	Identifier
1,3-Dicyclohexylurea^a^	✓	✓	✓	✓	8.876	224.1889	C13 H24 N2 O	+	CAS: 2387-23-7
13,14-dihydro Prostaglandin F1a	✓	✓	✓	✓	6.814	358.2736	C20 H38 O5	+	CAS: 20592-20-5
1-Hydroxy-2-naphthoic acid	✓	✓	✓	✓	6.258	188.0472	C11 H8 O3	+	KEGG: C03203
2-Amino-3,7-dideoxy-D-threo-hept-6-ulosonic acid	✓	✓	✓	✓	6.380	213.0620	C7 H13 N O5	+	KEGG: C16850
C14 sphingosine	✓	✓	✓	✓	9.628	271.2141	C14 H29 N O2	-	LMSP01040006
N-Acetyl-D-fucosamine	✓	✓	✓	✓	8.079	205.0950	C8 H15 N O5	+	KEGG: C15480
p-Cymene	✓	✓	✓	✓	9.382	152.1205	C10 H14	+	KEGG: C06575
Ureidoglycine	✓	✓	✓	✓	0.980	380.0903	C3 H7 N3 O3	-	KEGG: C02091
15(S)-HPETE	✓	✓		✓	10.185	264.2089	C20 H32 O4	+	KEGG: C05966
6-hydroxy caproic acid	✓	✓	✓		6.131	174.0890	C6 H12 O3	-	KEGG: C06103
Homoserine	✓	✓	✓		6.707	215.0406	C4 H9 N O3	-	KEGG: C00263
PA(22:2/0:0)	✓	✓	✓		6.118	490.3105	C25 H47 O7 P	-	KEGG: C00416
PI(12:0/12:0)	✓	✓	✓		5.703	698.3983	C33 H63 O13 P	-	KEGG: C01194
Tetrahydrodipicolinate	✓	✓	✓		7.780	153.0427	C7 H9 N O4	-	KEGG: C03972
Threonine	✓	✓	✓		8.939	233.0513	C4 H9 N O3	-	KEGG: C00188
2-Oxo-4-hydroxy-5-aminovalerate	✓	✓			1.604	129.0426	C5 H9 N O4	-	KEGG: C05941
N-Acetyl leucine	✓	✓			5.710	173.1055	C8 H15 N O3	-	KEGG: C02710
2-Amino-m-cresol^a^	✓				0.811	123.0684	C7 H9 N O	+	CAS: 2835-97-4
3-Cyano-6-methoxycoumarin^a^	✓				5.936	201.0426	C11 H7 N O3	+	-
3-Methylhistidine^a^	✓				0.809	169.0851	C7 H11 N3 O2	+	KEGG: C01152
4-Imidazoleacrylic acid^a^	✓				5.023	138.0429	C6 H6 N2 O2	+	HMDB00301
5-Aminosalicylic acid^a^	✓				1.612	153.0426	C7 H7 N O3	+	KEGG: C07138
Acetyl arginine^a^	✓				4.910	216.1222	C8 H16 N4 O3	+	HMDB04620
Arginine^a^	✓				0.810	174.1117	C6 H14 N4 O2	+	KEGG: C02385
Carnosine^a^	✓				0.809	226.1066	C9 H14 N4 O3	+	KEGG: C00386
D-erythro-Sphinganine^a^	✓				5.186	301.2981	C18 H39 N O2	+	LMSP01020001
Triethyl citrate^a^	✓				7.519	276.1209	C12 H20 O7	+	CAS: 77-93-0
Tryptophan^a^	✓				4.079	204.0899	C11 H12 N2 O2	+	KEGG: C00078
1alpha,24,25,28-tetrahydroxyvitamin D2		✓	✓		6.213	460.3189	C28 H44 O5	-	LMST03010055
MG(18:1)		✓	✓	✓	8.908	373.3185	C21 H40 O4	+	KEGG: C01885
PC(18:1/22:6)		✓	✓	✓	6.813	831.5785	C48 H82 N O8 P	+	KEGG: C00157
PG(18:4/20:4)		✓	✓	✓	12.183	790.4734	C44 H71 O10 P	+	KEGG: C00344
O-decanoyl-R-carnitine		✓	✓		6.305	361.2453	C17 H33 N O4	-	KEGG: C03299
Palmitoylglycine		✓		✓	9.931	335.2469	C18 H35 N O3	+	HMDB13034
Arogenate			✓	✓	6.828	227.0775	C10 H13 N O5	-	KEGG: C00826
α-Lipoic acid^a^			✓		5.666	206.0435	C8 H14 O2 S2	+	KEGG: C00725
Ephedrine^a^			✓		31.317	165.1154	C10 H15 N O	+	KEGG: C01575
3-Acetyl-8-methoxycoumarin^a^			✓		5.846	218.0579	C12 H10 O4	+	HMDB34345
6-Hydroxymelatonin^a^				✓	1.184	248.1161	C13 H16 N2 O3	+	KEGG: C05643

EBC was collected from 13 volunteers and pooled into four groups; healthy smokers, healthy non-smokers, non-smokers with nasal congestion, and non-smokers with the common cold. ✓ indicates that a compound was detected. ^a^ indicates tandem MS fragmentation patterns were matched to the NIST14 Mass Spectral library using the NIST MS Search v.2.2 g program. MF: match factor; RMF: reverse match score. The fragmentation spectra for the listed compounds are available in the Additional file 7

Targeted analysis of 32 eicosanoids resulted in the detection of 19 compounds in the smoker group; these were undetected or below our limit of quantitation in the other three EBC groups (Additional file 8). Seven of those eicosanoids were part of the cyclooxygenase pathway, 9 were part of the 5-, 12-, or 15-lipoxygenase pathways, and 2 were in the lipid peroxidation/oxidative stress pathway. Four eicosanoids were detected and quantified in all four groups (Fig. 5c). The smoker and the nasal congestion group showed elevated levels of 13-HODE, 13-OxoODE, 9-HODE, and 9-OxoODE compared to the healthy and common cold groups. A comprehensive pathway analysis was performed across all sample groups (Additional File 9) which showed that more pathways were detected in the smoker EBC compared to the other groups in that cohort. Pathway analysis also included Cohorts 1 and 2, of which the saliva-based samples showed a greater number of pathways compared to the EBC-only samples. These results were not unexpected as the saliva samples contained a larger number of compounds than the EBC samples.

Three proteins were detected in EBC samples of Cohort 3 subjects following proteomics analysis (Table 4). Zinc finger protein 800 and myoneurin were only detected in the smoker EBC. Cytokeratin 9 was only detected in the healthy EBC. No proteins were detected in the nasal congestion or common cold EBC.

**Table 4:** Proteins detected in healthy non-smoker and healthy smoker EBC

Protein	Accession ID	Smoker	Nasal Congestion	Cold/Flu	Healthy
Zinc finger protein 800	UniProtKB Q2TB10	✓			
Myoneurin	UniProtKB Q9NPC7	✓			
Keratin, type 1 cytoskeleton 9 (Cytokeratin 9)	UniProtKB P35527				✓

EBC was collected from 13 volunteers and pooled into 4 groups; healthy smokers, healthy non-smokers, non-smokers with nasal congestion, and non-smokers with the common cold. Samples were analyzed on a Bruker Impact HD Q-TOF and proteins were search using Mascot. ✓ indicates that a protein was detected within a particular group

## Discussion

The purpose of this study was to determine the utility of EBC in studying lung diseases, especially asthma. Therefore, we aimed to (1) evaluate whether compound adsorption to collection tubes is the reason for low-to-no detection of metabolites in some EBC studies, (2) characterize the constituents of EBC using untargeted and targeted mass spectrometry, and (3) determine if the detection of molecules in EBC is due to saliva contamination during sample collection. Initial EBC experiments using the RTube for sample collection yielded no detectable levels of leukotrienes in our previous studies (data not shown). Other studies have also showed the RTube to be less sensitive than other commercially available EBC collection devices [36]. Since there is the possibility of binding of compounds to the sides of the collection tube, a control experiment was performed (Fig. 1). Results showed 10-22% less adsorption of the leukotrienes to the TURBO-DECCS plastic tube compared to the plastic RTube at the 10 pg/mL spike levels (Fig. 1d). In addition, polyethylene terephthalate (PET) caused significant adsorption of LTC_4_, LTD_4_, and LTE_4_ to the coated collection tubes (Fig. 1d). Therefore, our clinical experiments used the TURBO-DECCS plastic collection tube without PET to minimize adsorption of compounds to the plastic.

Our first goal was to identify the contribution of saliva to EBC measurements by determining concentrations of amino acids and eicosanoids in both EBC and saliva. We observed that the concentration of several eicosanoids and amino acids (Table 1) were orders of magnitude higher in saliva compared to clean-EBC and saliva-EBC. Although a saliva trap was used, it may be possible that saliva contributed to the ng/mL concentration values obtained in both the clean-EBC and saliva-EBC sample group. As suggested by Gaber et al [25], even the slightest amount of saliva can generate false positives in EBC samples when sensitive detection methods are used. Since LTB_4_ is present in the nasal mucosa, and the oropharyngeal tract contributes to the contents of EBC, a more sensitive or alternate method to alpha-amylase detection may be required to confirm saliva contamination. In our study, we detected small molecules in EBC and saliva-EBC which were not detected in the saliva-only samples. This suggests that these molecules may be specific to EBC or have much lower concentrations in saliva; therefore, the low saliva content would not contribute to their detection in EBC. These EBC constituents may potentially be used as biomarkers; however, further study is required to confirm their identities and usefulness in studying lung disease.

Previous investigations have reported the presence of eicosanoids in EBC [11, 15, 37, 38]. Many of these groups have reported higher concentrations of eicosanoids than those shown in Table 1 of the current study; the majority of these studies were conducted in the context of lung disease such as asthma where elevated levels may due to inflammation. The healthy volunteers in our study had no history of asthma or lung inflammation; this could explain the lower levels which we report. We compared our concentration values of amino acids and eicosanoids in Table 1 to those available in the literature for EBC and saliva. LTB_4_ has been reported to have an average concentration in saliva of 0.467 ng/mL [25] which is similar to our estimated value of 1.56 ng/mL. Tyrosine has been reported at an average of 33.3 ng/mL [24] in EBC while Conventz et al [19] reported an average value of 15.5 ng/mL. We detected tyrosine at 27.1 ng/mL in ‘saliva-EBC’ but it was undetected in ‘clean-EBC’. Our proline estimate was 111.4 ng/mL in EBC. This was twice as high as the previously reported high value in healthy subjects of 51.9 ng/mL [19] and may be the result of individual subject differences, sample preparation, or detection method. Reported EBC urea values have similar ranges within the same order of magnitude to our calculated values. Effros et al [39] reported values in the range 0.33 to 0.39 μmol/L (19.8 to 23.4 ng/mL). Folesani et al [40] reported EBC urea concentrations found in healthy controls in the range 0.7-1.3 μM (42.0 to 78.1 ng/mL). Dwyer et al [41] reported EBC urea averaged 0.52 +/- 0.12 μmol/L (31.2 ng/mL) in 18 individuals. Our detected levels ranged from 1.56 ng/mL (clean-EBC) to 65.1 ng/mL (saliva-EBC) which falls within the same order of magnitude of these previously reported values. The differences among studies could be accounted for by variations in sample preparation procedures such as lyophilization, as well as differences in methods of collecting EBC.

Our second goal was to characterize the constituents of EBC using an untargeted metabolomics approach. We further determined the effect of saliva contamination on small molecules detection in EBC. The 77 small molecules that were detected in the clean-EBC and the saliva-EBC were undetected in the saliva samples; this suggests that these compounds may be specific to EBC. The 40 out of 77 that were database annotated using Human Metabolome Database (HMDB) [42] and the Kyoto Encyclopedia and Genes and Genomes (KEGG) [43] included the following: vitamin D metabolites, lipids, herbs, spices, food, plants, insecticides, herbicides, dipeptides, and PAH degradants. Five out of fourteen food metabolites were related to citrus fruit or tea, three were herbs and spices, and the remaining were vegetables, food flavorings, or oils. This is consistent with participants’ reports of drinking green tea, coffee, and chocolate milk, and eating pumpkin cake, green beans, club sandwich, and potato chips. Note that participants were not allowed to eat or drink at least two hours prior to sample collection. Other matches for two insecticides and three herbicides were also plausible since these samples were collected when subjects may have applied insect repellants such as diethyltoluamide (DEET) to their skin, and institutions were applying herbicides and pesticides to their lawns. For example, N-cyclopropylammelide is a degradation product of atrazine. Atrazine is an herbicide used to prevent weeds on golf courses and residential lawns.

Other metabolites found in EBC were tentatively identified as endogenous compounds; for example, 3-oxooctanoic acid is an endogenous keto acid involved in fatty acid biosynthesis. It is formed by the action of acid synthases from acetyl-CoA and malonyl-CoA precursors. PE(44:7) is an endogenous glycerophospholipid associated with cell signaling and membrane integrity and also serves as an energy source [42]. Ganglioside GM_3_ (d18:0/20:0) is an endogenous sphingolipid. Gangliosides, including GM_3_ and GM_2_, have been shown to be down-regulated in the hyper-reactive lung and trachea compared to the normal lung and trachea in a guinea pig model of bronchial asthma [44]. Gangliosides have also been shown to be inversely associated with severe emphysema in COPD human plasma [45]. Dipeptides were also annotated. These included glutamyl-glycine, methionyl-arginine, tyrosyl-histidine, and phenylalanyl-histidine. Dipeptides are incomplete breakdown products of protein digestion or protein catabolism. Many dipeptides are short-lived intermediates toward specific amino acid degradation pathways while others have physiological [46] or cell-signaling effects [42].

3,4-dihydroxyfluorene is a polycyclic aromatic hydrocarbon (PAH) degradation metabolite. PAH’s have been associated with childhood asthma [47] and are found in oil, coal, and tar. They are the result of combustion in engines, and incinerators; sources include forest fires, vehicle exhaust, grilling or barbecuing meat, and smoked fish [48–50]. Lastly, there were two annotated vitamin D metabolites (25-hydroxyvitamin D_2_-25-glucuronide and 3-deoxy-3-azido-25-hydroxyvitamin D_3_). Vitamin D and its metabolites are associated with asthma [51, 52] and vitamin D deficiency has been shown to be a risk factor for developing asthma [53, 54]. Because these compounds have previously reported associations with lung disease, they could either be used as diagnostic markers of health or disease state, or may be novel molecules requiring further interrogation.

Due to low sample volumes, tandem mass spectrometry was only performed in negative mode and resulted in six spectral library matches corresponding to acetylsalicylic acid, 4-chloro-L-phenylalanine, 3,4-furandicarboxylic acid, shikimic acid, succinic acid, and citric acid (Additional file 5). With additional sample, targeted MSMS can be performed on additional EBC metabolites to further explore their identities. Other metabolites present may be below our limit of detection or require more specialized sample preparation techniques. Therefore, detection of specific molecules or classes, may require derivatization, solid phase extraction, or enzymatic techniques, as described by Chérot-Kornobis et al [17] for nitrogen oxides, Esther Jr. et al [55] for purines, or Rossi et al [56] for glutathione.

A major objective of the current study was to determine if eicosanoid detection in EBC was the result of saliva contamination. With the exception of one molecule in one subject, we were unable to detect eicosanoids in EBC of over 100 asthmatic subjects, a group in which higher concentrations of these molecules have been reported (Fig. 4). Our EBC sample preparation step included diluting the EBC volume to 1 mL; this may have diluted levels to below the limit of detection for the targeted eicosanoid panel. However, the concentration of eicosanoids ranged from 10-fold to 100-fold lower in EBC compared to saliva from matched asthmatic subjects (Fig. 4). Since our sample preparation methods, including low starting volumes, were consistent with previous investigations reporting the detection of eicosanoids in EBC, this suggests that previously reported values could possibly be due to salivary contamination. This is further supported by the detection of some eicosanoids when a very highly concentrated (13 ml) EBC sample is used. Within the asthmatic saliva samples, the concentrations varied in the range 58-187% CV for the samples with detectable levels of eicosanoids. These results show that eicosanoid concentrations in saliva vary widely amongst asthmatic subjects.

Due to the poor detection of eicosanoids in EBC using targeted analysis and low starting volumes, we investigated whether any other small molecules could be detected in EBC when larger starting volumes are used. Leftover EBC from asthmatic subjects (*n* = 107) was pooled into a 13 mL aliquot, lyophilized, and reconstituted in 20 μL of HPLC buffer. Untargeted LC-MS revealed 97 metabolites, of which four were eicosanoid derivatives: LTE_3_, thromboxane, 11-trans-LTE_4_, 11-trans-LTC_4_, and 12-oxo-LTB_4_ (Additional file 6). Results suggest that although eicosanoid metabolites are present in EBC, samples require a drastic pre-concentration step prior to LC-MS analysis in order for these molecules to be detected. In addition, some of these detected metabolites may be breakdown products of the deuterated standards spiked during sample preparation, particularly during the lyophilization step.

In a few studies, investigators increased the amount of EBC used to 1 mL and obtained detectable metabolite signal. For example, Pelclová et al [11] collected EBC over 15-20 min using the ECoScreen. They analyzed EBC of 82 patients with occupational lung diseases & 27 controls were analyzed using SPE followed by LC-ESI-MS/MS. Results showed elevation of LTB_4_, LTC_4_, and LTE_4_ in asbestos-exposed and silica-exposed patients compared to controls. Conventz et al [19] collected 1-6 mL EBC from 27 healthy adults also using the ECoScreen. 1 mL EBC was used to quantify proline, hydroxyproline, and tyrosine using LC-ESI-MS/MS. Fritscher et al [6] collected at least 1 mL of EBC from 87 subjects for over 10 min and performed targeted analysis on a QQQ-MS. They examined twenty-three eicosanoids in the EBC from asthma and COPD individuals, five of which overlapped with our study. Our study examined an additional eleven molecules that were not present in their study.

A major difference between our study and others is that our aim was specifically to determine the potential for and extent of saliva contamination in EBC sampling. Therefore, our studies incorporated a well-controlled set of experiments that included mimicking EBC collection. Overall, we required greater than 1 mL EBC for detection of eicosanoids; preparation included concentrating samples in a lyophilizer. Other investigators have also concentrated their EBC samples prior to analysis. Montuschi et al [57] collected 1.5 mL EBC per subject over 15 min using the ECoScreen and concentrated the EBC 40-fold. 20 μL of sample was injected and analyzed for LTB_4_ using LC-MS & LC-MS/MS.

Our methods were replicated in an independent cohort. EBC was collected from 13 volunteers and grouped in four categories based on health and smoking status: healthy non-smoker, healthy smoker, non-smoker with common cold, and non-smoker with nasal congestion. Saliva contamination was not present, as measured by a proteomic approach. Using untargeted LC-MS metabolomics, 172 metabolites were detected in EBC, 81 of which were present in all 4 sample groups (Fig. 5a). Fold changes were observed (Fig. 5b) but no statistical inferences could be made. Qualitatively however, the healthy EBC contained fewer compounds compared to the EBC of subjects with nasal congestion, the common cold, or the smoker (Fig. 5a). This may be due to differences in diet, or because subjects with signs of illness may ingest cold, flu, or nasal decongestion medication which may artificially increase the number of detected compounds in their EBC.

Some of the detected metabolites in the smoker, healthy, common cold, and nasal congestion EBC (Table 3 and Additional file 7) were markers of environmental exposure such as 1-hydroxy-2-naphthoic acid which is a PAH and naphthalene degradation metabolite. This metabolite also mapped to the “degradation of aromatic compounds” pathway, as did 6-hydroxy caproic acid and p-cymene. Ephedrine, used as a decongestant, was only detected in the common cold group. Additional detected metabolites such as 2-Oxo-4-hydroxy-5-aminovalerate, homoserine, arogenate, tetrahydrodipicolinate, and 2-amino-3,7-dideoxy-D-threo-hept-6-ulosonic acid play roles in amino acid metabolism and/or biosynthesis [58]. Many of these compounds were absent in the healthy EBC but were present in the EBC of individuals who smoked, had nasal congestion, or had the common cold. Other detected metabolites were part of purine metabolism [ureidoglycine], sphingolipid signaling pathway [C14 sphingosine], glycerolipid metabolism [PA(22:2), MG(18:1)], and glycerophospholipid metabolism [PC(40:7), PG(38:8), PA(22:2), PI(24:0)]. The lipid compounds PA(22:2) and PI(24:0) were undetected in the healthy EBC but were detected in the other three groups. These biological pathways have been implicated in lung diseases such as asthma [59] and COPD [45, 60].

Targeted eicosanoid analysis was also performed on the healthy, smoker, common cold, and nasal congestion EBC. Nineteen eicosanoids were detected in the smoker EBC which were undetected in the other groups. Elevated levels of 13-HODE, 13-OxoODE, 9-HODE, and 9-OxoODE were observed in both the smoker and nasal congestion EBC compared to the healthy and common cold EBC. This increase in eicosanoids in smoking samples has been observed in previous studies. Sanak et al [61] analyzed EBC from 17 healthy smokers and 41 healthy non-smokers collected using the ECoScreen. Results showed an increase in 5-HETE and 8-iso-PGF2α in the current smokers compared to the non-smokers. We conclude that cigarette smoking increases the inflammatory and oxidative stress markers observed in our study to levels that were at least 3-fold higher compared to the healthy EBC.

Proteomics analysis of these four groups detected few proteins in the EBC samples using both human and bacterial searches (Table 4). In a review in 2014, Harshman et al. [18] summarized 80 detected proteins in EBC from the current literature including one detected in our study. Although no proteins were detected in our nasal congestion or common cold EBC samples, keratin type I cytoskeleton 9 (Cytokeratin-9) was detected in healthy EBC. Cytokeratin-9 has been previously been identified in asthmatic EBC [62], and in the pooled EBC of non-smokers and healthy smokers [63]. However, others have suggested that cytokeratin in EBC is the result of ambient air rather than the airways [64]. We detected zinc finger protein 800 and myoneurin in the smoker EBC. Zinc finger protein 800 and myoneurin (also a zinc finger protein) have not been previously reported in EBC. Other zinc finger proteins have been detected; zinc finger CCCH domain-containing protein 4 (ZC3H4) has been reported in healthy non-smoker and healthy smoker EBC [63]. In our study, no salivary proteins were detected, indicating that the EBC samples from the replication study were not contaminated with saliva. A larger, more diverse cohort encompassing multiple lung diseases may be required to explore the diversity of exhaled proteins.

This study is particularly significant to EBC researchers because it emphasizes the issues of compound adsorption, saliva contamination, and high volumes of EBC required for biomarker discovery studies. The strengths of this study lie in the precise methods used and the large sample size of Cohort 2 in the asthmatic study. We recognize that some limitations exist. First, the sample number is limited for Cohorts 1 and 3. Second, subjects in these cohorts only refrained from food intake for 2-3 h rather than 12 h, which could explain some differences in metabolites detected. Lastly, due to limited sample volumes, additional analyses could not be performed to compare the three cohorts across all mass spectrometry technologies. Future studies could be aimed at rectifying these limitations.

## Conclusions

We conclude that measureable levels of small molecules, including amino acids and eicosanoids, are present in healthy EBC; however, the dilute nature of EBC requires larger volumes of starting material than currently reported in the literature. We suggest the collection of at least 15 mL of EBC per subject and pre-concentrating by at least 20-fold to as much as 500-fold prior to LC-MS analysis in order to confidently and reproducibly detect metabolites of interest. Secondly, although α-amylase assays can test for the presence of saliva in EBC, small volumes of saliva may still be present but be below the detection limits of the assay. Since saliva can be responsible for contaminating EBC samples, proper sample collection and handling is necessary, particularly the use of a saliva trap during sample collection. Thirdly, eicosanoid concentrations in saliva vary widely amongst asthmatic subjects and this should be considered when designing experiments. Here, we provide a general presentation of EBC constituents from which investigators can probe more specialized techniques to detect additional or lower abundant compounds of interest. These results suggest that large volumes of samples and a more targeted approach are needed when using EBC to study asthma and other lung diseases.

## Additional files

Additional file 1: Targeted mass spectrometry parameters for eicosanoid analysis. MRM parameters, retention times, associated internal standards, and ionization modes used for lipid mediators by LC-MS/MS. IS: internal standard. (PDF 23 kb)

Additional file 2: Peak areas of selected amino acids and eicosanoids detected in EBC and/or saliva samples. (A) Peak areas of selected eicosanoids using untargeted metabolomics; (B) Peak areas of selected amino acids in spiked water (green), clean-EBC (black), saliva-EBC (blue), and saliva (red) using untargeted metabolomics. A control water sample which was spiked with known concentrations of amino acids and eicosanoids was used to confirm the identities of these compounds in the saliva and EBC samples using exact mass, isotope ratios and retention time. (C) Peak area of LTB_4_ in internal standard, EBC and saliva using targeted analysis. (D) Peak area of LTE_4_ in internal standard and EBC using targeted analysis. Peak areas were extracted using MassHunter Quantitative Analysis software (Agilent). y-axis: mass spectral counts; x-axis: retention time. Starting volumes for untargeted metabolomics was 11.5 mL (clean-EBC) and 7.5 mL (saliva-EBC) with final volume of 20 μL and injection volume of 5 μL. Starting volume for targeted analysis was 1 mL saliva or EBC with an injection volume of 100 μL. (TIF 1669 kb)

Additional file 3: Separation of PGF_2α_ isomers in spiked control water. Samples were injected onto an SB-AQ analytical column. Since the four isomers could not be differentiated using untargeted analysis, multiple reaction monitoring (MRM) using a triple quadrupole mass spectrometer (QQQ-MS) with a C18 column was used to determine their elution order. (TIF 710 kb)

Additional file 4: Molecular formula annotated metabolites and unannotated metabolites detected in exhaled breath condensate (EBC). These 37 out of 77 unique compounds were not matched to a database compound. Samples were analyzed in positive and negative ionization mode using LC-MS untargeted metabolomics on an SB-AQ analytical column. + indicates detected in positive ionization mode, - indicates detected in negative ionization mode. (PDF 38 kb)

Additional file 5: Tandem MS fragmentation patterns for six EBC metabolites. The mass spectral fragment peaks in red indicate the experimental results. The peaks in blue indicate the database matches based on standards. (PDF 36 kb)

Additional file 6: Putatively identified eicosanoids in EBC. 13 mL of pooled EBC from 107 asthmatic subjects was lyophilized, reconstituted in 20 μL of buffer, and analyzed using LC-MS based metabolomics. Metabolite peaks were extracted using Profinder and MassHunter software using exact mass and isotope ratios (Agilent). Detected peaks are indicated by single colored lines. Database isotope pattern and distribution is indicated by a circled red box. Matches with multiple adducts are indicated. (PDF 55 kb)

Additional file 7: Metabolite annotations and tandem MS fragmentation patterns of compound detected in EBC. EBC was collected from four groups of volunteers: healthy smokers, healthy non-smokers, non-smokers with nasal congestion, and non-smokers with the common cold. Samples were pooled, lyophilized, reconstituted in 20 μL of buffer, and analyzed using LC-MS based metabolomics. Metabolite peaks were extracted with Mass Hunter Profinder software (Agilent) using exact mass and isotope ratios. Tandem MS was performed, spectra was exported to NIST MS Search v2.2, and matched to the NIST14 Mass Spectral library. Fragments in red indicate EBC sample, fragments in blue indicate NIST standard reference spectra. (PDF 545 kb)

Additional file 8: Targeted eicosanoid analysis of EBC. EBC was collected from four groups of volunteers: healthy smokers, healthy non-smokers, non-smokers with nasal congestion, and non-smokers with the common cold. Samples were pooled, lyophilized, reconstituted in LC-MS buffer, and analyzed using targeted LC-MS on a triple quadruple mass spectrometer. (PDF 40 kb)

Additional file 9: Pathway analysis based on sample type. The compounds which were detected in each sample type were mapped to KEGG pathways using the online freeware pathway analysis software MBROLE. The compound names were based on database annotations using exact mass, isotope ratios and/or MSMS. Only pathways with hits ≥ 2 are listed. (PDF 278 kb)

## Abbreviations

11β-PGF_2α_: 11beta-prostaglandin F_2_ alpha
15R-PGF_2α_: 15R-prostaglandin F_2_ alpha
8-iso-PGF_2α_: 8-isoprostane-prostaglandin F_2_ alpha
APCI: Atmospheric pressure chemical ionization
BAL: Bronchoalveolar lavage
C18: Reverse phase chromatography with octadecyl carbon chain (C18)-bonded silica
CE: Collision energy
COPD: Chronic obstructive pulmonary disease
CysLT: Cysteinyl-leukotrienes
DiHDoHE: Dihydroxy-docosahexaenoic acid
EBC: Exhaled breath condensate
EIA: Enzyme immunoassay
ESI: Electrospray ionization
HDHA: Hydroxy-docosahexaenoic acid
HETE: Hydroxy-eicosatetraenoic acid
HILIC: Hydrophilic interaction chromatography
HMDB: Human metabolome database
KEGG: Kyoto encyclopedia of genes and genomes
LC: Liquid chromatography
LC-MS: Liquid chromatography mass spectrometry
LC-MS/MS: Liquid chromatography tandem mass spectrometry
LOD: Limit of detection
LOQ: Limit of quantitation
LTB_4_: Leukotriene B_4_
LTC_4_: Leukotriene C_4_
LTD_4_: Leukotriene D_4_
LTE_4_: Leukotriene E_4_
LXA_4_: Lipoxin A_4_
MS: Mass spectrometry
MSMS: Tandem mass spectrometry
PGE_2_: Prostaglandin E_2_
PGF2α: Prostaglandin F_2_ alpha
Protectin DX: 10(S),17(S)-DiHDoHE
QQQ: Triple quadrupole
Q-TOF: Quadrupole time-of-flight
R_V_D1: Resolvin D1
R_V_D2: Resolvin D2
SB-AQ: Stable bond (diisopropyl side chain group)
SRM: Selected reaction monitoring
TxB_2_: Thromboxane B_2_
