# Supplementary material for: Determining the presence of asthma-related molecules and salivary contamination in exhaled breath condensate
Source: Respir Res. 2017 Apr 12;18:57. doi: 10.1186/s12931-017-0538-5 (PMC5389118; doi:10.1186/s12931-017-0538-5)
Supplement: Supplementary file 1 — Targeted mass spectrometry parameters for eicosanoid analysis. MRM parameters, retention times, associated internal standards, and ionization modes used for lipid mediators by LC-MS/MS. IS: internal standard. (PDF 23 kb) [file 12931_2017_538_MOESM1_ESM.pdf]

Additional file 1: Targeted mass spectrometry parameters for eicosanoid analysis. MRM parameters, retention times, associated internal standards, and ionization modes used for lipid mediators by LC/MS/MS. IS: internal standard.

Compound	MRM	CE (V)	RT (min)	Internal standard	Ionization mode
8-iso-15R-PGF2a	353.2 -> 193.1	16	8.08	8-iso-PGF2a-d4	Negative
8-iso-PGF2a	353.2 -> 193.1	16	8.23	8-iso-PGF2a-d4	Negative
8-ISO-PGF2a-d4 (IS)	357.2 -> 197.1	26	8.20	NA	Negative
11B-PGF2a	353.2 -> 193.1	16	8.43	8-iso-PGF2a-d4	Negative
PGE2	351.2 -> 315.2	8	9.02	PGE2-d4	Negative
PGE2-d4 (IS)	355.2 -> 319.2	8	8.98	NA	Negative
15R-PGF2a	353.2 -> 309.2	16	9.03	PGF2a-d9	Negative
PGF2a	353.2 -> 309.2	16	9.12	PGF2a-d9	Negative
Resolvin D2-d5 (IS)	380.2 -> 141.0	13	9.30	NA	Negative
Resolvin D2	375.2 -> 141.0	13	9.35	Resolvin D2-d5	Negative
Resolvin D1	375.2 -> 141.0	13	10.13	Resolvin D2-d5	Negative
Lipoxin A4	351.2 -> 114.9	13	10.38	Resolvin D2-d5	Negative
LTD4-d5 (IS)	500.3 -> 176.8	18	11.37	NA	Positive
LTD4	495.3 -> 176.9	17	11.39	LTD4-d5	Positive
10(S),17(S)-DiHDoHE	359.2 -> 153.0	13	12.95	LTB4-d4	Negative
7S Maresin-1	359.2 -> 341.2	9	12.95	LTB4-d4	Negative
7R Maresin-1	359.2 -> 250.0	17	13.02	LTB4-d4	Negative
LTE4-d5	443.3 -> 338.2	18	13.06	NA	Positive
LTE4	438.2 -> 333.1	17	13.09	LTE4-d5	Positive
LTB4-d4	339.2 -> 197.1	13	13.47	NA	Negative
LTB4	335.2 -> 195.1	13	13.53	LTB4-d4	Negative
17(S)-HDHA	343.2 -> 281.2	9	17.40	9(S)-HODE-d4	Negative
14(S)-HDHA	343.2 -> 205.0	9	17.73	9(S)-HODE-d4	Negative
