# Supplementary figures and images for: Determining the presence of asthma-related molecules and salivary contamination in exhaled breath condensate
Source: Respir Res. 2017 Apr 12;18:57. doi: 10.1186/s12931-017-0538-5 (PMC5389118; doi:10.1186/s12931-017-0538-5)

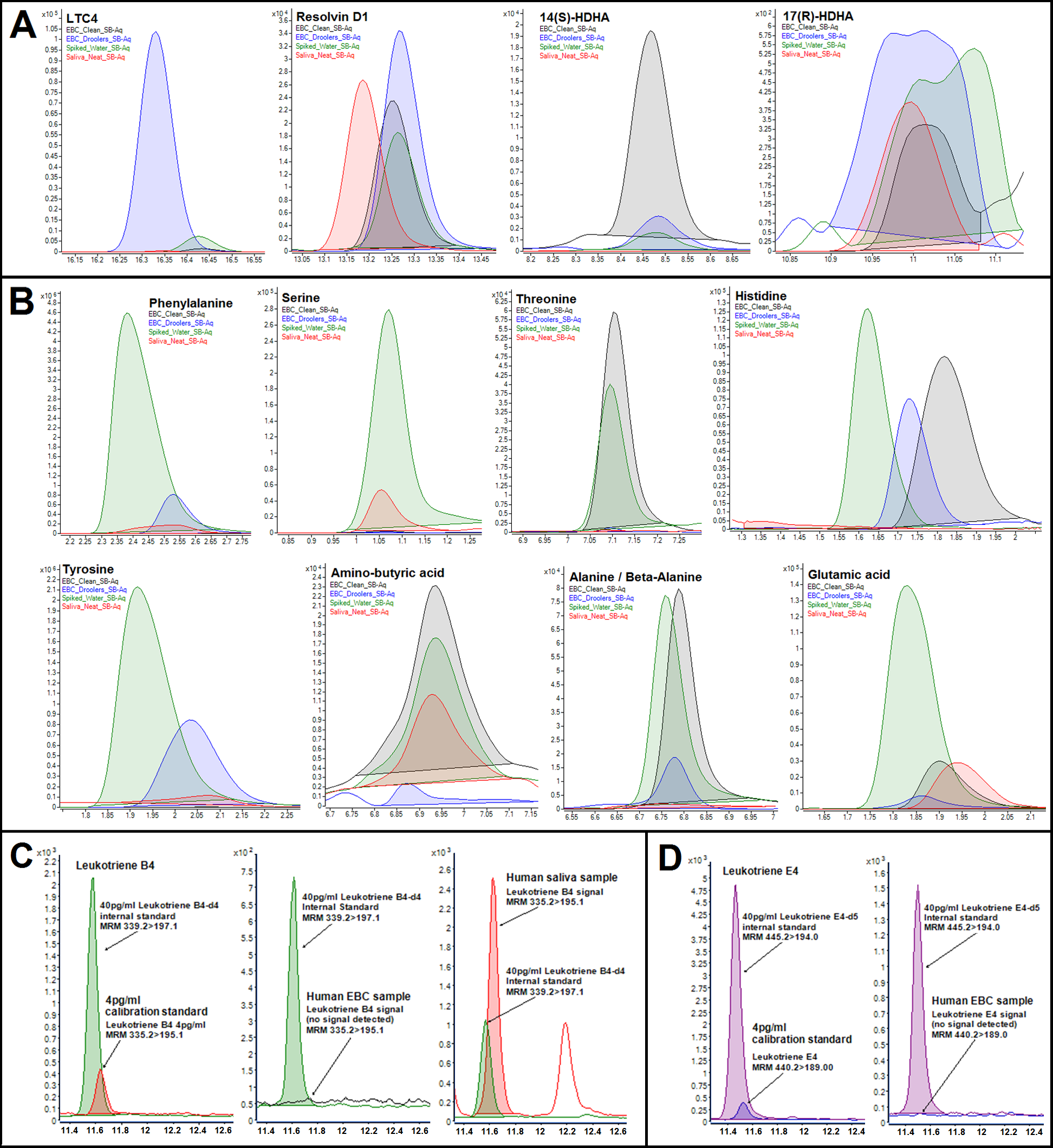

Supplement: Supplementary file 2 — Peak areas of selected amino acids and eicosanoids detected in EBC and/or saliva samples. (A) Peak areas of selected eicosanoids using untargeted metabolomics; (B) Peak areas of selected amino acids in spiked water (green), clean-EBC (black), saliva-EBC (blue), and saliva (red) using untargeted metabolomics. A control water sample which was spiked with known concentrations of amino acids and eicosanoids was used to confirm the identities of these compounds in the saliva and EBC samples using exact mass, isotope ratios and retention time. (C) Peak area of LTB4 in internal standard, EBC and saliva using targeted analysis. (D) Peak area of LTE4 in internal standard and EBC using targeted analysis. Peak areas were extracted using MassHunter Quantitative Analysis software (Agilent). y-axis: mass spectral counts; x-axis: retention time. Starting volumes for untargeted metabolomics was 11.5 mL (clean-EBC) and 7.5 mL (saliva-EBC) with final volume of 20 μL and injection volume of 5 μL. Starting volume for targeted analysis was 1 mL saliva or EBC with an injection volume of 100 μL. (TIF 1669 kb) [file 12931_2017_538_MOESM2_ESM.tif]

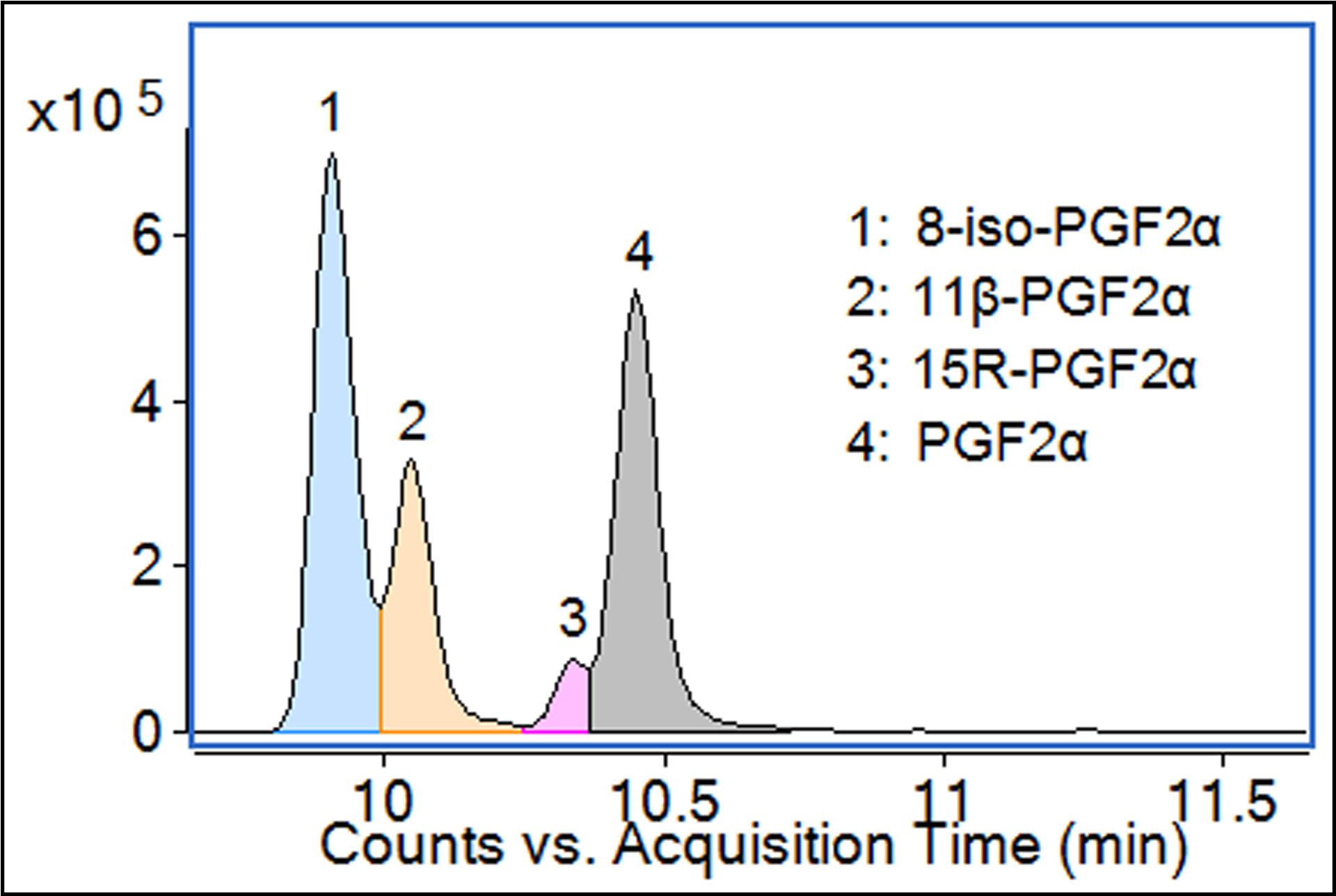

Supplement: Supplementary file 3 — Separation of PGF2α isomers in spiked control water. Samples were injected onto an SB-AQ analytical column. Since the four isomers could not be differentiated using untargeted analysis, multiple reaction monitoring (MRM) using a triple quadrupole mass spectrometer (QQQ-MS) with a C18 column was used to determine their elution order. (TIF 710 kb) [file 12931_2017_538_MOESM3_ESM.tif]
