# Supplementary material for: Determining the presence of asthma-related molecules and salivary contamination in exhaled breath condensate
Source: Respir Res. 2017 Apr 12;18:57. doi: 10.1186/s12931-017-0538-5 (PMC5389118; doi:10.1186/s12931-017-0538-5)
Supplement: Supplementary file 4 — Molecular formula annotated metabolites and unannotated metabolites detected in exhaled breath condensate (EBC). These 37 out of 77 unique compounds were not matched to a database compound. Samples were analyzed in positive and negative ionization mode using LC-MS untargeted metabolomics on an SB-AQ analytical column. + indicates detected in positive ionization mode, - indicates detected in negative ionization mode. (PDF 38 kb) [file 12931_2017_538_MOESM4_ESM.pdf]

Additional file 4: Molecular formula annotated metabolites and unannotated metabolites detected in exhaled breath condensate (EBC). These 37 out of 77 unique compounds were not matched to any compounds in the database. Samples were analyzed in positive and negative ionization mode using LC-MS untargeted metabolomics on an SB-AQ analytical column. + indicates detected in positive ionization mode, - indicates detected in negative ionization mode.

Compound Annotation	Mode	RT	Mass	m/z	Adduct	Peak Area
C7 H19 N4 O13 P3	-	12.633	460.0180	459.0093	[M-H]-	7652
C8 H27 N7 O4 P2	+	10.716	347.1618	386.1271	[M+K]+	8351
C9 H24 N6 O3	+	8.704	264.1916	265.2000	[M+H]+	67512
C11 H21 N2 O2 P	+	9.828	244.1351	245.1367	[M+H]+	42855
C12 H27 N9 O	+	9.921	313.2346	352.1999	[M+Na]+	17817
C18 H25 O P	+	12.732	288.1670	289.1683	[M+H]+	49374
C18 H46 N6 P4	+	8.246	470.2712	471.2753	[M+H]+	37164
C22 H26 O7	+	9.614	402.1686	403.1747	[M+H]+	26353
C22 H33 N P2	+	7.609	373.2139	374.2182	[M+H]+	31172
C22 H40 O2 P2	+	7.804	398.2503	399.2518	[M+H]+	27711
C23 H11 N3	+	9.442	329.0968	368.0582	[M+K]+	36431
C24 H37 N8 O P	+	8.602	484.2865	485.2933	[M+H]+	113802
C25 H51 N8 O3 P S	+	8.097	574.3518	575.3556	[M+H]+	104092
C26 H56 N13 P S	+	8.164	613.4223	636.4137	[M+Na]+	68529
C27 H60 N16 O2 S2	+	9.149	704.4507	727.4419	[M+Na]+	37792
C32 H64 N6 O6 P2	+	8.756	690.4305	713.4247	[M+Na]+	2768
C34 H59 N12 P3	+	7.632	728.4122	729.4123	[M+H]+	131
C37 H82 N19 O3 P S	+	10.277	903.6257	926.6201	[M+Na]+	378340
C38 H42 N2 O2	+	8.404	558.3259	559.3220	[M+H]+	221488
C39 H81 N41 O	+	9.734	1139.7530	1162.7457	[M+Na]+	40278
C39 H87 N21 O5 S2	+	9.209	993.6629	1016.6553	[M+Na]+	53419
C41 H90 N19 O5 P S	+	10.364	991.6787	1014.6743	[M+Na]+	338107
C41 H90 N19 O6 P S	+	9.573	1007.6778	1030.6740	[M+Na]+	136738
C42 H89 N11 O2 P2 S2	+	9.119	905.6093	928.6011	[M+Na]+	99423
C43 H90 N2 O18 S	+	9.179	954.5841	478.2982	[M+2H]+2	68975
C47 H96 N12 O15 S	+	9.674	1100.6800	551.3372	[M+2H]+2	65249
C51 H71 N3 O S	+	8.906	773.5229	796.5205	[M+Na]+	136495
C52 H114 N5 O11 P3 S	+	10.060	1109.7420	1132.7241	[M+Na]+	117557
C55 H109 N21 O10 S	+	10.603	1255.8365	1278.8394	[M+Na]+	91667
C57 H122 N15 O8 P3 S	+	11.049	1269.8519	657.9166	[M+2Na]+2	154146
C57 H124 N5 O13 P3 S	+	10.573	1211.8108	1234.7935	[M+Na]+	129906
C64 H4 O33	+	10.633	1299.8640	1322.8550	[M+Na]+	55829
C68 H9 N2 O23 P3	+	11.061	1313.8812	1336.8677	[M+Na]+	105448
122.0915@6.8110003	+	6.811	122.0915	145.0802	[M+Na]+	47117
172.2097@1.768	+	1.768	172.2097	173.2177	[M+H]+	28234
386.403@12.376	+	12.376	386.4030	387.4111	[M+H]+	12334
418.1606@9.547	+	9.547	418.1606	419.1674	[M+H]+	25790
