# Supplementary material for: Determining the presence of asthma-related molecules and salivary contamination in exhaled breath condensate
Source: Respir Res. 2017 Apr 12;18:57. doi: 10.1186/s12931-017-0538-5 (PMC5389118; doi:10.1186/s12931-017-0538-5)

Additional file 5: Tandem MS fragmentation patterns for six EBC metabolites. The mass spectral fragment peaks in red indicate the experimental results. The peaks in blue indicate the database matches based on standards.

Citric acid ($C_6H_8O_7$), negative mode, RT = 1.693 minutes

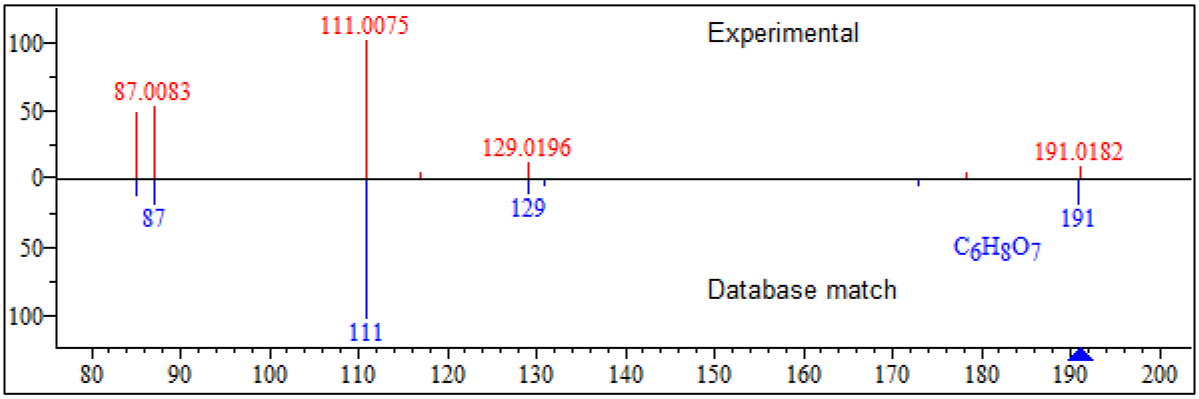

Succinic acid ($C_4H_6O_4$), negative mode, RT = 1.830 minutes

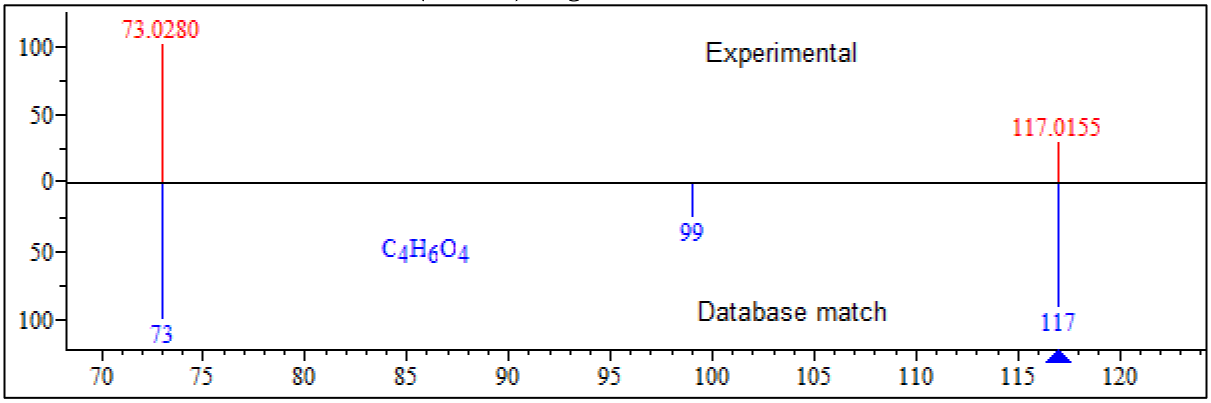

Acetylsalicylic acid ($C_9H_8O_4$), negative mode, RT = 6.957 minutes

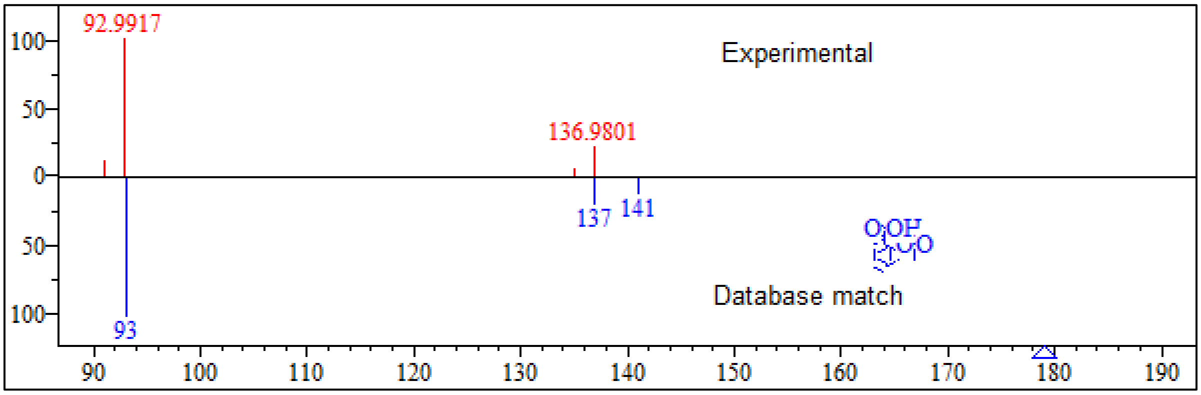

Shikimic acid ($C_7H_{10}O_5$), negative mode, RT = 27.634 minutes

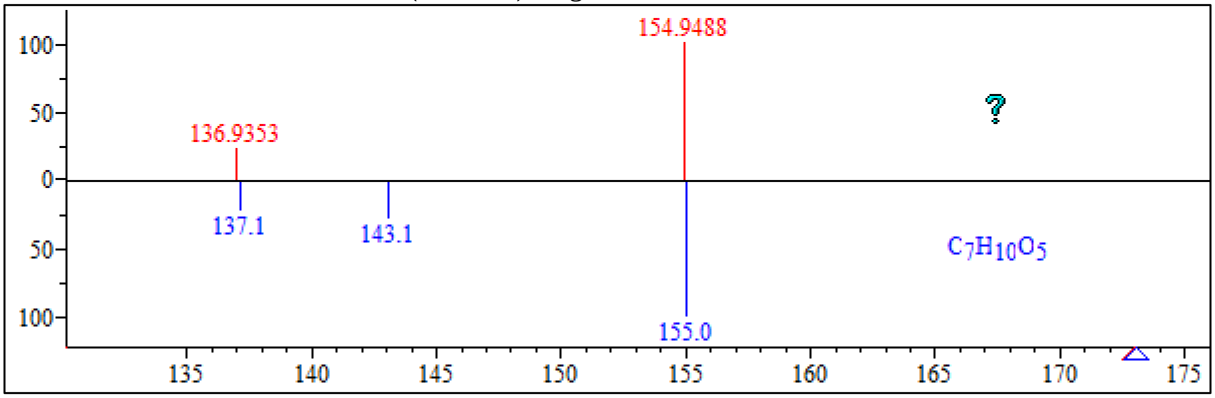

3,4-Furandicarboxylic acid ($C_6H_4O_5$), negative mode, RT = 31.702 minutes

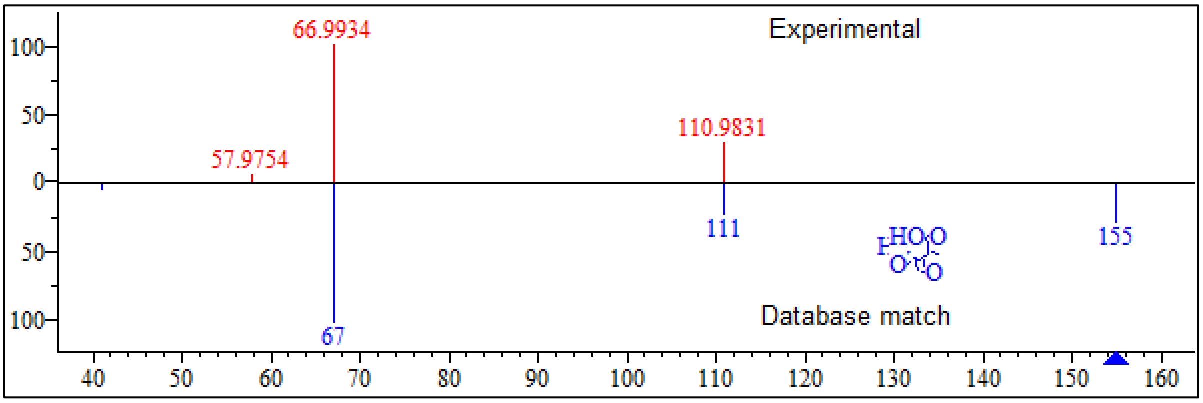

4-Chloro-L-phenylalanine ($C_9H_{10}ClNO_2$), negative mode, RT = 31.518 minutes

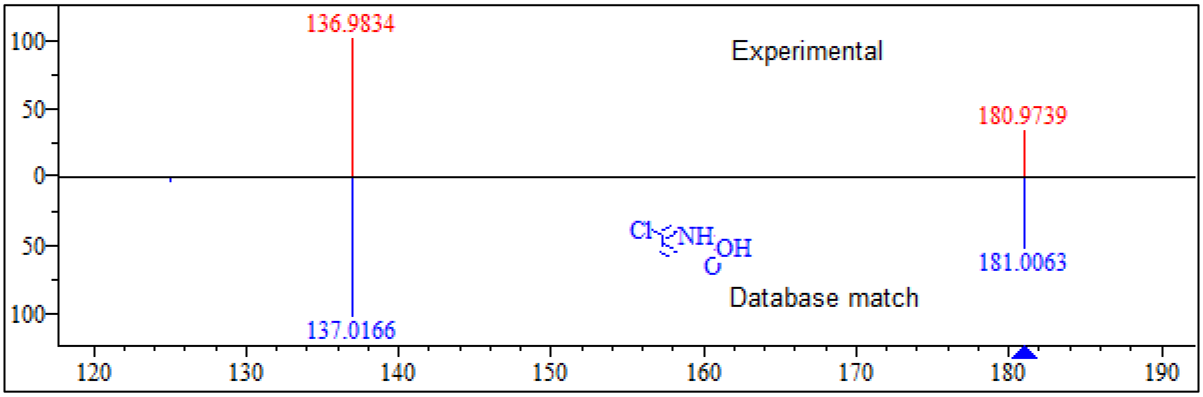

Supplement: Supplementary file 5 — Tandem MS fragmentation patterns for six EBC metabolites. The mass spectral fragment peaks in red indicate the experimental results. The peaks in blue indicate the database matches based on standards. (PDF 36 kb) [file 12931_2017_538_MOESM5_ESM.pdf]
