# Supplementary material for: Determining the presence of asthma-related molecules and salivary contamination in exhaled breath condensate
Source: Respir Res. 2017 Apr 12;18:57. doi: 10.1186/s12931-017-0538-5 (PMC5389118; doi:10.1186/s12931-017-0538-5)

Additional file 6: Putatively identified eicosanoids in EBC. 13mL of pooled EBC from 107 asthmatic subjects was lyophilized, reconstituted in 20µL of buffer, and analyzed using LC-MS based metabolomics. Metabolite peaks were extracted using Profinder and Mass Hunter software using exact mass and isotope ratios (Agilent). Detected peaks are indicated by single colored lines. Database isotope pattern and distribution is indicated by a circled red box. Matches with multiple adducts are indicated.

Leukotriene E3 (sodium adducts plus a loss of water at 446 m/z)

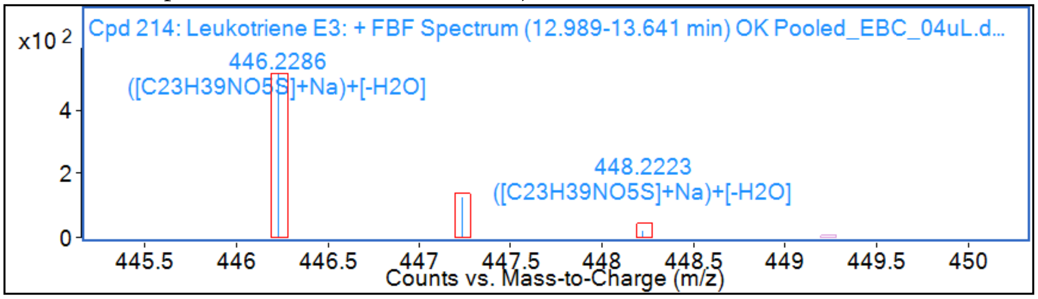

11-trans-LTE4 (protonated with a loss of water at 422 m/z; minor cluster of ammoniated peaks at 457 m/z)

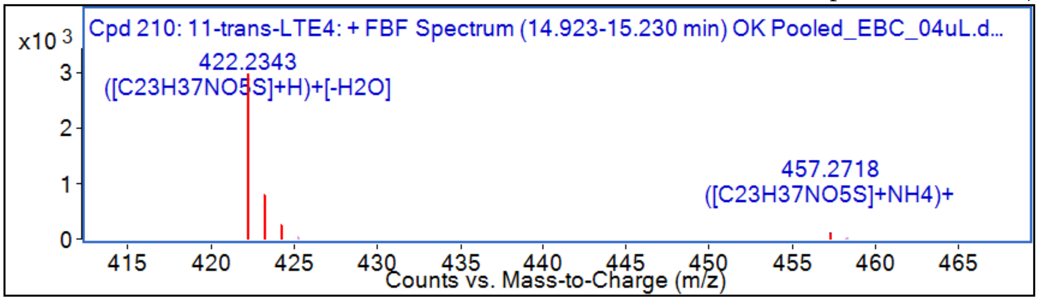

Thromboxane (ammoniated peaks at 314 m/z; sodiated adduct with a loss of water at 301 m/z)

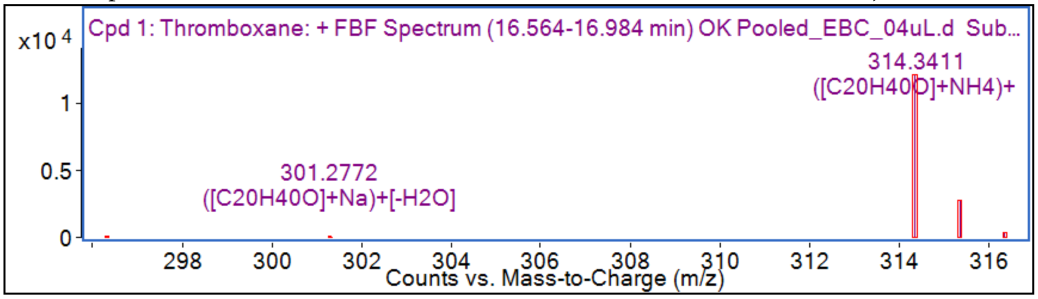

12-oxo-LTB4 (protonated with a loss of water at 317 m/z; minor peaks protonated at 335 & ammoniated at 352 m/z)

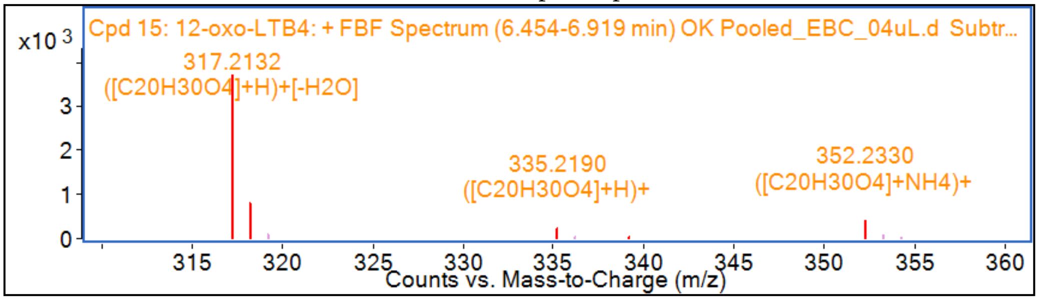

11-trans-LTC4 (sodiated dimer at 1273 m/z)

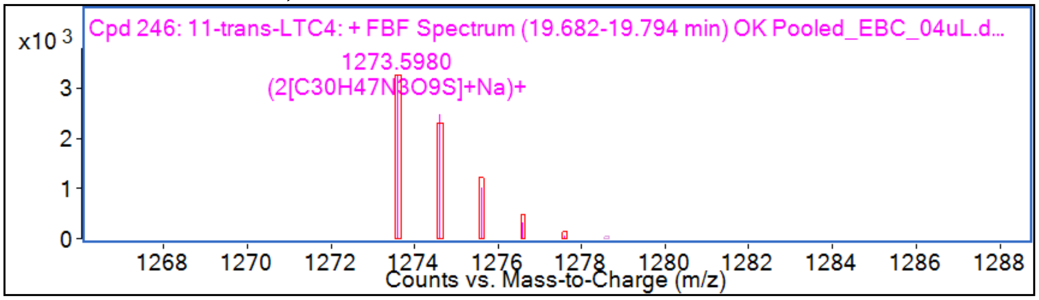

Supplement: Supplementary file 6 — Putatively identified eicosanoids in EBC. 13 mL of pooled EBC from 107 asthmatic subjects was lyophilized, reconstituted in 20 μL of buffer, and analyzed using LC-MS based metabolomics. Metabolite peaks were extracted using Profinder and MassHunter software using exact mass and isotope ratios (Agilent). Detected peaks are indicated by single colored lines. Database isotope pattern and distribution is indicated by a circled red box. Matches with multiple adducts are indicated. (PDF 55 kb) [file 12931_2017_538_MOESM6_ESM.pdf]
