# Supplementary material for: Determining the presence of asthma-related molecules and salivary contamination in exhaled breath condensate
Source: Respir Res. 2017 Apr 12;18:57. doi: 10.1186/s12931-017-0538-5 (PMC5389118; doi:10.1186/s12931-017-0538-5)
Supplement: Supplementary file 7 — Metabolite annotations and tandem MS fragmentation patterns of compound detected in EBC. EBC was collected from four groups of volunteers: healthy smokers, healthy non-smokers, non-smokers with nasal congestion, and non-smokers with the common cold. Samples were pooled, lyophilized, reconstituted in 20 μL of buffer, and analyzed using LC-MS based metabolomics. Metabolite peaks were extracted with Mass Hunter Profinder software (Agilent) using exact mass and isotope ratios. Tandem MS was performed, spectra was exported to NIST MS Search v2.2, and matched to the NIST14 Mass Spectral library. Fragments in red indicate EBC sample, fragments in blue indicate NIST standard reference spectra. (PDF 545 kb) [file 12931_2017_538_MOESM7_ESM.pdf]

Additional file 7: Metabolite annotations and tandem MS fragmentation patterns of compound detected in EBC. EBC was collected from four groups of volunteers: healthy smokers, healthy non-smokers, non-smokers with nasal congestion, and non-smokers with the common cold. Samples were pooled, lyophilized, reconstituted in 20µL of buffer, and analyzed using LC-MS based metabolomics. Metabolite peaks were extracted with Mass Hunter Profinder software (Agilent) using exact mass and isotope ratios. Tandem MS was performed, spectra was exported to NIST MS Search v2.2, and matched to the NIST14 Mass Spectral library. Fragments in red indicate EBC sample, fragments in blue indicate NIST standard reference spectra.

**Compound**	**Smkr**	**Nasal**	**Cold**	**Hlthy**	**RT (mins)**	**Mass**	**Formula**	**Mode**	**NIST MF**	**NIST RMF**	**KEGG**	**PubChem**
(R)-4'-Deoxyindenestrol	X	X	X	X	6.620	250.1358	C18 H18 O	+			C14541	11954028
(R)-5-Deoxyindenestrol	X	X	X	X	7.839	250.1358	C18 H18 O	+			C14543	11954030
N-Acetyl-D-fucosamine	X	X	X	X	8.079	205.0950	C8 H15 N O5	+			C15480	11954186
Heptadecatrienoic acid	X	X	X	X	8.080	264.2089	C17 H28 O2	+			C16344	16061034
(+)-Prosopinine	X	X	X	X	10.422	313.2609	C18 H35 N O3	+			C10167	442654
1,3-Dicyclohexylurea	X	X	X	X	8.876	224.1889	C13 H24 N2 O	+	713	880		4277
10-Hydroxydecanoic acid	X	X	X	X	7.523	216.1357	C10 H20 O3	-			C02774	74300
11-Aminoundecanoic acid	X	X	X	X	7.418	229.1680	C11 H23 N O2	-				17083
1-Hydroxy-2-naphthoic acid	X	X	X	X	6.258	188.0472	C11 H8 O3	+			C03203	6844
2-(Methylthiomethyl)-3-phenyl-2-propenal	X	X	X	X	6.388	230.0169	C11 H12 O S	+				6435634
2,4-Diamino-6-nitrotoluene	X	X	X	X	7.112	167.0703	C7 H9 N3 O2	+			C16396	96177
13,14-dihydro Prostaglandin F1a	X	X	X	X	6.814	358.2736	C20 H38 O5	+				
2-Anthramine	X	X	X	X	6.672	193.0897	C14 H11 N	+			C14417	11937
2-Amino-3,7-dideoxy-D-threo-hept-6-ulosonic acid	X	X	X	X	6.380	213.0620	C7 H13 N O5	+			C16850	25137912
2-Carboxy-4-dodecanolide	X	X	X	X	6.301	282.1449	C13 H22 O4	+				
2-Methylisoborneol	X	X	X	X	10.458	185.1778	C11 H20 O	+				16913
4-Heptadecene	X	X	X	X	10.081	255.2924	C17 H34	+				
3-Deoxyestrone	X	X	X	X	7.300	254.1676	C18 H22 O	+			C14972	5882
4-Vinylcyclohexene	X	X	X	X	7.491	233.2141	C8 H12	+				7499
5-Nitro-2-furfuryl methyl ether	X	X	X	X	6.855	139.0270	C6 H7 N O4	-			C14293	11469
9-(Methylthio)nonanenitrile	X	X	X	X	6.799	185.1234	C10 H19 N S	+				93987
Allotetrahydrodeoxycorticosterone	X	X	X	X	6.756	351.2768	C21 H34 O3	+			C13713	101771
beta-Santonin	X	X	X	X	7.790	286.1171	C15 H18 O3	+			C09545	237619
Butoctamide hydrogen succinate	X	X	X	X	5.933	375.2285	C16 H29 N O5	-			C12946	2486
C14 sphingosine	X	X	X	X	9.628	271.2141	C14 H29 N O2	-				
Arachidonoyl-1-thio-Glycerol	X	X	X	X	6.303	428.3085	C23 H38 O3 S	+				
cis-Hydroxy Perhexiline	X	X	X	X	7.714	331.2304	C19 H35 N O	+				
Citronellyl formate	X	X	X	X	8.918	184.1461	C11 H20 O2	-			C12295	7778
Diethylene glycol	X	X	X	X	1.201	202.0454	C4 H10 O3	-			C14689	8117
Dimethyl suberate	X	X	X	X	6.940	202.1207	C10 H18 O4	-			C17803	15611
Dizocilpine	X	X	X	X	7.245	221.1209	C16 H15 N	+			C13737	1207
Ethyl 3-(N-butylacetamido)propionate	X	X	X	X	6.857	215.1522	C11 H21 N O3	-				
Ethyl 3,4,5-trimethoxybenzoate	X	X	X	X	6.664	219.1691	C12 H16 O5	+				231162
Furmecyclox	X	X	X	X	13.492	251.1517	C14 H21 N O3	-				43359
Enhydrin	X	X	X	X	7.002	464.1678	C23 H28 O10	-			C09405	325606
Ganoderic acid Mg	X	X	X	X	6.451	602.3877	C35 H54 O8	+				
Gnididilatin	X	X	X	X	7.590	669.3502	C37 H48 O10	+			C09101	442032
Gomphrenin-I	X	X	X	X	11.228	646.1258	C24 H26 N2 O13	-			C08544	4480887
Gossypetin 3,5,8,3',4'-pentamethyl ether	X	X	X	X	0.979	388.1127	C20 H20 O8	+				
Heptanal	X	X	X	X	6.502	210.0870	C7 H14 O	-			C14390	8130
Icosenoic acid	X	X	X	X	13.666	327.3138	C20 H38 O2	+			C16526	5282768
Indicumenone	X	X	X	X	12.421	234.1620	C15 H24 O3	-			C17612	
12,14-Pentacosadiynoic acid	X	X	X	X	6.122	374.3185	C25 H42 O2	-				9543642
Isodictamnine	X	X	X	X	6.491	199.0637	C12 H9 N O2	+			C10697	442913
N-cis-tetradec-9Z-enoyl-L-Homoserine lactone	X	X	X	X	10.715	309.2301	C18 H31 N O3	+				
Oxprenolol	X	X	X	X	8.085	265.1652	C15 H23 N O3	-				4631
Pantothenol	X	X	X	X	6.088	187.1208	C9 H19 N O4	-			C05944	4678
p-Cymene	X	X	X	X	9.382	152.1205	C10 H14	+			C06575	7463
Pulcherriminic acid	X	X	X	X	6.774	238.1332	C12 H20 N2 O4	-				3083664
Tamarixetin	X	X	X	X	6.420	298.0486	C16 H12 O7	-			C10188	482576
Teniposide	X	X	X	X	11.221	656.1547	C32 H32 O13 S	-			C11153	5396
Tremetone	X	X	X	X	1.200	264.0154	C13 H14 O2	-			C08992	78673
Ureidoglycine	X	X	X	X	0.980	380.0903	C3 H7 N3 O3	-			C02091	503
Zeatin	X	X	X	X	7.333	219.1122	C10 H13 N5 O	+			C00371	15419
3-Cyano-6-methoxycoumarin	X				5.936	201.0426	C11 H7 N O3	+	508	970		

1,2-Dimethylimidazole	X				1.538	96.0687	C5 H8 N2	+	520	987		15617
1-Hexadecylamine	X				9.087	241.277	C16 H35 N	+	398	858		8926
2,6-Di-tert-butyl-4-hydroxymethylphenol	X				6.806	236.1776	C15 H24 O2	+	823	922		6929
2-Aminobenzamide	X				1.58	136.0637	C7 H8 N2 O	+	425	977		6942
2-Amino-m-cresol	X				0.811	123.0684	C7 H9 N O	+	560	999		3014138
3-Methylhistidine	X				0.809	169.0851	C7 H11 N3 O2	+	383	860	C01152	64969
Arginine	X				0.81	174.1117	C6 H14 N4 O2	+	610	908	C02385	232
Carnosine	X				0.809	226.1066	C9 H14 N4 O3	+	262	841	C00386	9369
p,p'-Ditolylamine	X				6.038	197.1204	C14 H15 N	+	597	933		69293
Tryptophan	X				4.079	204.0899	C11 H12 N2 O2	+	679	926		
4-Hydroxy-3-methoxyphenethyl alcohol	X				5.255	166.063	C9 H12 O3	+	560	999		16928
4-Imidazoleacrylic acid	X				5.023	138.0429	C6 H6 N2 O2	+	557	998		1178
5-Aminosalicylic acid	X				1.612	153.0426	C7 H7 N O3	+	553	997		4075
D-erythro-Sphinganine	X				5.186	301.2981	C18 H39 N O2	+	51	299		91486
Triethyl citrate	X				7.519	276.1209	C12 H20 O7	+	360	945		6506
Acetylarginine	X				4.91	216.1222	C8 H16 N4 O3	+	404	910		
5-Methoxytryptophan	X				6.124	234.1004	C12 H14 N2 O3	+	532	993		119802
1,2-Benzenecarboxylic acid	X		X	X	12.835	148.016	C8 H4 O3	+	679	946		181977
2-Butyl-4-propylthiazole	X		X	X	7.548	205.0902	C10 H17 N S	+				
3-Hydroxymorphinan	X			X	5.004	243.1623	C16 H21 N O	+	535	989		
Gabaculine	X		X	X	6.387	121.0524	C7 H9 N O2	-			C12110	3445
Thyrotropin releasing hormone	X		X		9.248	362.1702	C16 H22 N6 O4	-			C03958	4968
12-Nitrolinoleic acid	X	X	X		6.303	325.2245	C18 H31 N O4	+				
12-O-Palmitoyl-16-hydroxyphorbol 13-acetate	X	X			5.803	756.4039	C38 H60 O9	-			C09153	124127
15(S)-HPETE	X	X		X	10.185	264.2089	C20 H32 O4	+			C05966	1440
2-chloro-2-iodoacetamide	X	X	X		8.499	278.9149	C2 H3Cl I N O	-				
2-Oxo-4-hydroxy-5-aminovalerate	X	X			1.604	129.0426	C5 H9 N O4	-			C05941	440852
2-Oxo-6-methylthiohexanoic acid	X	X		X	8.085	193.0780	C7 H12 O3 S	+			C17216	44237195
2S-Amino-tridecanoic acid	X	X		X	8.835	257.1986	C13 H27 N O2	+			C13795	656741
3,4-Dihydrocadalene	X	X	X		10.501	200.1563	C15 H20	+				
3-Hydroxy-2-(4-morpholinylmethyl)estra-1,3,5(10)-trien-17-one	X	X			5.178	431.1463	C23 H31 N O3	-			C14926	235463
6-hydroxy caproic acid	X	X	X		6.131	174.0890	C6 H12 O3	-				14490
6-Hydroxy-3,7-dimethyloctanoate	X	X		X	8.518	188.1410	C10 H20 O3	-			C18067	
Bisdiphosphoinositol tetrakisphosphate	X	X	X		0.771	705.8145	C6 H20 O24 P8	-				25245165
Carteolol	X	X	X		6.385	274.1687	C16 H24 N2 O3	-			C06874	2583
Chalcogran	X	X			6.409	156.1151	C9 H16 O2	-			C08471	441558
Cybutryne	X	X	X		2.275	281.1293	C11 H19 N5 S	-				91590
Deaminohydroxyblasticidin S	X	X			5.769	483.2076	C17 H25 N7 O6	-			C03860	629
Homoserine	X	X	X		6.707	215.0406	C4 H9 N O3	-				779
Dodecanedioic acid	X	X	X		8.186	230.1517	C12 H22 O4	-			C02678	12736
Eleganin	X	X	X		7.474	434.1566	C22 H26 O9	-			C09401	5281440
Ethirimol	X	X			6.063	532.2984	C11 H19 N3 O	-				32152
gamma-Chaconine	X	X	X		6.247	601.3965	C33 H53 N O6	-				160496
Hallactone B	X	X	X		9.784	468.1098	C20 H24 O9 S	-			C09105	442036
Hydnocarpic acid	X	X	X		9.749	252.2089	C16 H28 O2	+			C16795	110680
Levan	X	X	X		1.016	504.1672	C18 H32 O16	-			C06215	440946
Lys Lys Gly	X	X	X		5.934	377.2258	C14 H29 N5 O4	-				
Met Trp Glu	X	X			10.589	492.1678	C21 H28 N4 O6 S	-				
Meteloidine	X	X			5.385	315.1683	C13 H21 N O4	-			C10861	443006
N-Acetylleucine	X	X			5.710	173.1055	C8 H15 N O3	-				1995
N-Methylpelletierine	X	X	X		6.429	201.1364	C9 H17 N O	-			C06184	86786
PA(22:2/0:0)	X	X	X		6.118	490.3105	C25 H47 O7 P	-				
Pachymic acid	X	X			5.487	642.3713	C33 H52 O5	-			C17044	316448
Paromamine	X	X	X		5.176	369.1758	C12 H25 N3 O7	-			C01743	439560
PI(12:0/12:0)	X	X	X		5.703	698.3983	C33 H63 O13 P	-				
Tetrahydrodipicolinate	X	X	X		7.780	153.0427	C7 H9 N O4	-				5460758
Threonine	X	X	X		8.939	233.0513	C4 H9 N O3	-				205
1,8-Diazacyclotetradecane-2,9-dione		X	X		6.118	498.3410	C12 H22 N2 O2	-			C04277	16
13,14-dihydroxy-docosanoic acid		X	X		10.059	394.3109	C22 H44 O4	+				102450
(R,S)-Tetrahydrobenzylisoquinoline		X	X	X	7.463	223.1366	C16 H17 N	+			C05201	98468
2-Methylpropanal oxime		X	X	X	6.879	87.0683	C4 H9 N O	+			C03219	5324281
3-Hydroxy-4-methoxybenzyl alcohol		X		X	5.078	154.063	C8 H10 O3	+	479	804		78089
3-Oxosteroid		X	X	X	8.419	291.2562	C19 H30 O	+			C01876	439594

4-Dodecylphenol		X	X	X	11.465	279.2561	C18 H30 O	+			C14245	66030
5-(1-oxopropan-2-yl)isolongifol-5-ene		X	X	X	11.149	260.2132	C18 H28 O	+				
6-(2-Chloroallylthio)purine		X	X		7.239	247.9896	C8 H7 Cl N4 S	+			C15397	3247797
Aniracetam		X	X	X	8.231	201.0790	C12 H13 N O3	-			C13355	2196
Benzylamine		X			8.278	107.0735	C7 H9 N	+	539	983	C15562	7504
1α,24,25,28-tetrahydroxyvitamin D2		X	X		6.213	460.3189	C28 H44 O5	-				
MG(18:1)		X	X	X	8.908	373.3185	C21 H40 O4	+				
Miotine		X	X	X	6.023	222.1369	C12 H18 N2 O2	+			C11763	46092
O-decanoyl-R-carnitine		X	X		6.305	361.2453	C17 H33 N O4	-				
Palmitoylglycine		X		X	9.931	335.2469	C18 H35 N O3	+				151008
PC(18:1/22:6)		X	X	X	6.813	831.5785	C48 H82 N O8 P	+				
PG(18:4/20:4)		X	X	X	12.183	790.4734	C44 H71 O10 P	+				
Purine		X			8.811	120.0436	C5 H4 N4	+	791	993	C15587	1044
S-(4,5-Dihydro-2-methyl-3-furanyl) ethanethioate		X	X	X	5.915	186.0353	C7 H10 O2 S	-				5463955
Tris(2-butoxyethyl)phosphate		X	X	X	12.185	398.2433	C18 H39 O7 P	+	829	922	C14446	6540
3-Acetyl-8-methoxycoumarin			X		5.846	218.0579	C12 H10 O4	+	661	881		
1-(2-Pyrimidyl)piperazine				X	5.37	164.1062	C8 H12 N4	+	559	998		88747
1-Butylimidazole			X		4.977	124.1	C7 H12 N2	+	449	852		61347
2-Hydroxy-6'-methoxyacetophenone				X	10.183	166.063	C9 H10 O3	+	153	601		69709
4-(4-Aminophenoxy)aniline				X	5.892	200.095	C12 H12 N2 O	+	454	893		
6-Hydroxymelatonin				X	1.184	248.1161	C13 H16 N2 O3	+	422	890	C05643	1864
Atractyloside C			X	X	13.415	442.2598	C21 H36 O7	-				
Benzofuran			X	X	7.921	118.0419	C8 H6 O	+			C14512	9223
α-Lipoic acid			X		5.666	206.0435	C8 H14 O2 S2	+	476	775	C00725	864
Ephedrine			X		31.317	165.1154	C10 H15 N O	+	560	999	C01575	5032
Hexaethylene glycol			X		5.443	282.1679	C12 H26 O7	+	765	949		17472
Arogenate			X	X	6.828	227.0775	C10 H13 N O5	-				193887
Tetradecyamine			X		7.876	213.2456	C14 H31 N	+	522	957		

EBC from healthy non-smokers

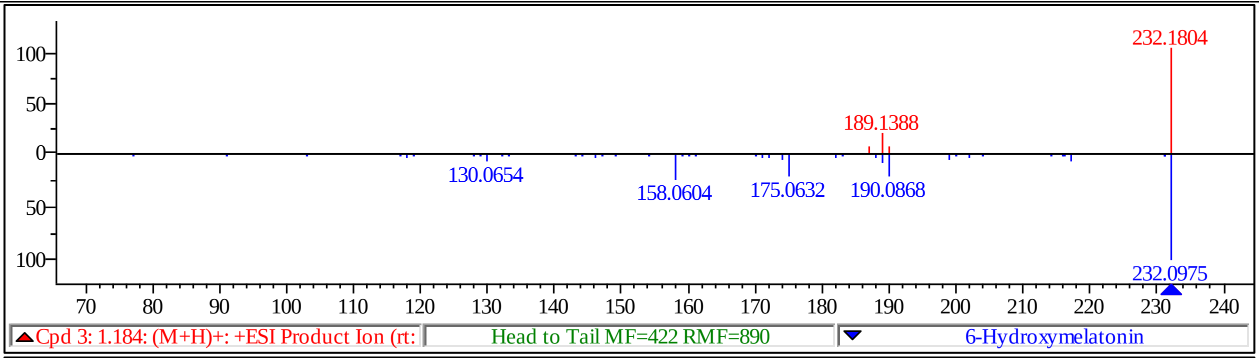

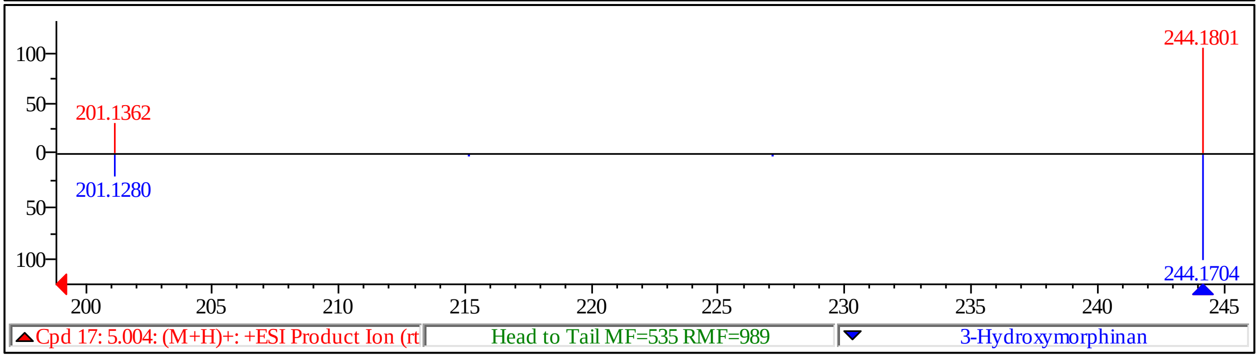

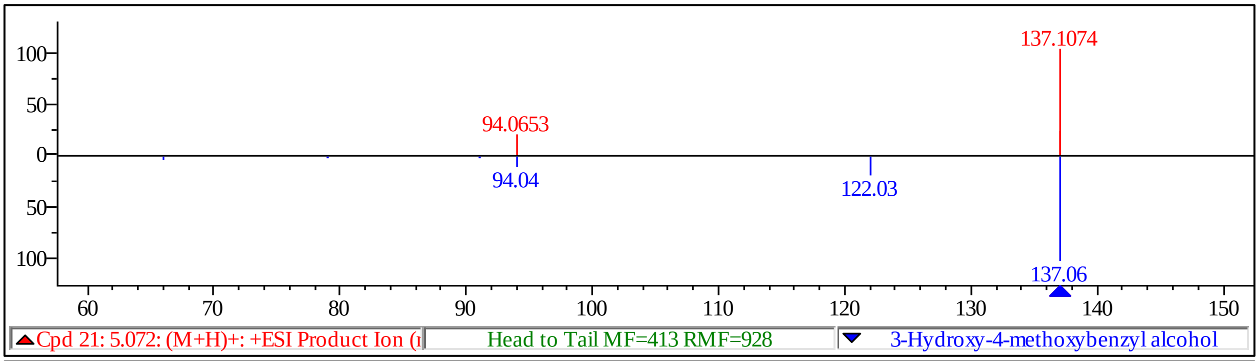
137.1074
94.0653
94.04
122.03
137.06
Cpd 21: 5.072: (M+H)+: +ESI Product Ion (r
Head to Tail MF=413 RMF=928
3-Hydroxy-4-methoxybenzyl alcohol

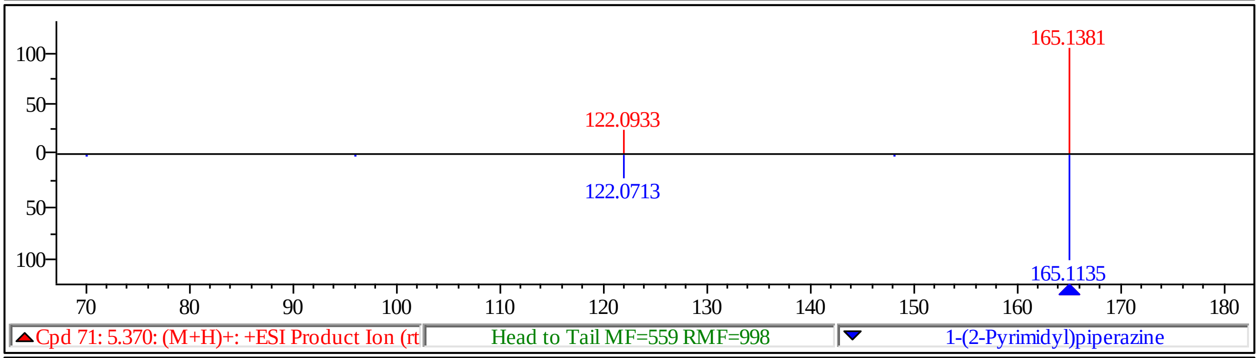
165.1381
122.0933
122.0713
165.1135
Cpd 71: 5.370: (M+H)+: +ESI Product Ion (rt
Head to Tail MF=559 RMF=998
1-(2-Pyrimidyl)piperazine

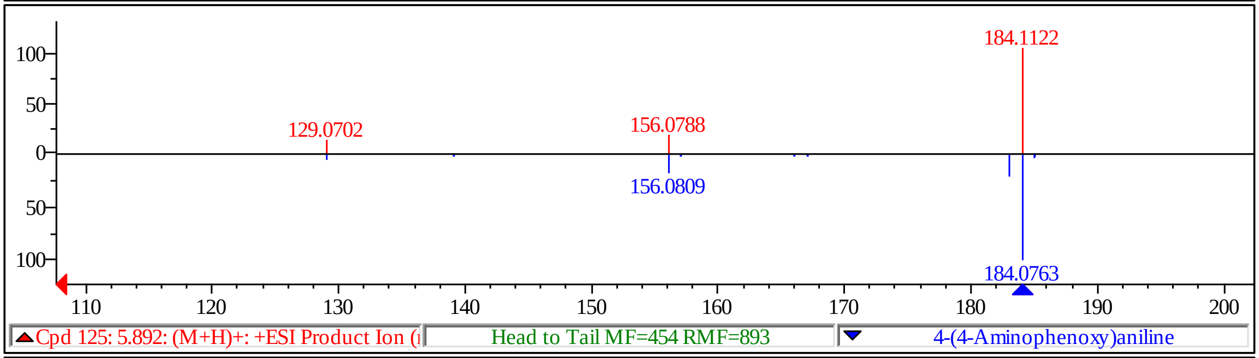
184.1122
129.0702
156.0788
156.0809
184.0763
Cpd 125: 5.892: (M+H)+: +ESI Product Ion (r
Head to Tail MF=454 RMF=893
4-(4-Aminophenoxy)aniline

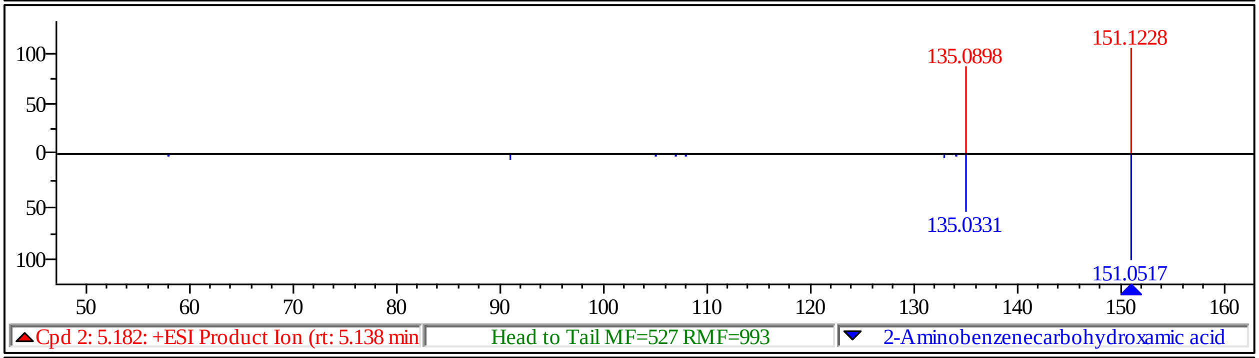
151.1228
135.0898
135.0331
151.0517
Cpd 2: 5.182: +ESI Product Ion (rt: 5.138 min
Head to Tail MF=527 RMF=993
2-Aminobenzenecarbohydroxamic acid

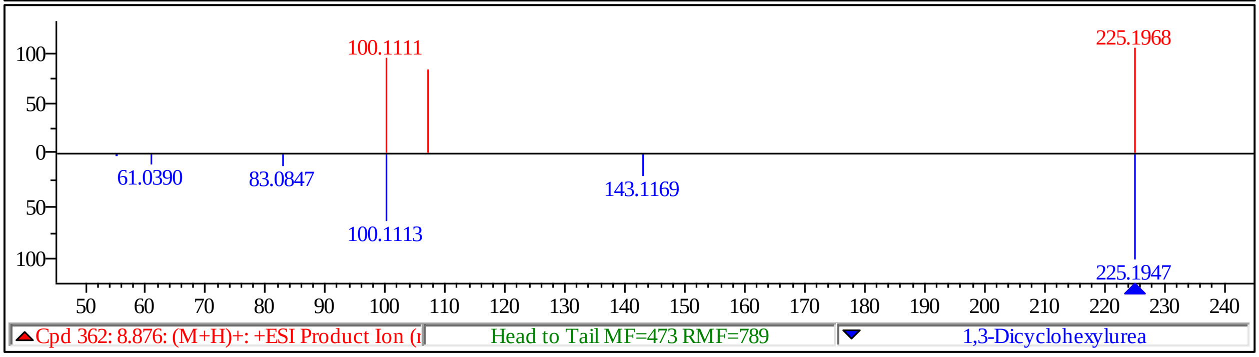
100.1111
225.1968
61.0390
83.0847
143.1169
100.1113
225.1947
Cpd 362: 8.876: (M+H)+: +ESI Product Ion (r
Head to Tail MF=473 RMF=789
1,3-Dicyclohexylurea

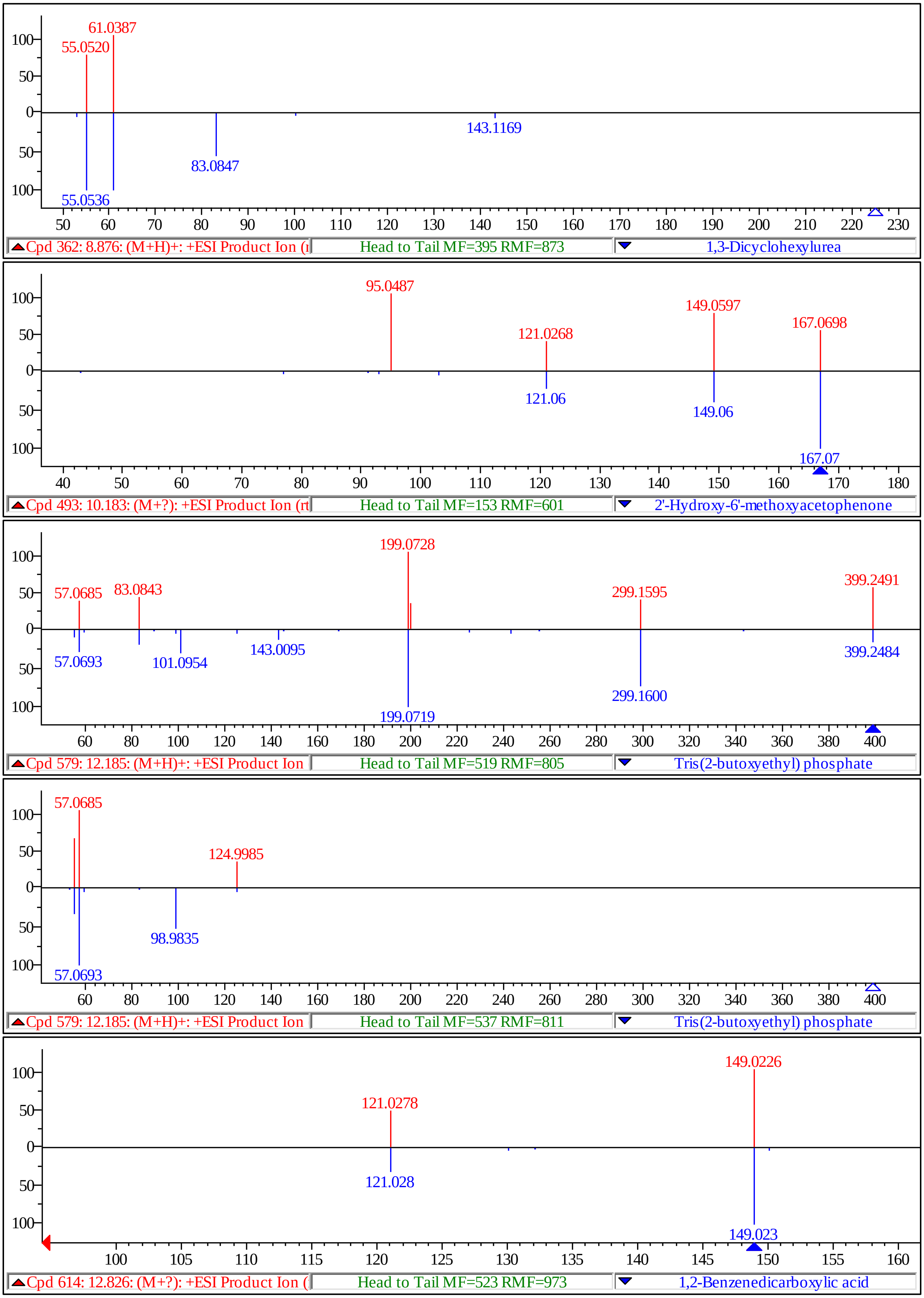

EBC from non-smokers with the common cold

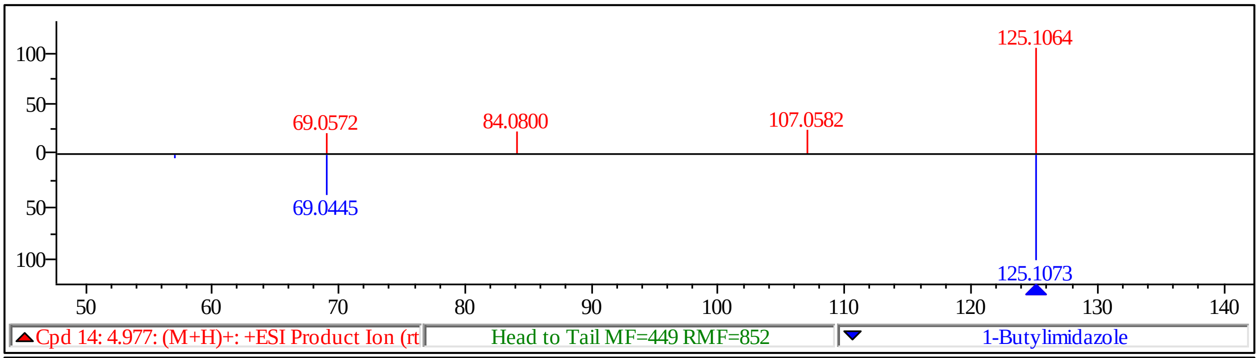
125.1064
69.0572
84.0800
107.0582
69.0445
125.1073
Cpd 14: 4.977: (M+H)+: +ESI Product Ion (rt
Head to Tail MF=449 RMF=852
1-Butylimidazole

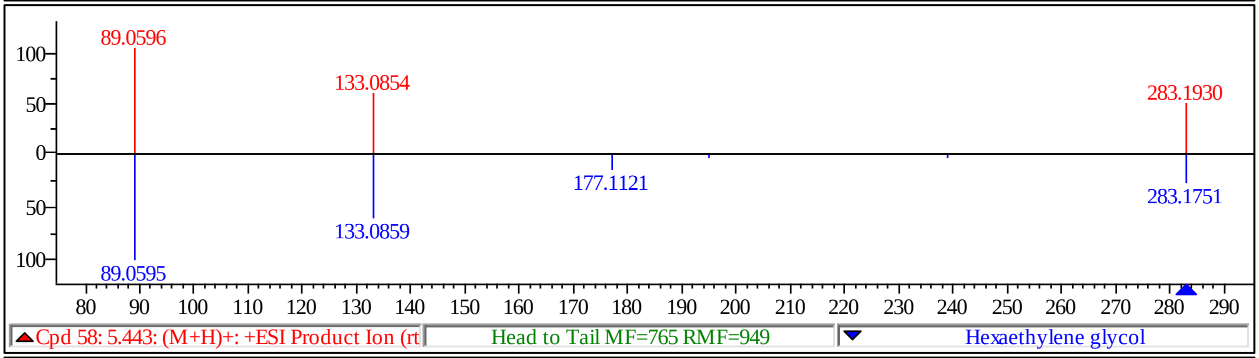
89.0596
133.0854
283.1930
177.1121
283.1751
133.0859
89.0595
Cpd 58: 5.443: (M+H)+: +ESI Product Ion (rt
Head to Tail MF=765 RMF=949
Hexaethylene glycol

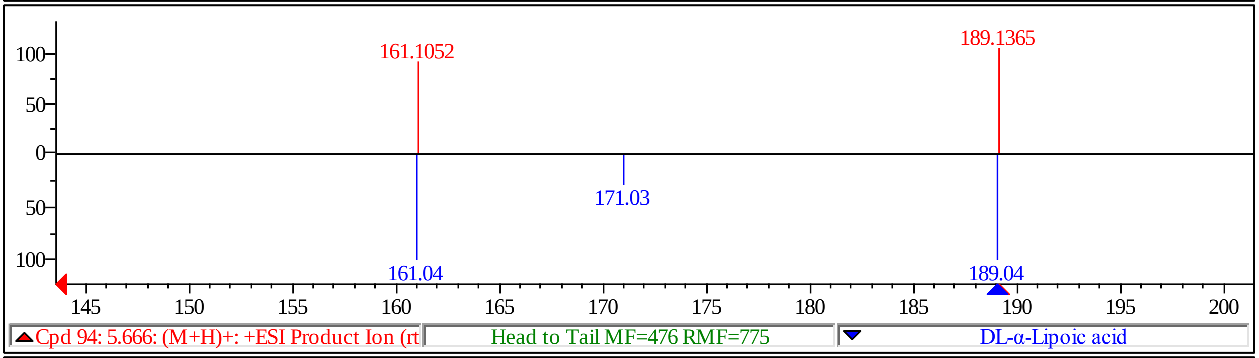
161.1052
189.1365
171.03
161.04
189.04
Cpd 94: 5.666: (M+H)+: +ESI Product Ion (rt
Head to Tail MF=476 RMF=775
DL-α-Lipoic acid

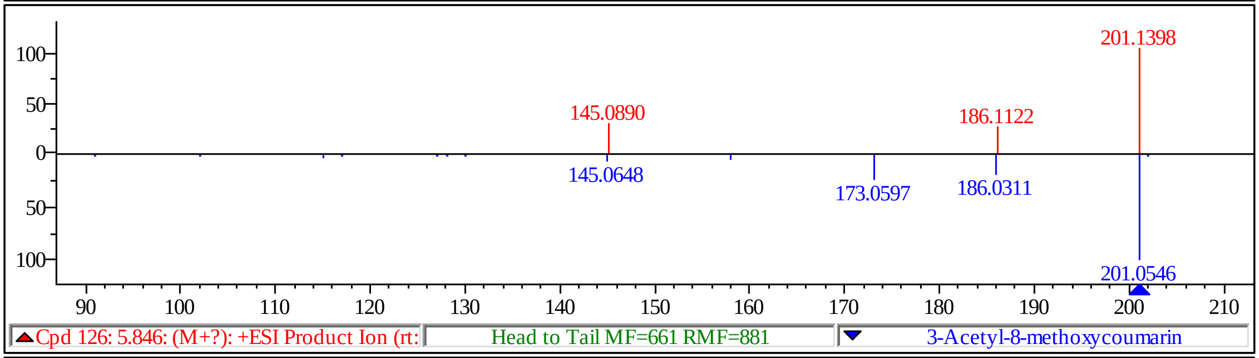
201.1398
145.0890
186.1122
145.0648
173.0597
186.0311
201.0546
Cpd 126: 5.846: (M+?): +ESI Product Ion (rt:
Head to Tail MF=661 RMF=881
3-Acetyl-8-methoxycoumarin

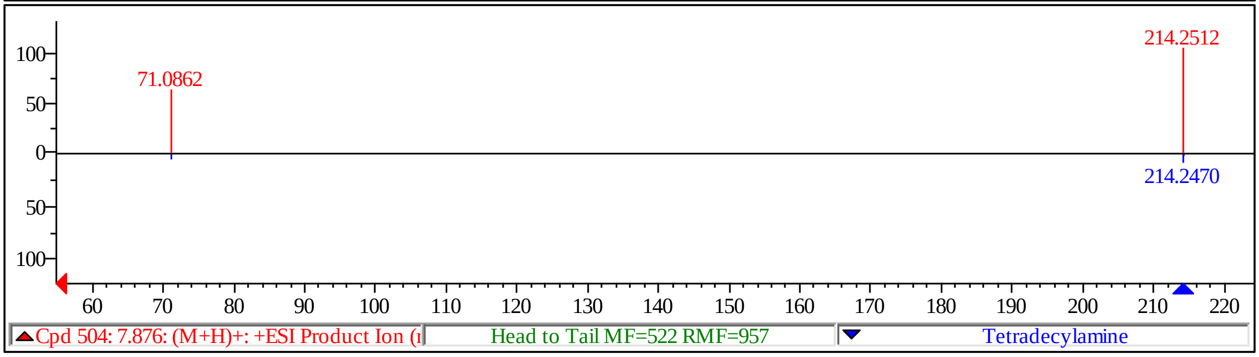
214.2512
71.0862
214.2470
Cpd 504: 7.876: (M+H)+: +ESI Product Ion (r
Head to Tail MF=522 RMF=957
Tetradecylamine

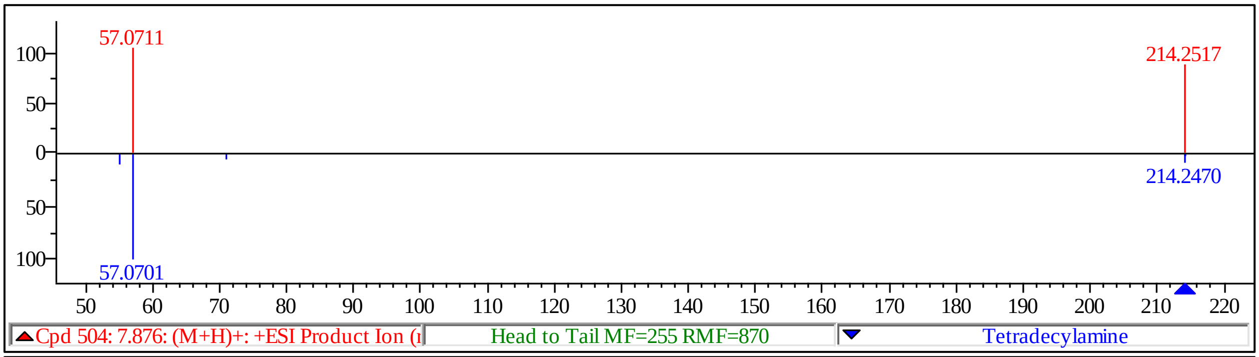
57.0711
214.2517
57.0701
214.2470
Cpd 504: 7.876: (M+H)+: +ESI Product Ion (
Head to Tail MF=255 RMF=870
Tetradecylamine

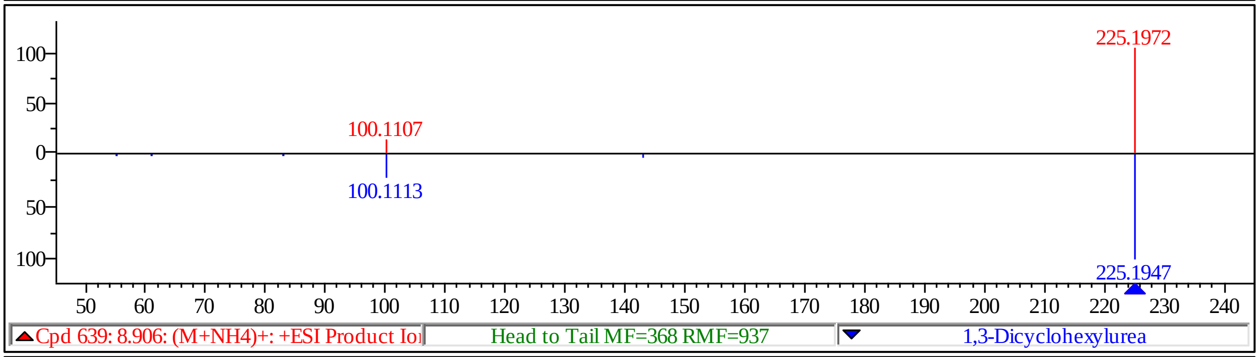
225.1972
100.1107
100.1113
225.1947
Cpd 639: 8.906: (M+NH4)+: +ESI Product Io
Head to Tail MF=368 RMF=937
1,3-Dicyclohexylurea

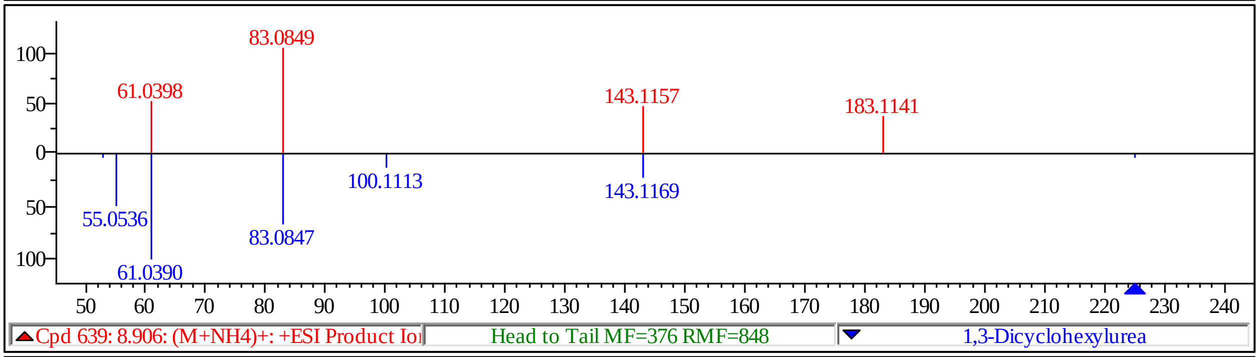
83.0849
61.0398
143.1157
183.1141
100.1113
143.1169
55.0536
83.0847
61.0390
Cpd 639: 8.906: (M+NH4)+: +ESI Product Io
Head to Tail MF=376 RMF=848
1,3-Dicyclohexylurea

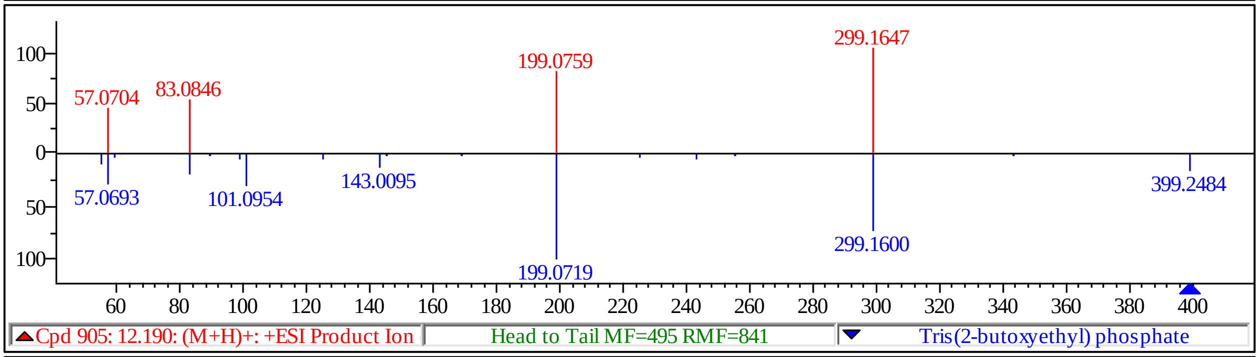
299.1647
199.0759
57.0704
83.0846
57.0693
101.0954
143.0095
399.2484
299.1600
199.0719
Cpd 905: 12.190: (M+H)+: +ESI Product Ion
Head to Tail MF=495 RMF=841
Tris(2-butoxyethyl) phosphate

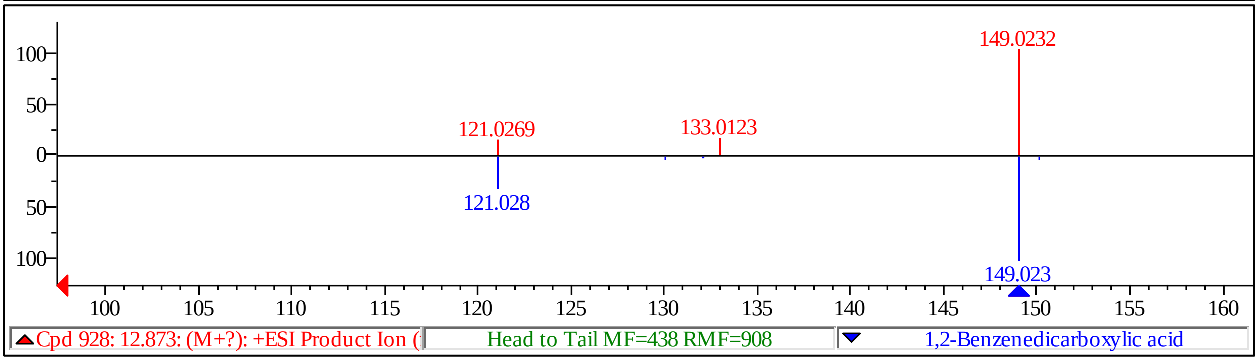
149.0232
121.0269
133.0123
121.028
149.023
Cpd 928: 12.873: (M+?): +ESI Product Ion (
Head to Tail MF=438 RMF=908
1,2-Benzenedicarboxylic acid

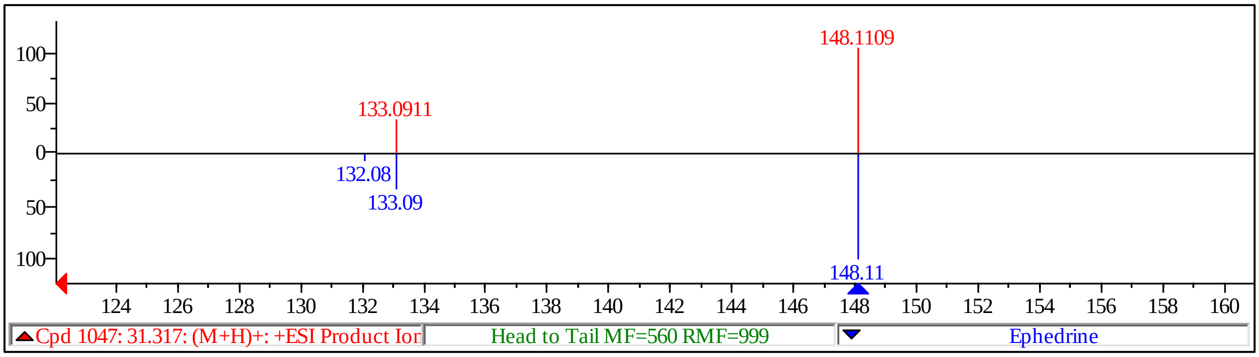

EBC from non-smokers with nasal congestion

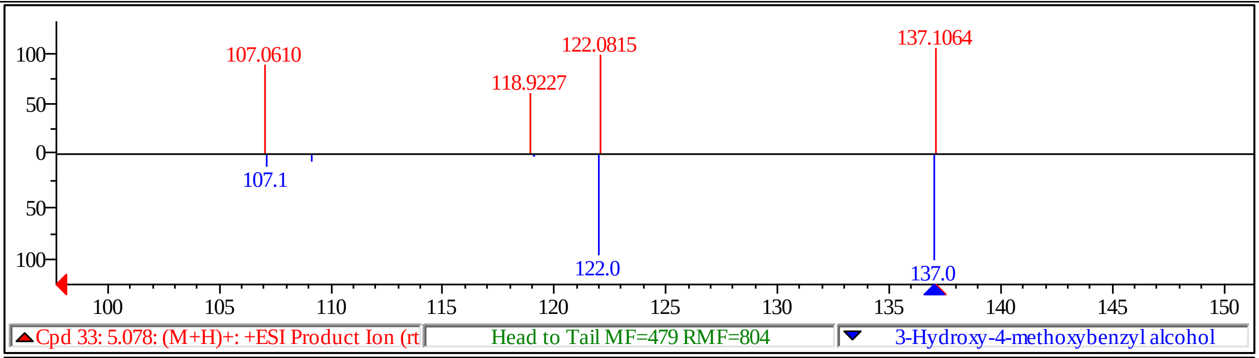

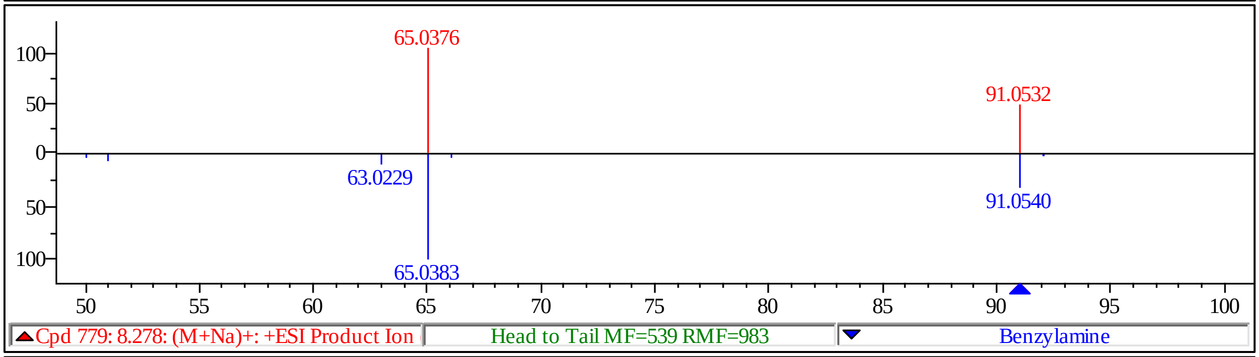

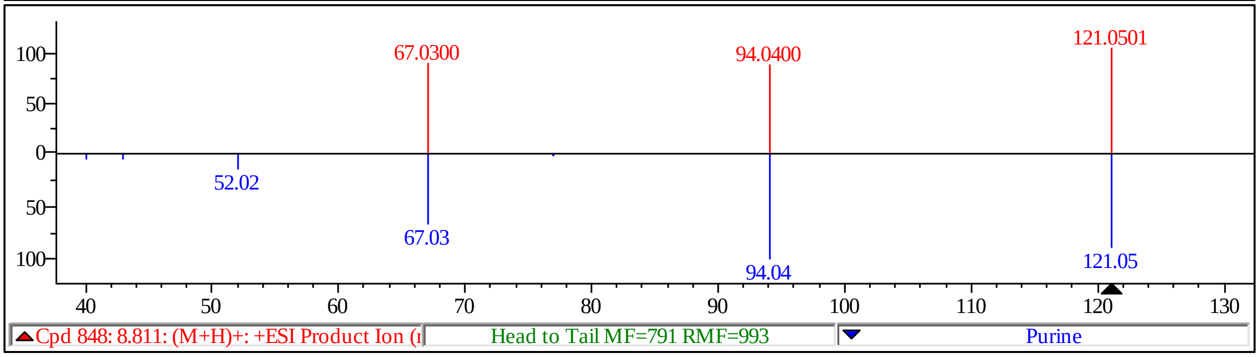

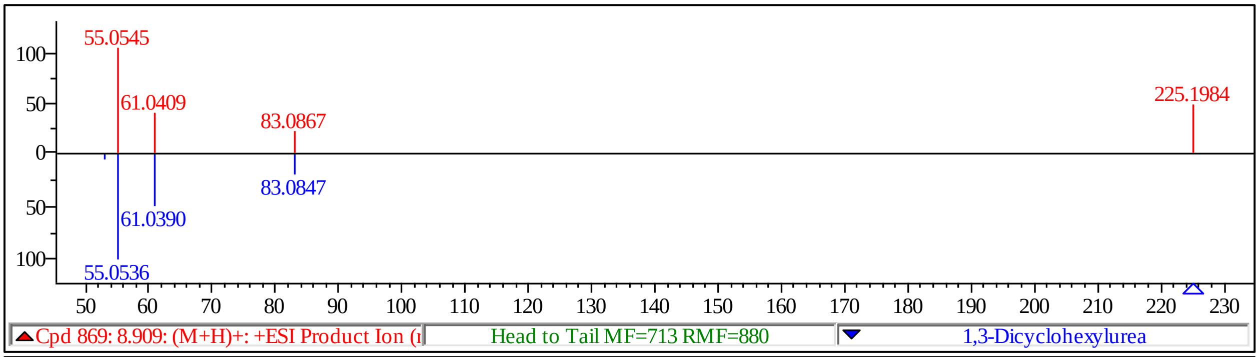

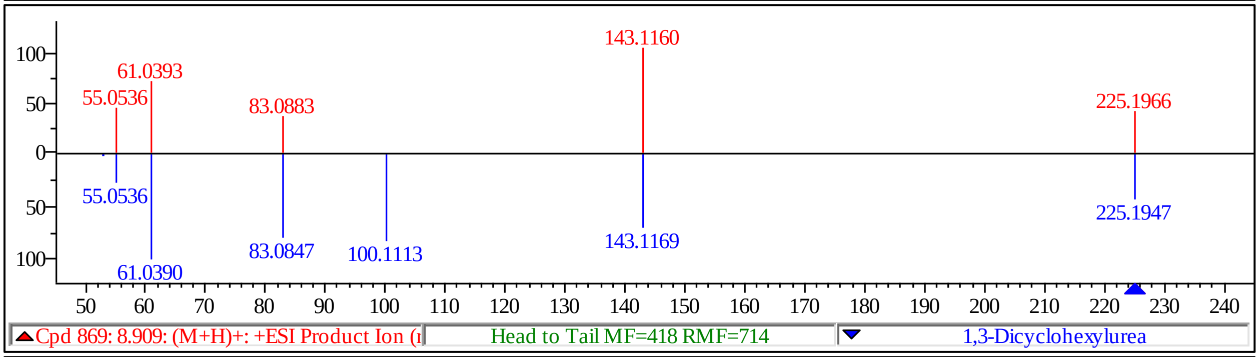

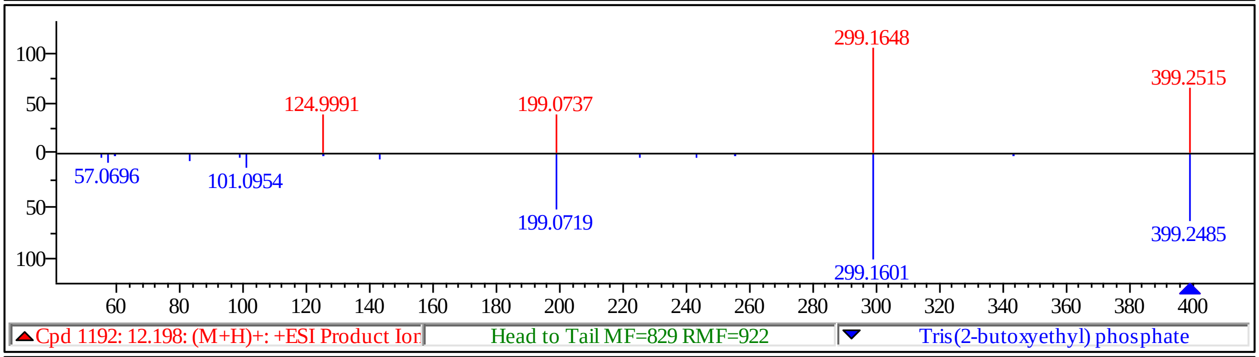

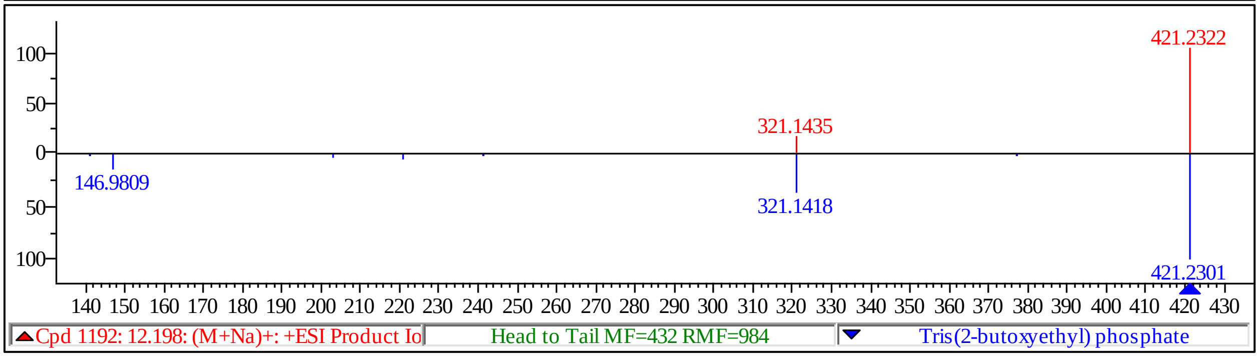

EBC from healthy non-smokers

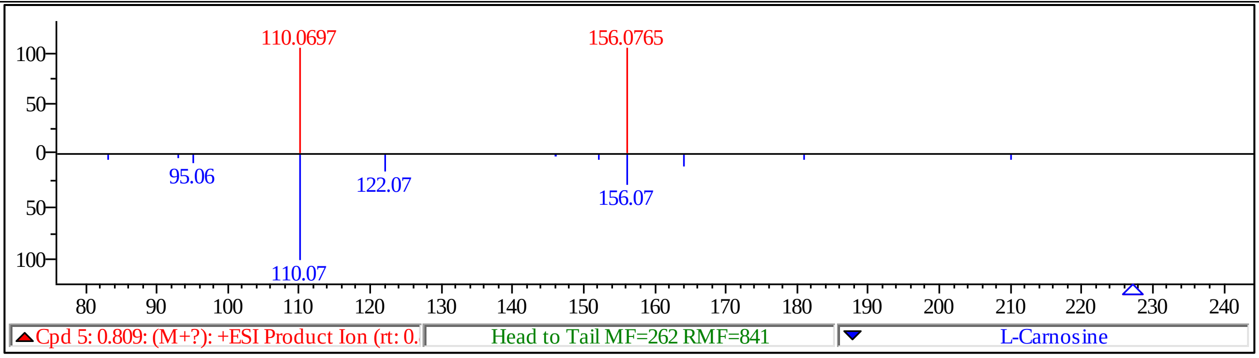

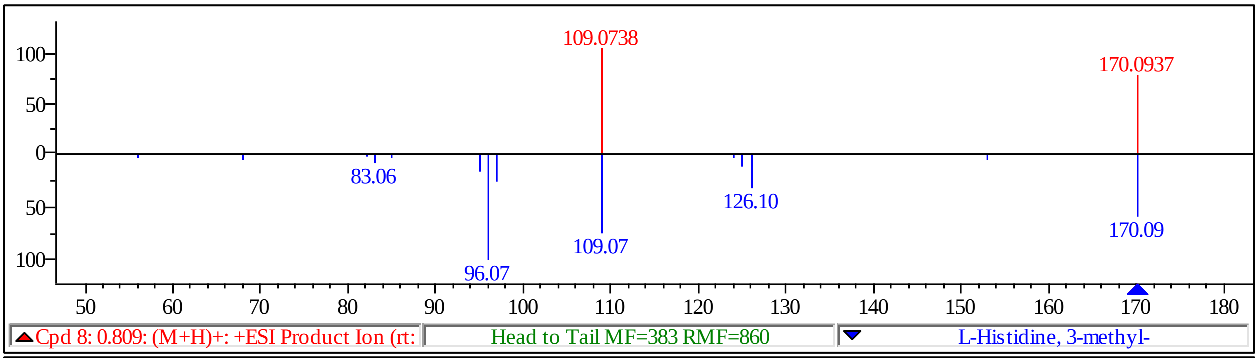
109.0738
170.0937
83.06
96.07
109.07
126.10
170.09
Cpd 8: 0.809: (M+H)+: +ESI Product Ion (rt:
Head to Tail MF=383 RMF=860
L-Histidine, 3-methyl-

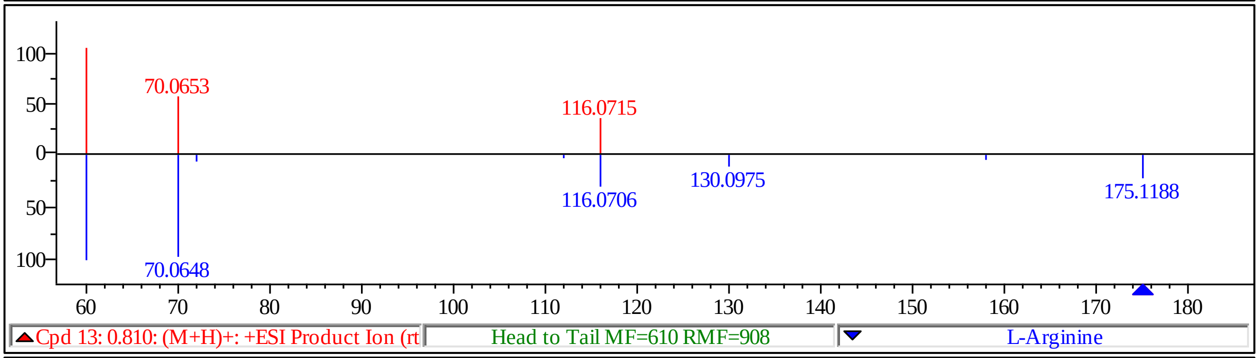
70.0653
116.0715
116.0706
130.0975
175.1188
70.0648
Cpd 13: 0.810: (M+H)+: +ESI Product Ion (rt
Head to Tail MF=610 RMF=908
L-Arginine

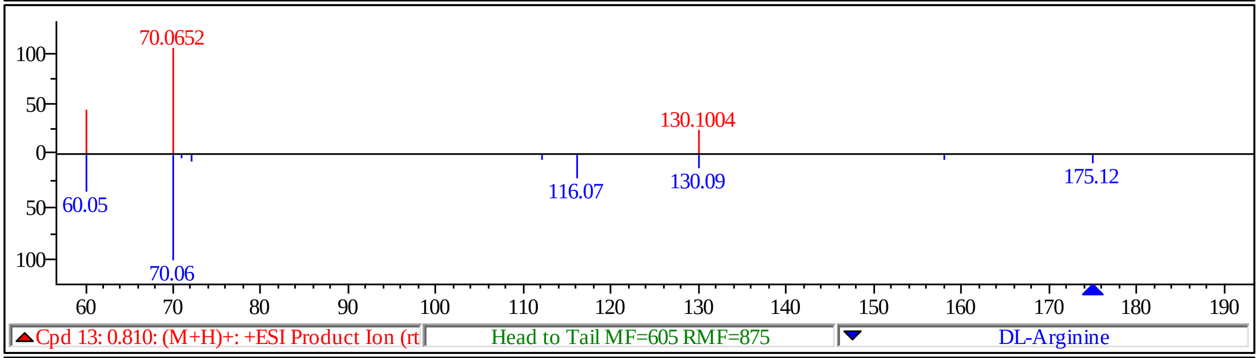
70.0652
130.1004
60.05
116.07
130.09
175.12
70.06
Cpd 13: 0.810: (M+H)+: +ESI Product Ion (rt
Head to Tail MF=605 RMF=875
DL-Arginine

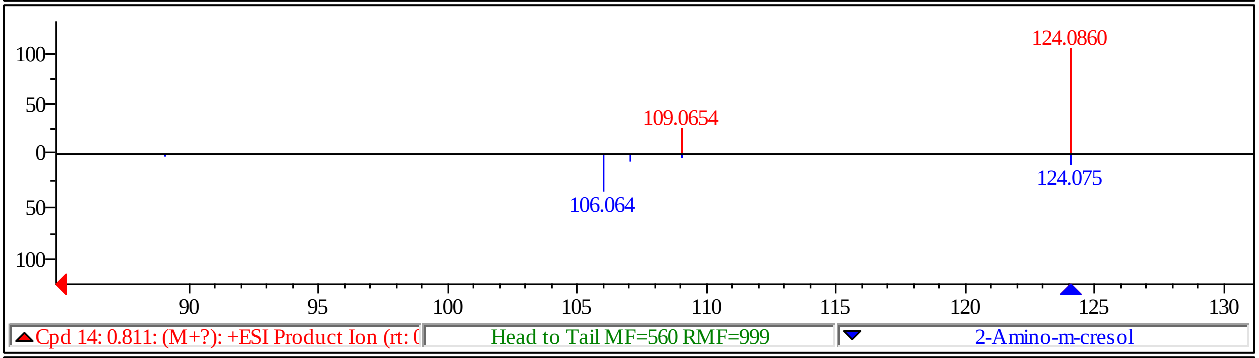
124.0860
109.0654
106.064
124.075
Cpd 14: 0.811: (M+?): +ESI Product Ion (rt: (
Head to Tail MF=560 RMF=999
2-Amino-m-cresol

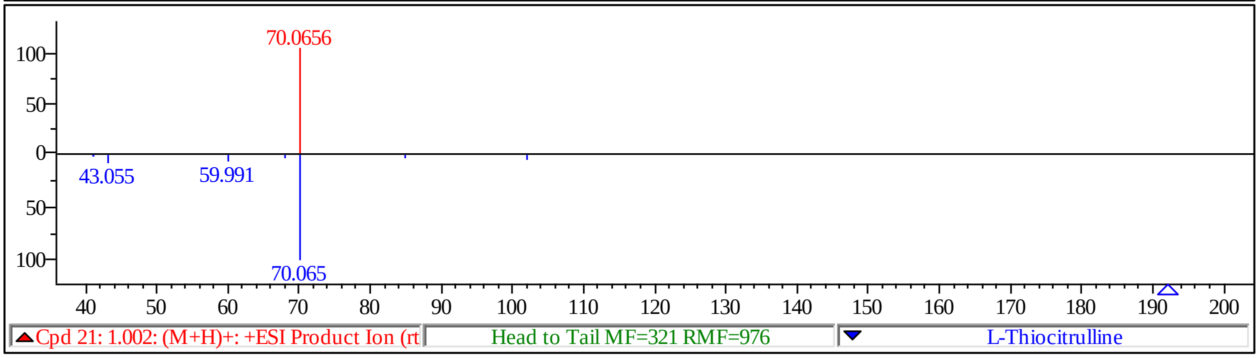
70.0656
43.055
59.991
70.065
Cpd 21: 1.002: (M+H)+: +ESI Product Ion (rt
Head to Tail MF=321 RMF=976
L-Thiocitrulline

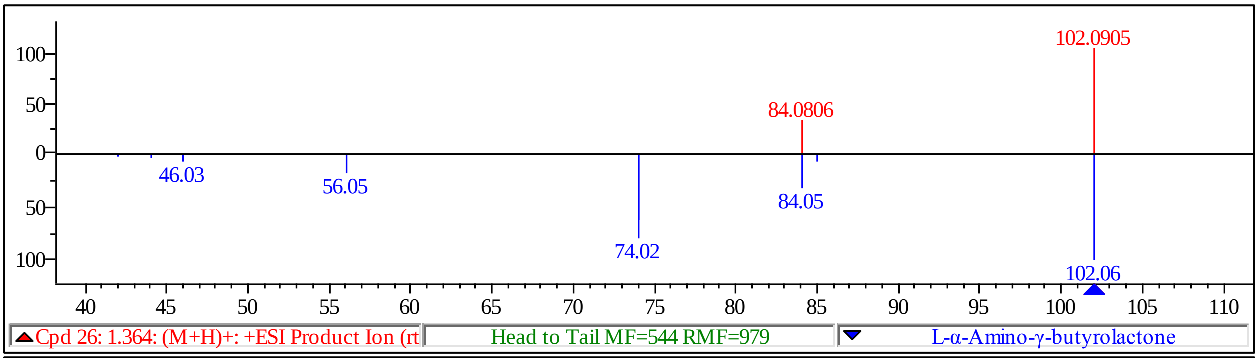
102.0905
84.0806
46.03
56.05
74.02
84.05
102.06
Cpd 26: 1.364: (M+H)+: +ESI Product Ion (rt
Head to Tail MF=544 RMF=979
L-α-Amino-γ-butyrolactone

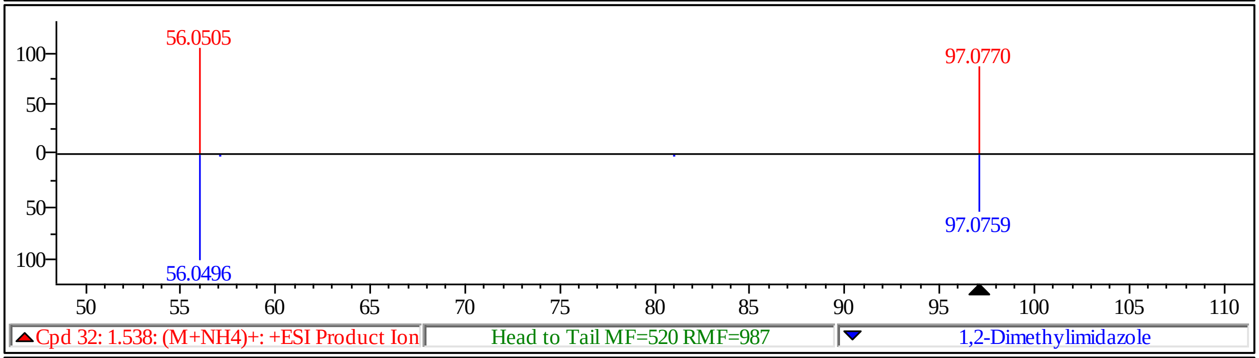
56.0505
97.0770
97.0759
56.0496
Cpd 32: 1.538: (M+NH4)+: +ESI Product Ion
Head to Tail MF=520 RMF=987
1,2-Dimethylimidazole

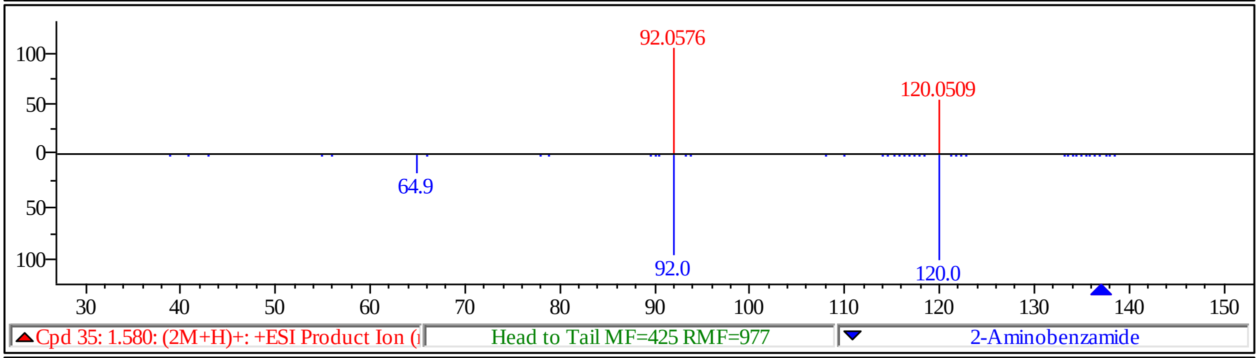
92.0576
120.0509
64.9
92.0
120.0
Cpd 35: 1.580: (2M+H)+: +ESI Product Ion (i
Head to Tail MF=425 RMF=977
2-Aminobenzamide

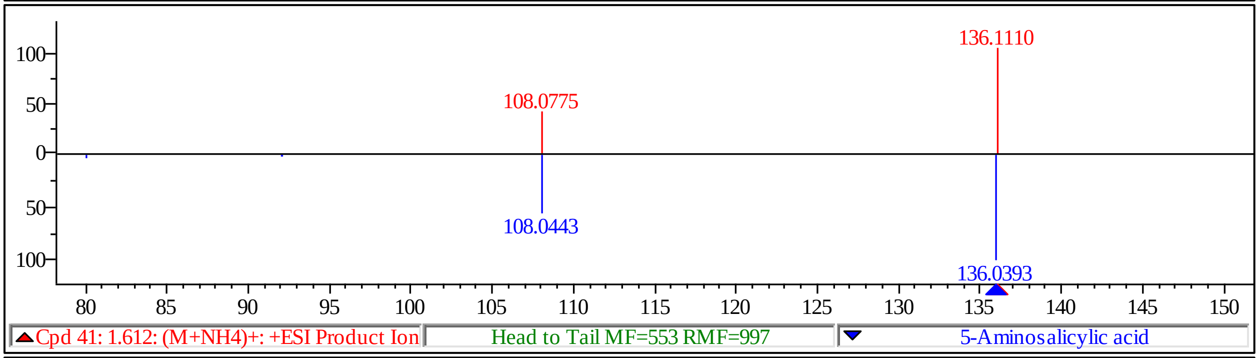
136.1110
108.0775
108.0443
136.0393
Cpd 41: 1.612: (M+NH4)+: +ESI Product Ion
Head to Tail MF=553 RMF=997
5-Aminosalicylic acid

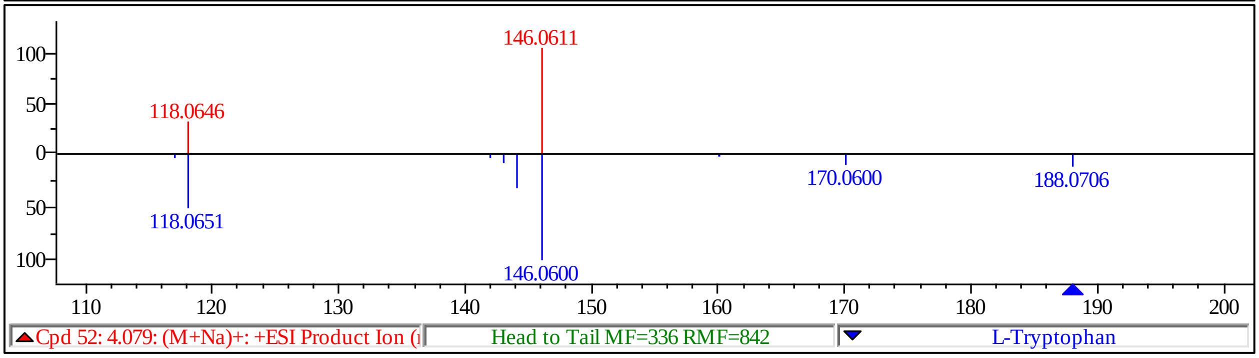
146.0611
118.0646
118.0651
170.0600
188.0706
146.0600
Cpd 52: 4.079: (M+Na)+: +ESI Product Ion (i
Head to Tail MF=336 RMF=842
L-Tryptophan

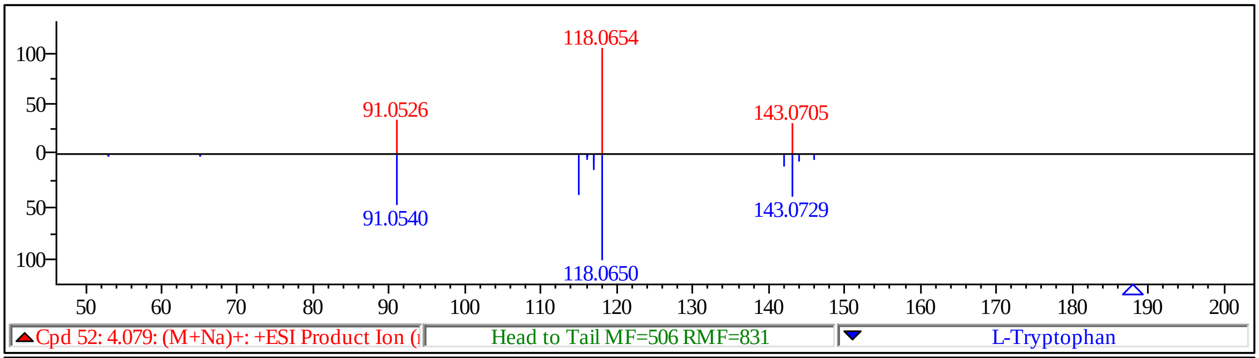
118.0654
91.0526
143.0705
91.0540
143.0729
118.0650
Cpd 52: 4.079: (M+Na)+: +ESI Product Ion (
Head to Tail MF=506 RMF=831
L-Tryptophan

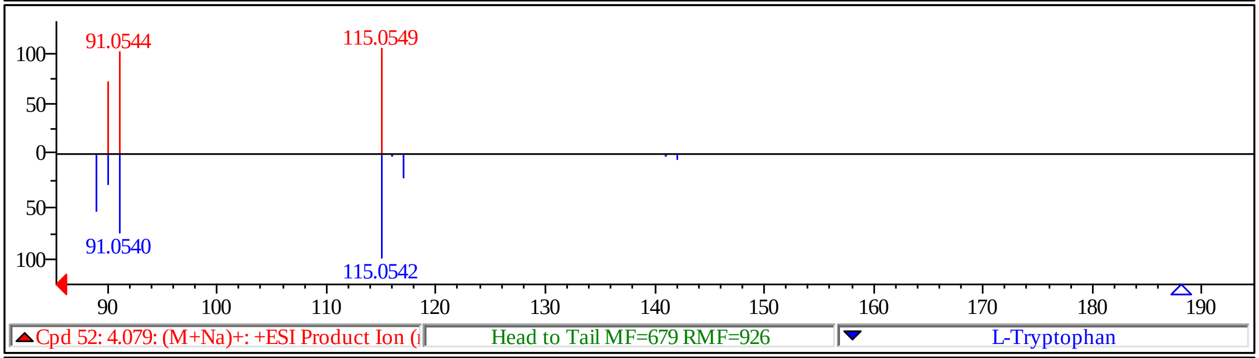
91.0544
115.0549
91.0540
115.0542
Cpd 52: 4.079: (M+Na)+: +ESI Product Ion (
Head to Tail MF=679 RMF=926
L-Tryptophan

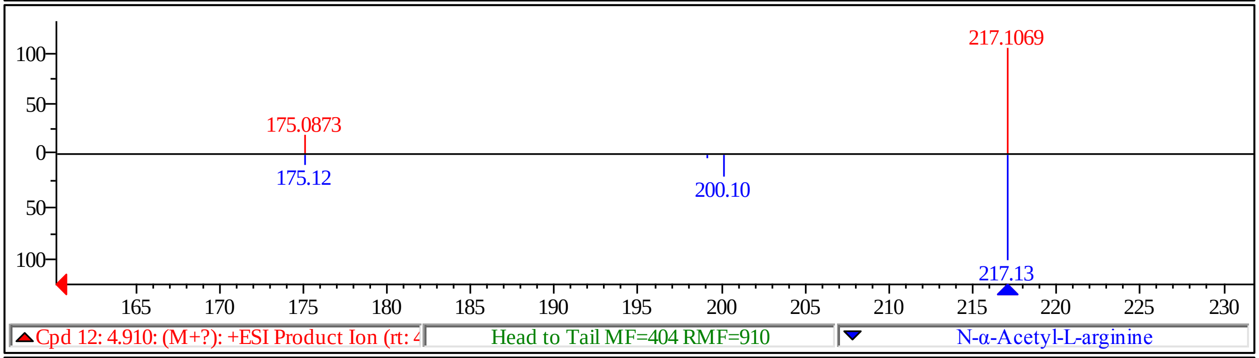
217.1069
175.0873
175.12
200.10
217.13
Cpd 12: 4.910: (M+?): +ESI Product Ion (rt:
Head to Tail MF=404 RMF=910
N-α-Acetyl-L-arginine

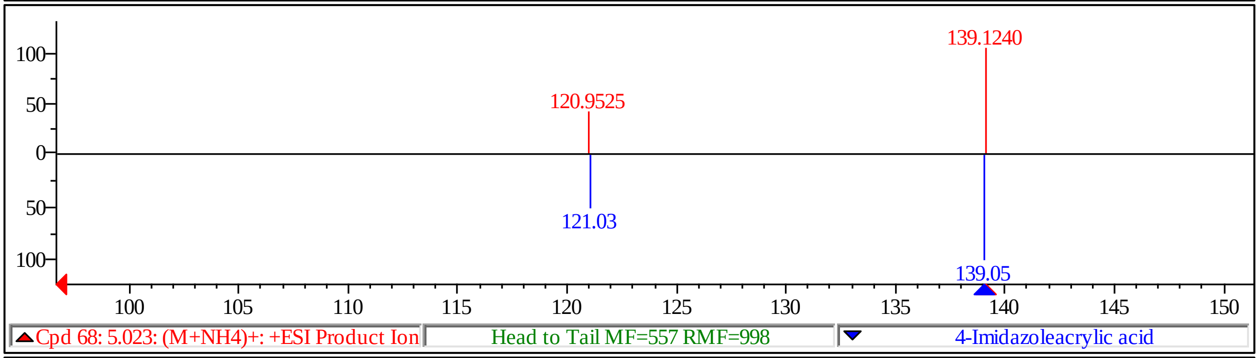
139.1240
120.9525
121.03
139.05
Cpd 68: 5.023: (M+NH4)+: +ESI Product Ion
Head to Tail MF=557 RMF=998
4-Imidazoleacrylic acid

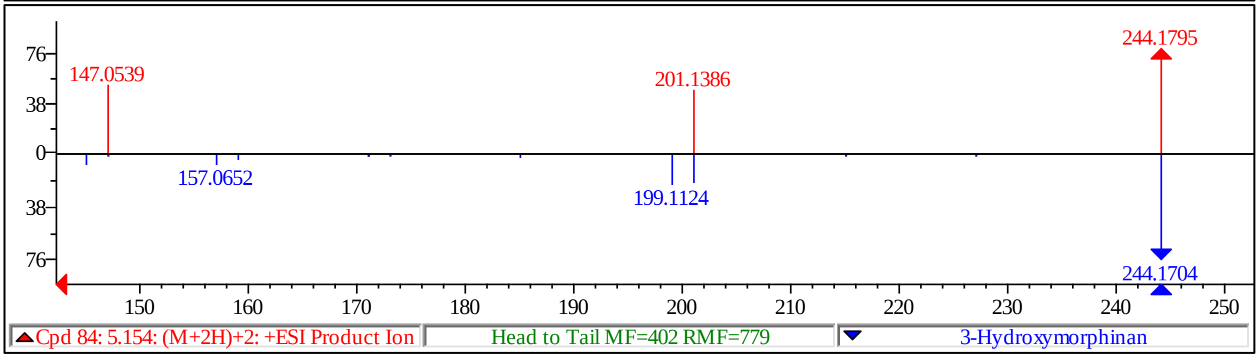
244.1795
147.0539
201.1386
157.0652
199.1124
244.1704
Cpd 84: 5.154: (M+2H)+2: +ESI Product Ion
Head to Tail MF=402 RMF=779
3-Hydroxymorphinan

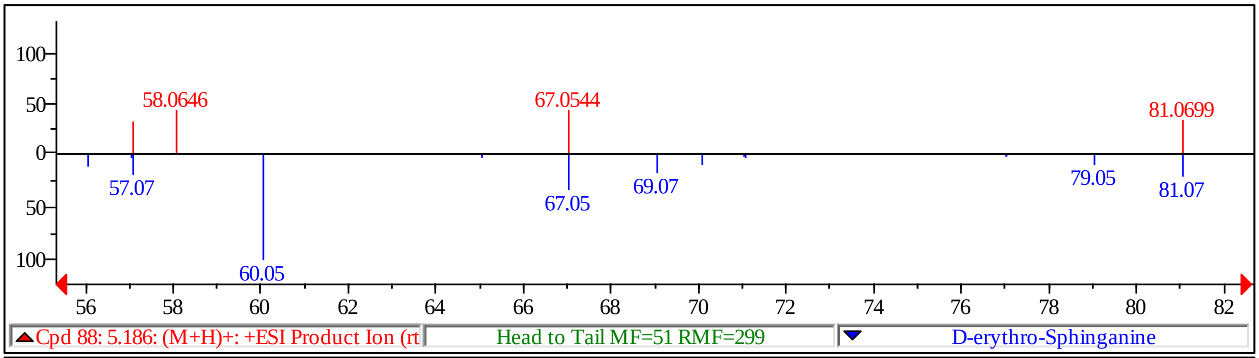
58.0646
67.0544
81.0699
57.07
67.05
69.07
79.05
81.07
60.05
Cpd 88: 5.186: (M+H)+: +ESI Product Ion (rt
Head to Tail MF=51 RMF=299
D-erythro-Sphinganine

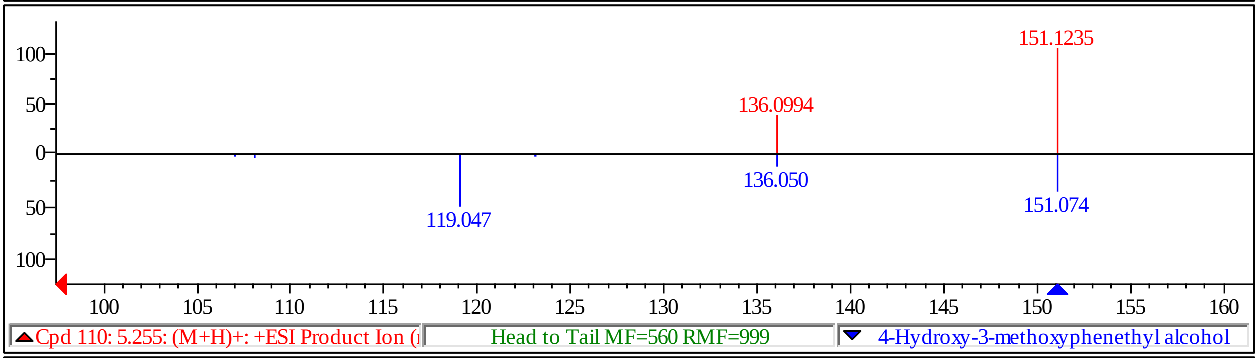
151.1235
136.0994
136.050
151.074
119.047
Cpd 110: 5.255: (M+H)+: +ESI Product Ion (i
Head to Tail MF=560 RMF=999
4-Hydroxy-3-methoxyphenethyl alcohol

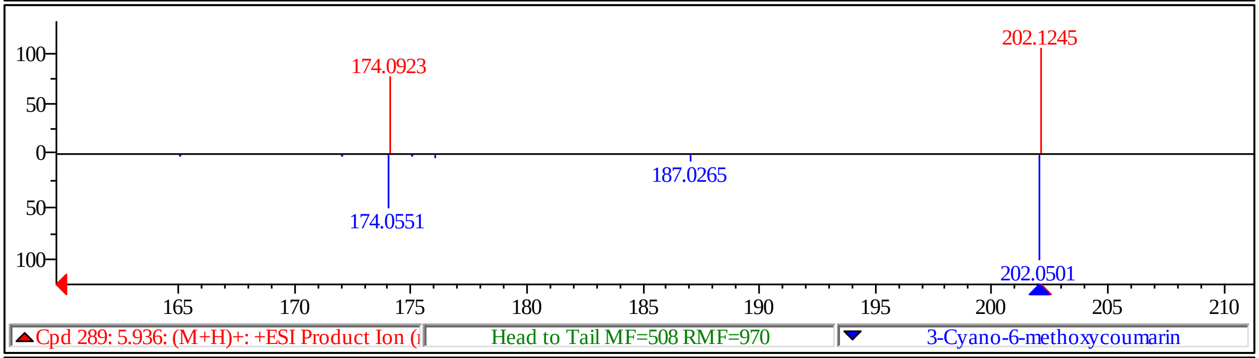
202.1245
174.0923
187.0265
174.0551
202.0501
Cpd 289: 5.936: (M+H)+: +ESI Product Ion (i
Head to Tail MF=508 RMF=970
3-Cyano-6-methoxycoumarin

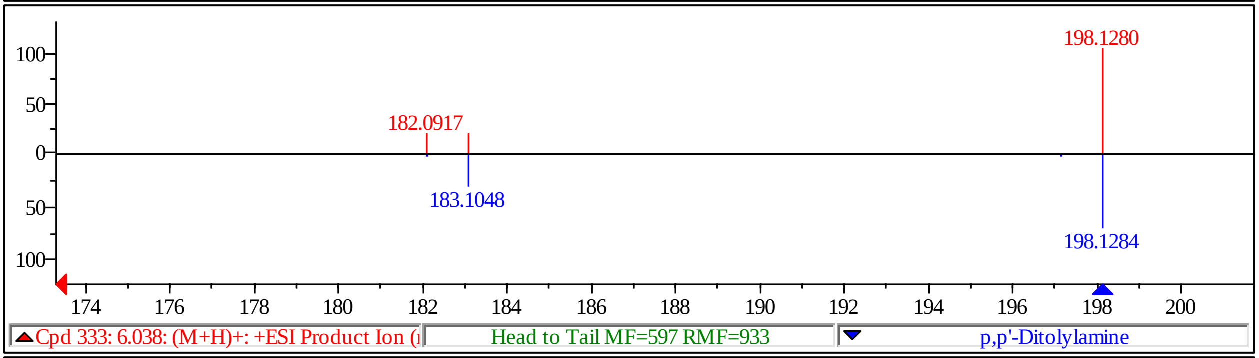
198.1280
182.0917
183.1048
198.1284
Cpd 333: 6.038: (M+H)+: +ESI Product Ion (i
Head to Tail MF=597 RMF=933
p,p'-Ditolylamine

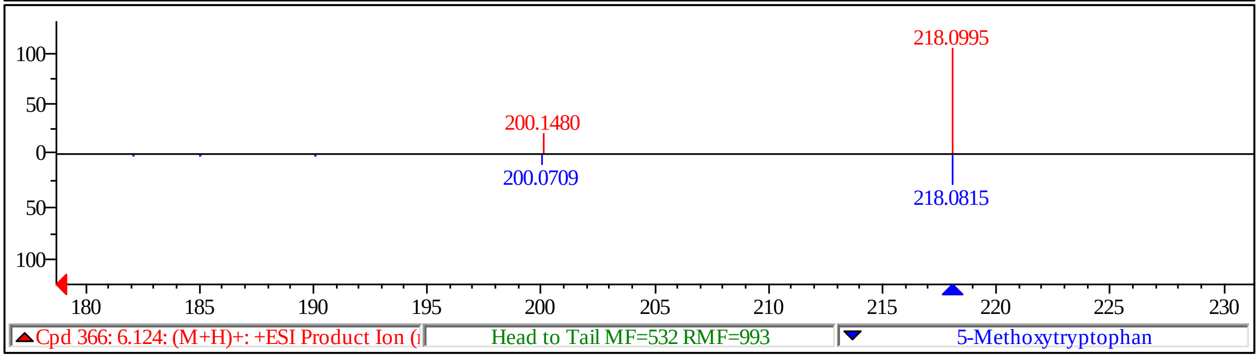
218.0995
200.1480
200.0709
218.0815
Cpd 366: 6.124: (M+H)+: +ESI Product Ion (i
Head to Tail MF=532 RMF=993
5-Methoxytryptophan

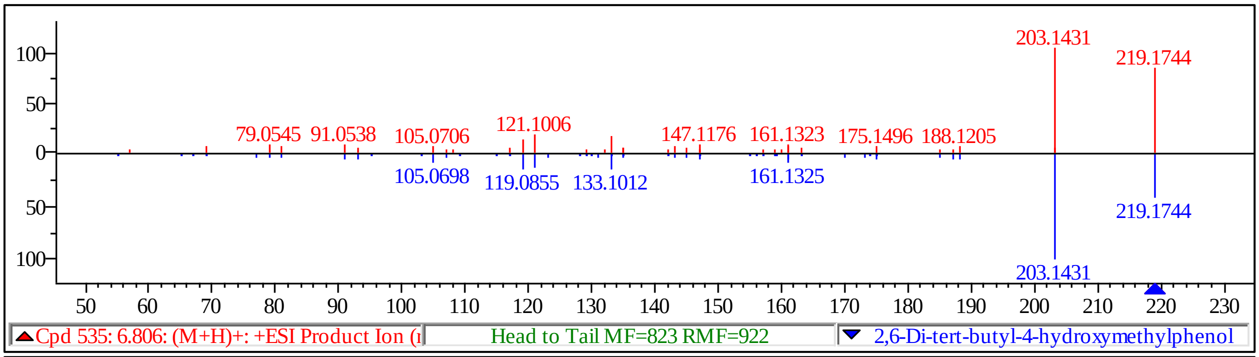
203.1431
219.1744
79.0545 91.0538 105.0706 121.1006 147.1176 161.1323 175.1496 188.1205
105.0698 119.0855 133.1012 161.1325
219.1744
203.1431
Cpd 535: 6.806: (M+H)+: +ESI Product Ion (i
Head to Tail MF=823 RMF=922
2,6-Di-tert-butyl-4-hydroxymethylphenol

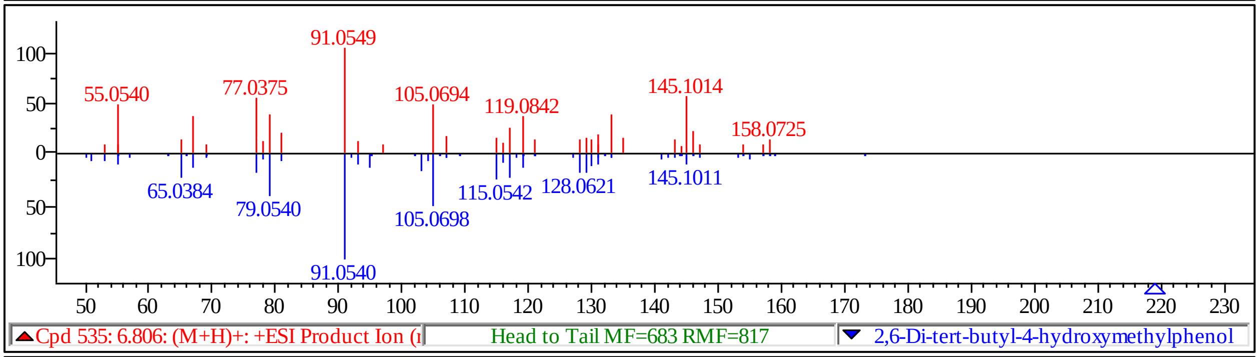
91.0549
55.0540 77.0375 105.0694 119.0842 145.1014 158.0725
65.0384 79.0540 115.0542 128.0621 145.1011
105.0698
91.0540
Cpd 535: 6.806: (M+H)+: +ESI Product Ion (i
Head to Tail MF=683 RMF=817
2,6-Di-tert-butyl-4-hydroxymethylphenol

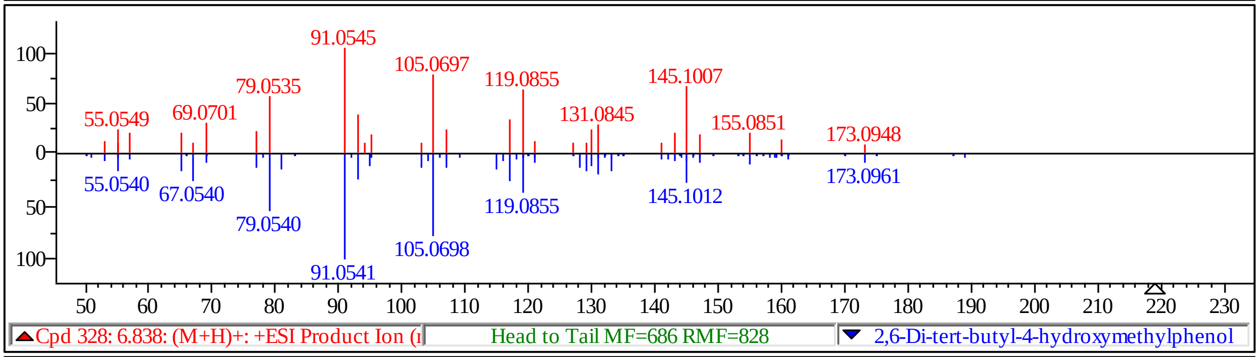
91.0545
105.0697 119.0855 145.1007
79.0535
55.0549 69.0701 131.0845 155.0851 173.0948
55.0540 67.0540 173.0961
79.0540 119.0855 145.1012
105.0698
91.0541
Cpd 328: 6.838: (M+H)+: +ESI Product Ion (i
Head to Tail MF=686 RMF=828
2,6-Di-tert-butyl-4-hydroxymethylphenol

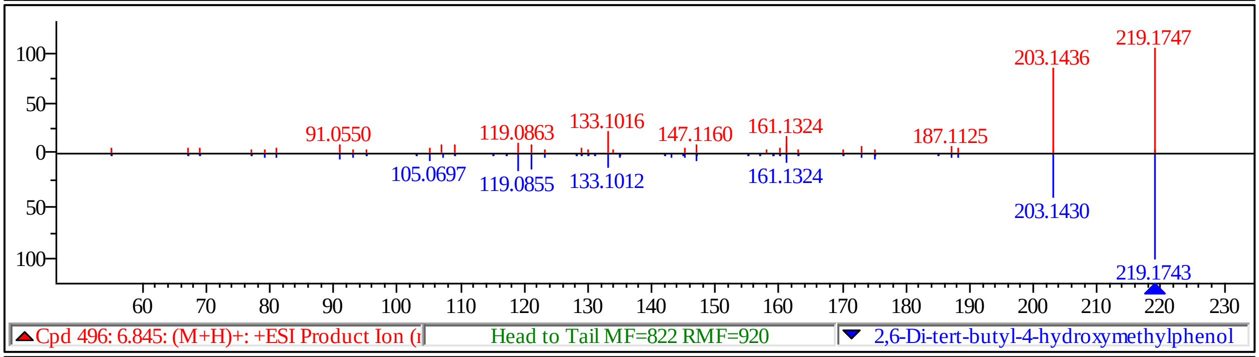
219.1747
203.1436
91.0550 119.0863 133.1016 147.1160 161.1324 187.1125
105.0697 119.0855 133.1012 161.1324
203.1430
219.1743
Cpd 496: 6.845: (M+H)+: +ESI Product Ion (i
Head to Tail MF=822 RMF=920
2,6-Di-tert-butyl-4-hydroxymethylphenol

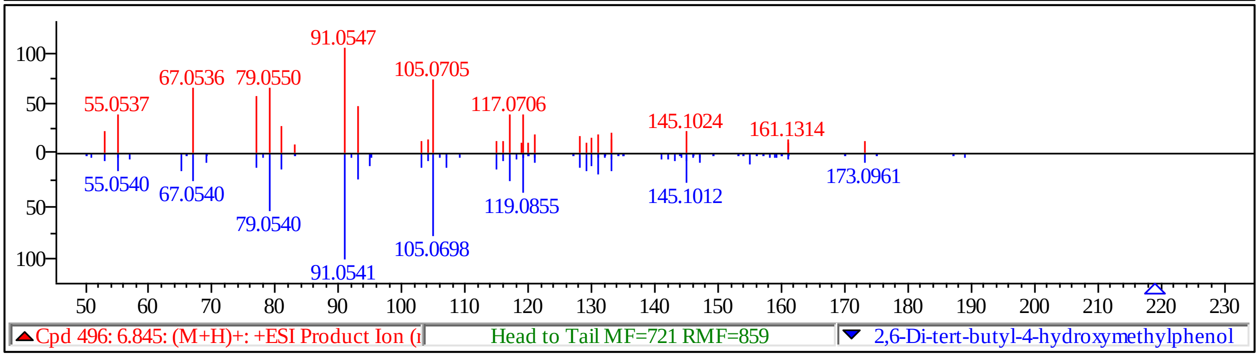
91.0547
105.0705
67.0536 79.0550
55.0537 117.0706
145.1024 161.1314
55.0540 67.0540 173.0961
79.0540 119.0855 145.1012
105.0698
91.0541
Cpd 496: 6.845: (M+H)+: +ESI Product Ion (i
Head to Tail MF=721 RMF=859
2,6-Di-tert-butyl-4-hydroxymethylphenol

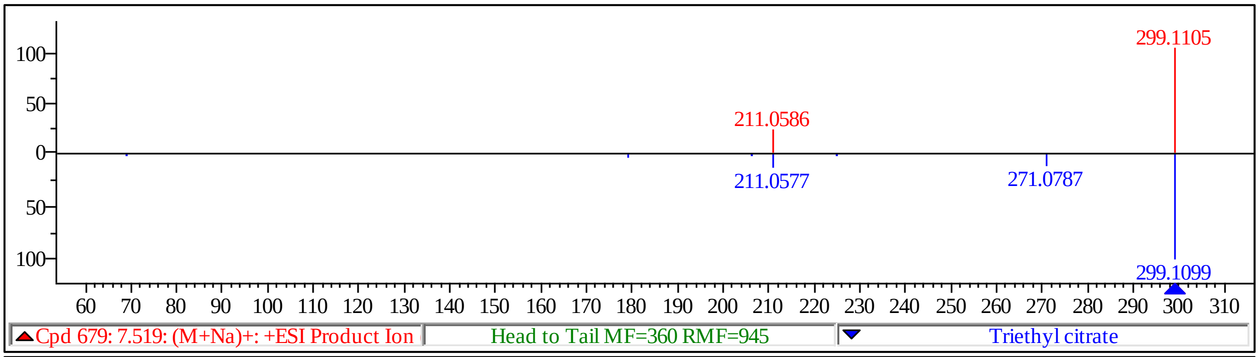
299.1105
211.0586
211.0577
271.0787
299.1099
Cpd 679: 7.519: (M+Na)+: +ESI Product Ion
Head to Tail MF=360 RMF=945
Triethyl citrate

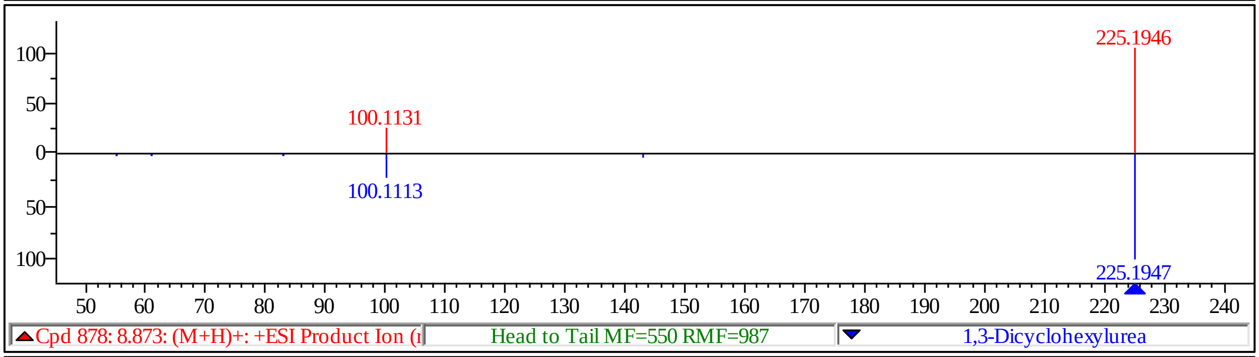
225.1946
100.1131
100.1113
225.1947
Cpd 878: 8.873: (M+H)+: +ESI Product Ion (
Head to Tail MF=550 RMF=987
1,3-Dicyclohexylurea

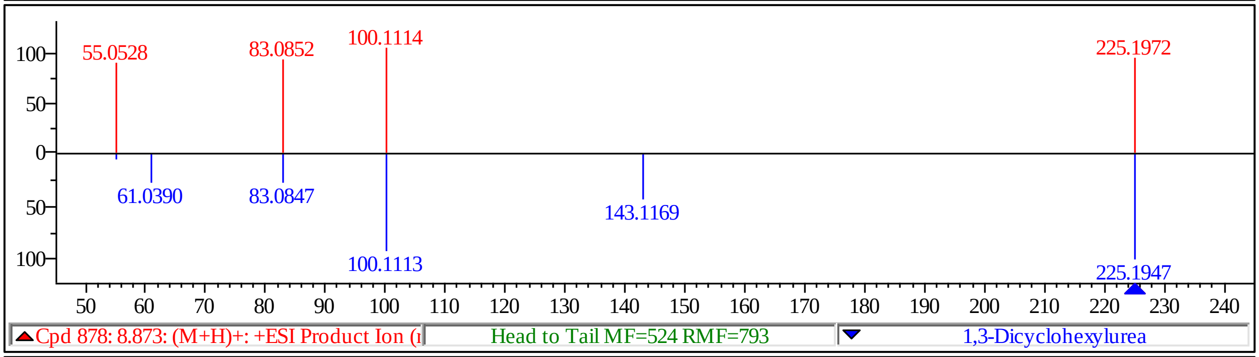
55.0528
83.0852
100.1114
225.1972
61.0390
83.0847
143.1169
100.1113
225.1947
Cpd 878: 8.873: (M+H)+: +ESI Product Ion (
Head to Tail MF=524 RMF=793
1,3-Dicyclohexylurea

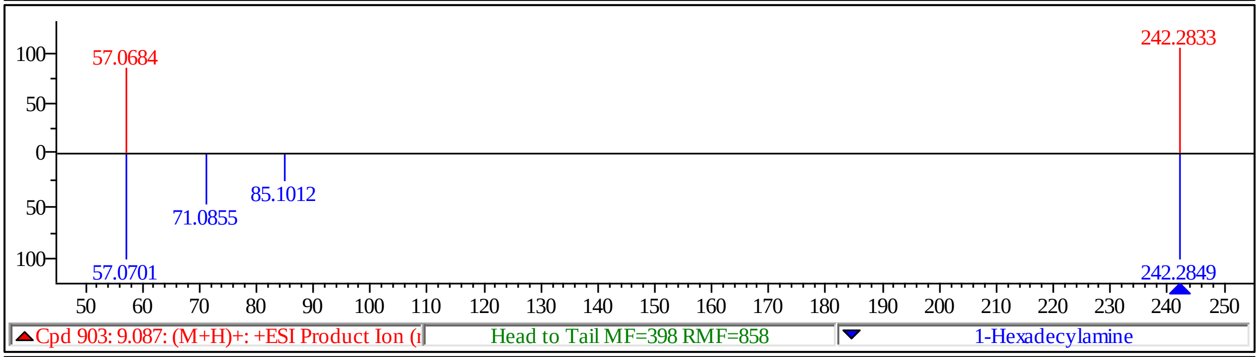
57.0684
242.2833
85.1012
71.0855
57.0701
242.2849
Cpd 903: 9.087: (M+H)+: +ESI Product Ion (
Head to Tail MF=398 RMF=858
1-Hexadecylamine

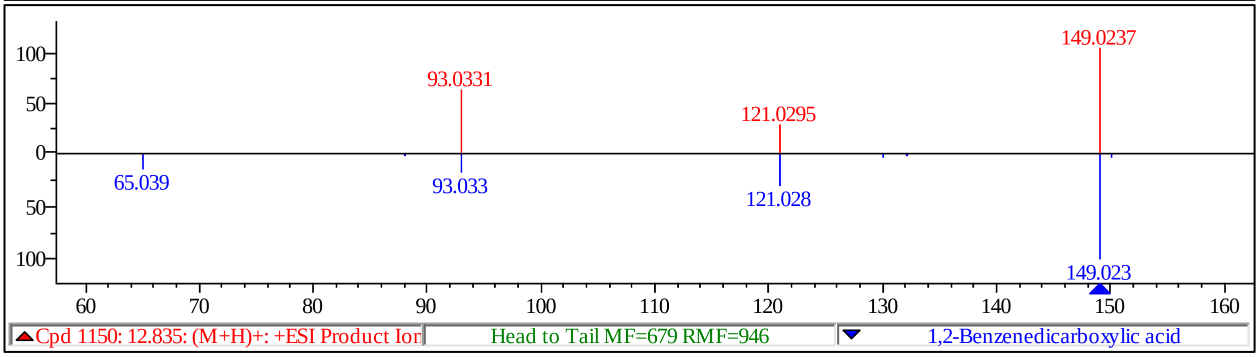
149.0237
93.0331
121.0295
65.039
93.033
121.028
149.023
Cpd 1150: 12.835: (M+H)+: +ESI Product Ior
Head to Tail MF=679 RMF=946
1,2-Benzenedicarboxylic acid

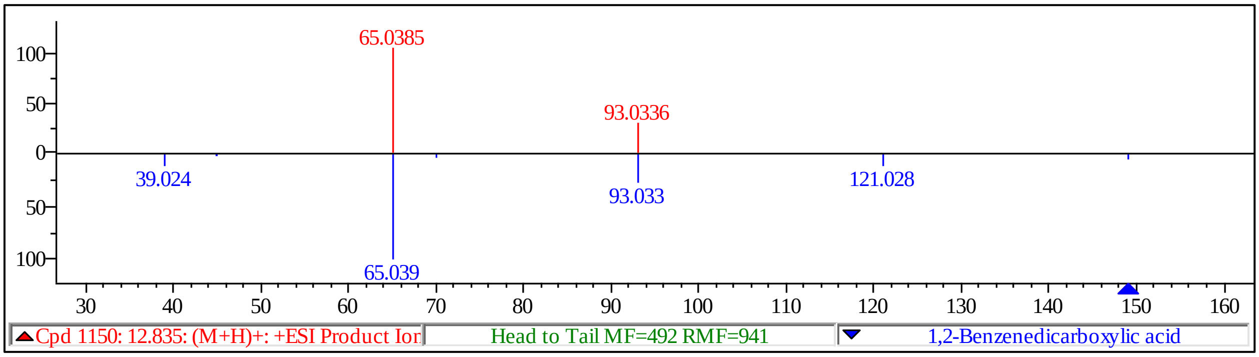
100
50
0
50
100
65.0385
93.0336
39.024
93.033
121.028
65.039
30
40
50
60
70
80
90
100
110
120
130
140
150
160
Cpd 1150: 12.835: (M+H)+: +ESI Product Ior
Head to Tail MF=492 RMF=941
1,2-Benzenedicarboxylic acid
