# Supplementary material for: Determining the presence of asthma-related molecules and salivary contamination in exhaled breath condensate
Source: Respir Res. 2017 Apr 12;18:57. doi: 10.1186/s12931-017-0538-5 (PMC5389118; doi:10.1186/s12931-017-0538-5)
Supplement: Supplementary file 8 — Targeted eicosanoid analysis of EBC. EBC was collected from four groups of volunteers: healthy smokers, healthy non-smokers, non-smokers with nasal congestion, and non-smokers with the common cold. Samples were pooled, lyophilized, reconstituted in LC-MS buffer, and analyzed using targeted LC-MS on a triple quadruple mass spectrometer. (PDF 40 kb) [file 12931_2017_538_MOESM8_ESM.pdf]

Additional file 8: Targeted Eicosanoid Analysis of EBC. EBC was collected from four groups of volunteers: healthy smokers, healthy non-smokers, non-smokers with nasal congestion, and non-smokers with the common cold. Samples were pooled, lyophilized, reconstituted in LC-MS buffer, and analyzed using targeted LC-MS on a triple quadrupole mass spectrometer.

Compound Method				Smoker	Nasal Congestion	Common Cold	Healthy
Name	Transition	Associated pathway	Fatty acid	Final Conc (pg/ml)	Final Conc (pg/ml)	Final Conc (pg/ml)	Final Conc (pg/ml)
11(12)-EET	319.2 -> 178.8	CYP450	AA	0.0	0.0	0.0	0.0
11B-PGF2a	353.2 -> 193.1	Cyclooxygenase	AA	**1144.0**	0.0	0.0	0.0
12S-HETE	319.2 -> 178.7	12-Lipoxygenase	AA	0.0	0.0	0.0	0.0
14(15)-EET	319.2 -> 175.0	CYP450	AA	0.0	0.0	0.0	0.0
15R-PGF2a	353.2 -> 309.2	Cyclooxygenase	AA	**2091.5**	0.0	0.0	0.0
15S-HETE	319.2 -> 219.1	15-Lipoxygenase	AA	0.0	0.0	0.0	0.0
5S-HETE	319.2 -> 115.0	5-Lipoxygenase	AA	0.0	0.0	0.0	0.0
6-a-Prostaglandin (PGI2 analog)	353.2 -> 129.0	Cyclooxygenase	AA	0.0	0.0	0.0	0.0
8(9)-EET	319.2 -> 301.1	CYP450	AA	0.0	0.0	0.0	0.0
8-iso-15R-PGF2a	353.2 -> 193.1	Lipid peroxidation/oxidative stress	AA	**2383.2**	0.0	0.0	0.0
8-iso-PGF2a	353.2 -> 193.1	Lipid peroxidation/oxidative stress	AA	**1635.4**	0.0	0.0	0.0
8S-HETE	319.2 -> 154.9	8-hydroxylation	AA	0.0	0.0	0.0	0.0
Carbocyclic Thromboxane A2 (TXA2 analog)	347.3 -> 247.0	Cyclooxygenase	AA	**40.1**	0.0	0.0	0.0
Lipoxin A4 (LXA4)	351.2 -> 114.9	5-Lipoxygenase	AA	**100.8**	0.0	0.0	0.0
LTB4	335.2 -> 195.1	5-Lipoxygenase	AA	**136.4**	0.0	0.0	0.0
LTD4_NEG	495.3 -> 176.9	5-Lipoxygenase/Glutathione-S-transferase	AA	0.0	0.0	0.0	0.0
LTE4_NEG	438.2 -> 333.1	5-Lipoxygenase/Glutathione-S-transferase	AA	**29.9**	0.0	0.0	0.0
PGE2	351.2 -> 315.2	Cyclooxygenase	AA	**963.2**	0.0	0.0	0.0
PGF2a	353.2 -> 309.2	Cyclooxygenase	AA	**1296.3**	0.0	0.0	0.0
Prostaglandin D2	351.2 -> 315.3	Cyclooxygenase	AA	**106.4**	0.0	0.0	0.0
TXB2	369.2 -> 168.9	Cyclooxygenase	AA	**792.6**	0.0	0.0	0.0
10(S),17(S)-DiHDoHE (Protectin DX)	359.2 -> 153.0	8-hydroxylation	DHA	**96.6**	0.0	0.0	0.0
14(S)-HDHA	343.2 -> 205.0	12-Lipoxygenase	DHA	**67.3**	0.0	0.0	0.0
17(S)-HDHA	343.2 -> 281.2	15-Lipoxygenase	DHA	**57.6**	0.0	0.0	0.0
7R Maresin-1	359.2 -> 250.0	12-Lipoxygenase	DHA	**100.5**			0.0
7S Maresin-1	359.2 -> 341.2	12-Lipoxygenase	DHA	**164.2**	0.0	0.0	0.0
Resolvin D1 (RVD1)	375.2 -> 141.0	12-Lipoxygenase	DHA	**112.9**	0.0	0.0	0.0
Resolvin D2 (RVD2)	375.2 -> 141.0	12-Lipoxygenase	DHA	**74.8**	0.0	0.0	0.0
**13-HODE**	**295.2 -> 277.1**	**15-Lipoxygenase**	**LA**	**566.5**	**561.1**	**79.8**	**166.5**
**13-OxoODE**	**293.2 -> 113.0**	**15-Lipoxygenase**	**LA**	**129.7**	**135.6**	**91.0**	**105.9**
**9-HODE**	**295.2 -> 277.0**	**12-Lipoxygenase**	**LA**	**586.6**	**702.8**	**218.1**	**119.3**
**9-OxoODE**	**293.2 -> 185.0**	**12-Lipoxygenase**	**LA**	**267.3**	**410.8**	**131.7**	**64.4**

AA = Arachidonic acid
DHA = Docosahexaenoic acid
LA = Linoleic acid
