# Supplementary material for: Determining the presence of asthma-related molecules and salivary contamination in exhaled breath condensate
Source: Respir Res. 2017 Apr 12;18:57. doi: 10.1186/s12931-017-0538-5 (PMC5389118; doi:10.1186/s12931-017-0538-5)
Supplement: Supplementary file 9 — Pathway analysis based on sample type. The compounds which were detected in each sample type were mapped to KEGG pathways using the online freeware pathway analysis software MBROLE. The compound names were based on database annotations using exact mass, isotope ratios and/or MSMS. Only pathways with hits ≥ 2 are listed. (PDF 278 kb) [file 12931_2017_538_MOESM9_ESM.pdf]

Additional File 9: Pathway analysis based on sample type. The compounds which were detected in each sample type were mapped to KEGG pathways using the online freeware pathway analysis software MBROLE. The compound names were based on database annotations using exact mass, isotope ratios and/or MSMS. Only pathways with hits ≥ 2 are listed.

Biological Pathway	Cohort 1: Healthy			Cohort 2: Asthma	Cohort 3: Clean EBC			
	EBC Clean	EBC Saliva	Saliva	EBC Clean	Healthy	Common Cold	Nasal Congestion	Smoker
3-Chloroacrylic acid degradation				X				
Arachidonic acid metabolism	X	X	X	X	X		X	X
Cysteine and methionine metabolism	X	X	X	X				
ABC transporters	X	X	X					
Glycine, serine and threonine metabolism	X	X	X	X		X	X	X
Glycerophospholipid metabolism	X	X	X		X	X	X	X
Phosphatidylinositol signaling system		X	X			X	X	X
Phenylalanine, tyrosine and tryptophan biosynthesis		X	X		X	X		X
Neuroactive ligand-receptor interaction	X	X	X	X				X
Aminoacyl-tRNA biosynthesis	X	X	X					X
Arginine and proline metabolism	X	X	X					
Cyanoamino acid metabolism	X	X	X					
Fc epsilon RI signaling pathway	X	X	X					X
Lysine degradation	X	X	X					
Methane metabolism	X	X	X					
Porphyrin and chlorophyll metabolism	X	X	X					
Purine metabolism	X	X	X					
Sulfur metabolism	X	X						
Ascorbate and aldarate metabolism		X	X					
Atrazine degradation		X	X					
Biosynthesis of 12-, 14- and 16-membered macrolides		X	X					
Biosynthesis of alkaloids derived from histidine and purine		X	X					
Biosynthesis of alkaloids derived from shikimate pathway		X	X					
Caffeine metabolism		X	X					
Ethylbenzene degradation		X	X					
Glucosinolate biosynthesis		X	X					X
Inositol phosphate metabolism		X	X					
Pentose and glucuronate interconversions		X	X					
Phenylalanine metabolism		X	X					
Primary bile acid biosynthesis		X	X					
Pyrimidine metabolism		X	X					
Thiamine metabolism		X	X					
Tyrosine metabolism		X	X					
Isoquinoline alkaloid biosynthesis		X						
Ubiquinone and other terpenoid-quinone biosynthesis		X						
Alanine, aspartate and glutamate metabolism			X					
beta-Alanine metabolism			X					
Biosynthesis of alkaloids derived from ornithine, lysine and nicotinic acid			X					
Biosynthesis of alkaloids derived from terpenoid and polyketide			X					
Biosynthesis of phenylpropanoids			X					
Biosynthesis of plant hormones			X					X
Glutathione metabolism			X					
Lysine biosynthesis			X			X	X	X
Glycerolipid metabolism			X			X	X	
Histidine metabolism			X					X
Nitrogen metabolism			X					
Pantothenate and CoA biosynthesis			X					
alpha-Linolenic acid metabolism					X	X	X	
Linoleic acid metabolism					X	X	X	X
